# The 100-protein NMR spectra dataset: A resource for biomolecular NMR data analysis

**DOI:** 10.1038/s41597-023-02879-5

**Published:** 2024-01-04

**Authors:** Piotr Klukowski, Fred F. Damberger, Frédéric H.-T. Allain, Hideo Iwai, Harindranath Kadavath, Theresa A. Ramelot, Gaetano T. Montelione, Roland Riek, Peter Güntert

**Affiliations:** 1 Institute of Molecular Physical Science, ETH Zurich, 8093 Zurich, Switzerland; 2 Institute of Biochemistry, ETH Zurich, 8093 Zurich, Switzerland; 3 Institute of Biotechnology, University of Helsinki, 00100 Helsinki, Finland; 4 Department of Chemistry and Chemical Biology, and Center for Biotechnology and Interdisciplinary Sciences, Rensselaer Polytechnic Institute, Troy, NY 12180 USA; 5 Institute of Biophysical Chemistry, Goethe University, 60438 Frankfurt am Main, Germany; 6 Department of Chemistry, Tokyo Metropolitan University, Hachioji, 192-0397 Tokyo Japan

**Keywords:** Solution-state NMR, Biophysical chemistry

## Abstract

Multidimensional NMR spectra are the basis for studying proteins by NMR spectroscopy and crucial for the development and evaluation of methods for biomolecular NMR data analysis. Nevertheless, in contrast to derived data such as chemical shift assignments in the BMRB and protein structures in the PDB databases, this primary data is in general not publicly archived. To change this unsatisfactory situation, we present a standardized set of solution NMR data comprising 1329 2–4-dimensional NMR spectra and associated reference (chemical shift assignments, structures) and derived (peak lists, restraints for structure calculation, etc.) annotations. With the 100-protein NMR spectra dataset that was originally compiled for the development of the ARTINA deep learning-based spectra analysis method, 100 protein structures can be reproduced from their original experimental data. The 100-protein NMR spectra dataset is expected to help the development of computational methods for NMR spectroscopy, in particular machine learning approaches, and enable consistent and objective comparisons of these methods.

## Background & Summary

The fundamental data produced by biomolecular NMR spectroscopy are multidimensional NMR spectra. All NMR-based information is derived from these spectra, generally by chemical shift assignment followed by a variety of analyses yielding information on the structure, dynamics, interactions, and mechanisms of proteins and other biomolecules^1^. Despite the central importance of NMR spectra, these are so far not systematically archived in public databases and are therefore not readily available to other researchers. This contrasts with the situation for certain derived data, namely chemical shift assignments and three-dimensional structures, which are archived since many years and abundant in well-established databases, i.e., the Biological Magnetic Resonance Data Bank (BMRB)^2^ for chemical shifts and the Protein Data Bank (PDB)^3^ for protein structures. Although the BMRB does support deposition of time-domain NMR data and peak lists, this feature of the data archive is not extensively used by the community. Some standardized data sets are collected in a few study-specific data repositories^4,5^ and recommendations for organizing such data have been developed^6^. However, there is not yet a community-wide effort to collect and validate the NMR time-domain data that support the BMRB and PDB archives of derived chemical shifts and structures.

The fact that NMR spectra, which underlie protein structure determinations by NMR, are in general not available hampers NMR studies, particularly methods development for NMR data analysis. Due to a lack of large-scale primary NMR datasets, NMR data analysis methods are generally developed on the basis of NMR data from just a few proteins that happen to be in the researchers’ hands. This presumably results in sub-optimally parametrized methods, loss of statistical significance in validation, and, importantly, lack of comparability with other methods, since different approaches are not evaluated on the same, standardized data. Machine learning-based methods that require large training and testing datasets exacerbate this problem. Hence, in machine learning large-scale benchmark datasets are standard for methods development and evaluation, e.g., for image classification^7^.

During our recent development of the machine learning-based ARTINA workflow^8^ and the NMRtist webserver^9^ for automated NMR peak picking, chemical shift assignment, and protein structure determination we became acutely aware of the lack of benchmark data sets that include the complete sets of spectra for the assignment and structure determination of a protein. Even though more than 20,000 chemical shift lists and about 14,000 NMR structures have been deposited in the BMRB and PDB databases, respectively, it was a cumbersome task, requiring months of manual work, to collect and standardize the comparatively small number of spectra that had been used previously for the structure determination of just 100 of these proteins (Supplementary Table 1, Supplementary Table 2). Currently, these data are organized differently in every NMR research group and often even by every individual within a group with annotations and naming conventions of the spectra being variable in format and often even internally inconsistent or ambiguous. This makes it difficult to collect, standardize and annotate NMR datasets obtained from individuals and groups. We therefore developed software which assists in this process and allowed us to integrate spectra from different sources.

Here we present a 100-protein NMR spectra dataset that comprises 1329 2D–4D NMR spectra (Supplementary Table 3, Supplementary Table 4), as well as associated reference data (chemical shift assignments, distance restraints, and protein structures collected from the BMRB and PDB databases) and derived data (e.g., expected list of peaks calculated for each NMR spectrum). Our dataset allows to recapitulate the entire process of structure determination with NMR spectroscopy for 100 proteins, reproducing all steps from visual analysis of raw spectra to the calculation of the protein structure. The spectra data is standardized and has been converted to the most popular formats in the field, such as UCSF Sparky^10^, NMRPipe^11^ and XEASY^12^.

Primary NMR data may either be stored as time-domain (free induction decays, FIDs) or frequency-domain (spectra) data. While the two are essentially equivalent, unknown details of the data processing (for instance, apodization functions, baseline correction, etc.) impede a strict mathematical one-to-one correspondence or invertibility. The ARTINA data set provides spectra rather than time-domain data because (i) time-domain data was not available for many of the 100 proteins, (ii) NMR data analysis for assignment, structure determination, and other investigations works almost always in the frequency domain, and (iii) time-domain data needs to be accompanied by a comprehensive set of parameters (parameters of the measurement on the spectrometer, and parameters/scripts for different data processing software packages) in order to reproduce the corresponding spectrum, which poses additional challenges for standardization.

**Fig. 1:**
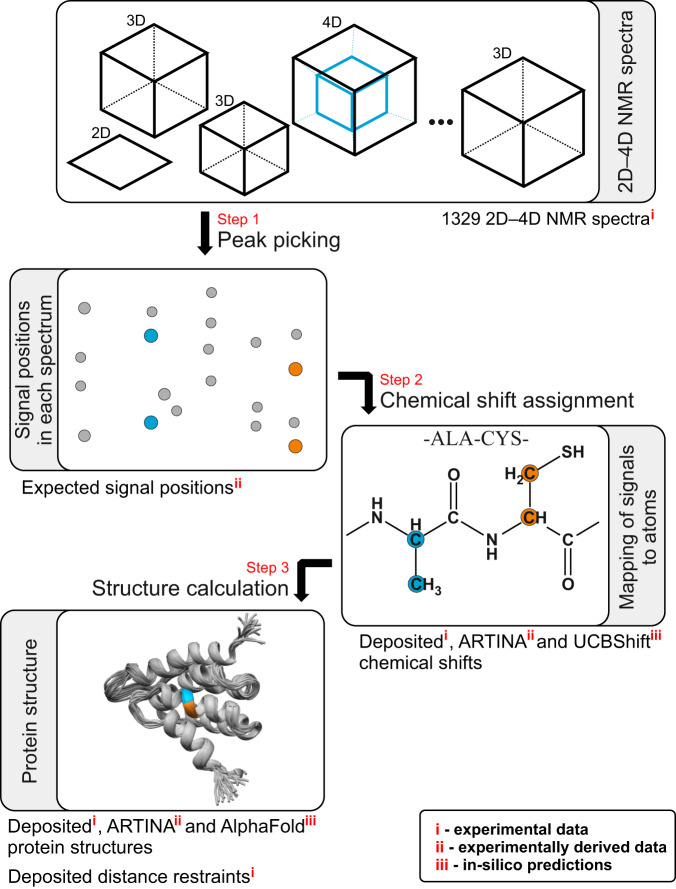
NMR spectra analysis workflow associated with the Dataset. Each protein record contains the protein sequence and a set of 2D–4D spectra, which undergo visual spectrum analysis (peak picking), yielding the coordinates of signals in the NMR spectra. Subsequently, identified signals are assigned to atoms in the protein sequence (chemical shift assignment). Assignments can then be used, for instance, to obtain interatomic distance restraints and to determine the three-dimensional protein structure. The Dataset documents all steps of this analysis for 100 proteins with (i) experimental, (ii) experimentally derived, and (iii) in-silico data, as indicated in the diagram.

The 100-protein NMR spectra dataset (in the following referred to as ‘the Dataset’) covers a wide range of proteins typically studied with NMR spectroscopy, ranging from small domains (35 residues, 4.1 kDa) to larger systems (175 residues, 20.3 kDa). All these proteins have well-defined tertiary structure, but their sequences may also include unstructured regions (Fig. 1).

To the best of our knowledge, the Dataset constitutes the largest standardized source of NMR primary data. In the past, similar datasets were used, but they consisted of fewer spectra and frequently did not cover all steps of protein structure determination with NMR. One of the prominent examples was the community-wide Critical Assessment of Automated Structure Determination by NMR (CASD-NMR) experiment^5,13^, for which 20 NMR spectra (NOESY type only) of 10 protein targets were provided. The benchmark presented here contains therefore 10 times more protein targets and over 65 times more spectra than the CASD-NMR dataset that was prepared to host this popular event in the NMR community. Other reference points are provided by publications presenting new computational methods in the field. For example, the FLYA algorithm^14^ was originally evaluated with 3 proteins (46 spectra), WaVPeak^15^ with 8 proteins (32 spectra), and PICKY^16^ with 8 proteins (32 spectra).

Further insights into the Dataset come from considering the NMR structure determination process (Fig. 1). It starts from a set of NMR spectra in the frequency domain, consisting of different experiment types recorded for the same protein. In total, 25 distinct experiment types are present among the 1329 NMR spectra in the Dataset. Both in the conventional manual and the automated ARTINA protocol, these spectra undergo visual analysis to identify signals coordinates. As reference information for this step of analysis, we provide for each benchmark spectrum lists of expected peak positions back-calculated from knowledge of the magnetization transfer rules, the protein sequence, the ground truth chemical shift deposited in the BMRB, and (for NOESY) the protein structure deposited in the PDB. In the next step of the analysis, identified signals are mapped to atoms in the protein sequence, yielding list of chemical shifts. Finally, interatomic distance restraints and possibly other conformational restraints are collected, from which the protein structure is calculated. In the Dataset, we provide lists of manually identified chemical shifts, distance restraints, and the protein structure in a standardized form, extracted from the public PDB and BMRB repositories. This non-primary experimental data is complemented with derived annotations, including in-silico predictions, such as AlphaFold^17^ structure models and UCBShift^18^-predicted chemical shift lists, that facilitate the development of hybrid approaches for experimental data analysis.

## Methods

### Spectra data acquisition

To collect NMR spectra, we explored four data sources. First, we implemented specialized crawler software that systematically scanned the FTP server of the BMRB database, extracting files relevant for this project, i.e., either spectra files with frequency-domain data or time-domain data accompanied by processing scripts. These files were converted to data formats available in the Dataset without any alteration of the original data. We did not perform any additional spectra processing steps to improve the deposited data. Time-domain data was used only if no frequency-domain data was available and if the author of the original measurement had uploaded the NMRpipe^11^ processing script to the BMRB. Notably, a large portion of the data extracted by our BMRB crawler software had been measured within the Northeast Structural Genomics Initiative (NESG)^19^.

The above data acquisition channel was complemented with a volunteer data upload initiative. To this end, we established a temporary web portal that allowed researchers to upload their published NMR spectra, thereby contributing to the Dataset. Guidelines for data submission were provided for quality and consistency, and each uploaded dataset was manually verified before inclusion in the Dataset. Finally, the diversity of the benchmark dataset was enhanced by including measurements of the authors and spectra recorded in their collaboration network. In total, the data originates from 13 different last authors of PDB depositions (Supplementary Table 2), including 78 referring to the Northeast Structural Genomics Initiative (NESG)^19^ and 3 from the RIKEN Structural Genomics/Proteomics Initiative (RSGI)^20^.

A broad range of high-field NMR instruments (600–950 MHz) and experimental setups was used to measure the spectra in the Dataset. This ensures that the dataset is representative for equipment used in the field and increases the chance that computational approaches developed with its support will generalize well to future applications. It is worth mentioning that the Dataset does not include any proprietary data, and all spectra were either publicly available, shared voluntarily by researchers, or generated by the authors and their collaborators. Supplementary Table 2 provides, for each benchmark protein, the names of the authors of the original measurements and the manual structure determination, the literature reference (if available), NMR instruments used to acquire the spectra, etc. Manually determined shift assignments and structures are available from the BMRB and PDB for 97 out of 100 proteins. Papers describing the manual structure determination have been published for 34 proteins^21–49^, and NOE and possibly other restraints for the manual structure determination have been deposited in the PDB for 94 of the 100 proteins.

### Spectra data standardization

A common challenge in NMR data analysis are systematic shifts, which may originate from different relative referencing of spectra. In manual data analysis, it constitutes only a minor obstacle, because two differently referenced spectra are consistent with each other, and their expected signal positions are merely shifted by constant. Popular software packages allow for the correction of these systematic shifts while visualizing spectra, solving the problem entirely from the user perspective, but leaving the source data intact. Therefore, we had to reference all 1329 2D–4D NMR spectra to their corresponding BMRB/PDB depositions. For each spectrum we back-calculated coordinates of expected signals from the corresponding BMRB chemical shift list and PDB structure. Afterwards we determined the optimal reference shifts for each spectrum by maximizing the sum of the absolute intensities of the spectrum at the expected peak positions, $o\left({\boldsymbol{w}}\right)={\sum }_{n}\left|s\left({{\boldsymbol{p}}}_{n}+{\boldsymbol{w}}\right)\right|$, where the summation runs over all peaks back-calculated from BMRB shifts and the PDB structure, ***p***_*n*_ denotes the position of the *n*-th expected peak in the spectrum, the vector ***w*** collects the systematic spectrum reference shifts in each dimension, and *s*(∙) is the intensity of the spectrum at the given position. Since the reference shifts ***w*** are typically small and the digital resolution of the spectrum is finite, the optimal reference shifts could be determined by a simple exhaustive search procedure with a finer spacing than the digital resolution of the spectrum. The correctness of the referencing was manually verified for each spectrum, as described in the Technical Validation section below.

Subsequently, we unified the spectra properties, following the standards of the CYANA/FLYA library^14^. Experiment types and axis labels in each spectrum file were set as specified in Supplementary Table 3 and the overall spectrum intensity was normalized by rescaling each spectrum with a constant to obtain an approximate median value of 100 for the absolute intensities of the scaled spectrum data points^50^.

### Standardization and preparation of other data

For each spectrum included in the Dataset, we calculated signals that are expected to be observed^14,51^ based on the reported chemical shift assignments deposited in the BMRB and the structures in the PDB. Lists of generated cross-peaks are available in three different variants: expected peaks, expected assigned peaks folded and expected assigned peaks unfolded. The first list stores the assignments of all signals that are expected based on the pulse sequence, the protein sequence and, in case of NOESY spectra, the PDB structure^14^, regardless of whether their chemical shift assignment is available in the BMRB or not. This list does not contain peak coordinates. The second and third variant of back-calculated peak lists contain folded or unfolded signal coordinates together with atoms assigned to each dimension of every cross-peak. Cross-peaks with missing assignments in the BMRB, and therefore unknown signal coordinates in the spectrum, are not included in these two peak lists. Peaks and their positions are therefore set according to the chemical shift assignments deposited in the BMRB; these are not the experimental peak lists that have been deposited, for only a small fraction of all spectra, in the BMRB by the original depositors of the data. Lists of expected assigned peaks were prepared according to the formal magnetization transfer rules in the CYANA/FLYA library^14^ (Supplementary Table 3). For spectra with purely through-bond magnetization transfer, the peak lists were generated using only the protein sequence as input, whereas for NOESY spectra the manually determined structure from the PDB was used in addition to obtain NOESY cross peaks for short ^1^H-^1^H distances. If an NOE involved groups of degenerate ^1^H shifts, e.g., for methyl groups, the *r*^–6^-summed distance^52^ was used. These generated peak lists were used to match the chemical shift referencing of the spectra and to verify data consistency.

Each protein record in the Dataset contains reference chemical shift assignments, which were acquired from the BMRB database, as well as a reference protein structure, acquired from the PDB database. To ensure consistency of the reference data, we unified the residue numberings in all data files to match those of the BMRB deposition.

To further facilitate the use of the Dataset for the development and evaluation of computational approaches in protein NMR spectroscopy, we prepared AlphaFold^17^ and UCBShift^18^ predictions of structures and chemical shifts. The latter comprises H^N^, H^α^, H^β^, C^α^, C^β^, C’, and N shifts predicted using the AlphaFold structure and can serve as prior information for computational approaches under development.

## Data Records

**Fig. 2:**
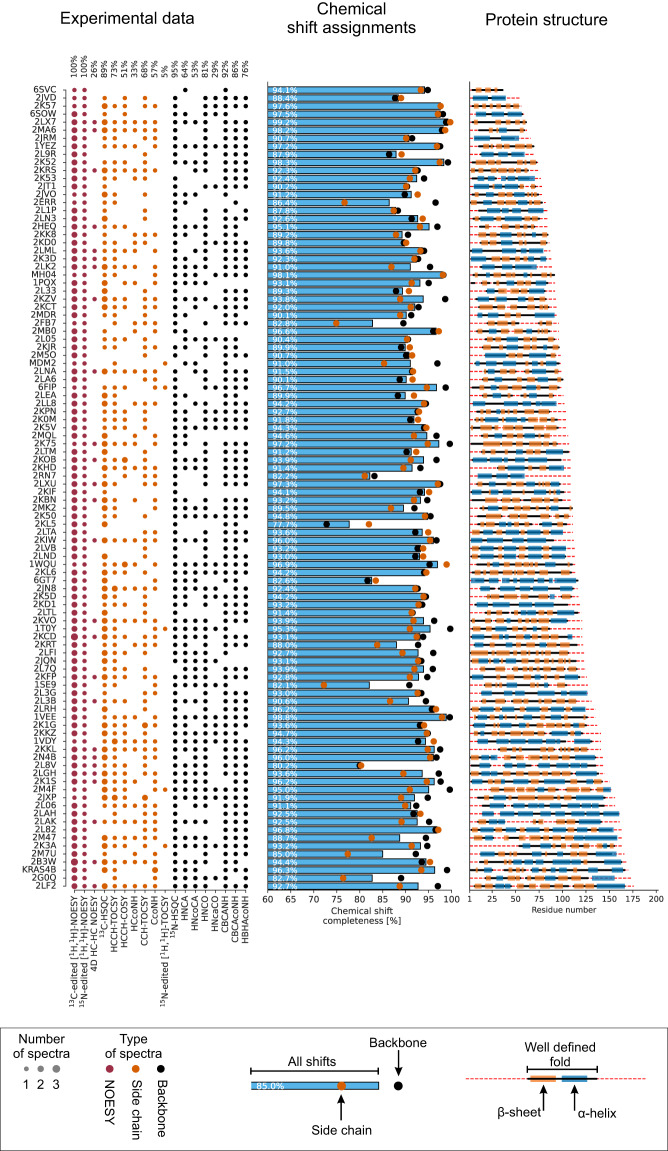
Overview of the Dataset comprising 100 proteins and 1329 spectra. Proteins are ordered by sequence length and identified by their PDB code or abbreviated name if not deposited in the PDB (left). The left panel shows the spectra available for each protein. Where multiple spectra are available for a given spectrum type, they typically have been acquired by separate measurement of aliphatic and aromatic ^13^C nuclei or with H_2_O and D_2_O solvent. The middle panel shows the completeness of the chemical shift assignments deposited in the BMRB. The right panel shows secondary structure elements and well-defined regions plotted versus the residue number. Well-defined regions, which are used for all RMSD calculations, were determined by CYRANGE^66^.

Each of the 100 protein records in the Dataset (Supplementary Table 1, Supplementary Table 2) is comprised of the data specified in Tables 1–5. It is available from the long-term ETH Research Collection^53^ and from https://nmrdb.ethz.ch. Data for each protein is stored in a directory entry that is named with the PDB code or with an abbreviated protein name if the structure of the protein has not been deposited in the PDB. Figure 2 provides an overview of the dataset, including, for each of the 100 proteins, the spectra available, the chemical shift assignment completeness from the BMRB, and the sequence length and secondary structure composition from the PDB.

**Table 1:** Files for each multidimensional NMR spectrum in the Dataset.

File name (*X* = spectrum type)	Content
*X*.ucsf	Spectrum in UCSF Sparky format
*X*.pipe	Spectrum in NMRpipe format
*X*.3D.16, *X*.3D.param	Spectrum in XEASY format with associated parameter file
*X*_projection_w*ij*.jpg	Contour plot of projection for dimensions *i* and *j*
*X*_projection_w*ij*_with_peaks.jpg	Contour plot of projection for dimensions *i* and *j*, with expected peaks
*X*_exemplary_layer_000*i*_with_peaks.jpg	Contour plots of 3 exemplary layers (*i* = 0, 1, 2); for 3D spectra

**Table 2:** General files for proteins in the Dataset.

File name	Content
sequence.fasta /.seq	Amino acid sequence in FASTA and CYANA format
manual_structure.cif /.pdb	Reported structure from PDB in mmCIF and PDB format
bmrb.str	Reported chemical shifts from BMRB in NMR-STAR format
manual_shift_list.nef /.prot	Reported chemical shifts in NEF^65^ and XEASY format

**Table 3:** Files for peak lists in the Dataset.

File name (*X* = spectrum type)	Content
*X*_expected.list /.peaks	All expected peaks in Sparky and XEASY format (assignments, not positions)
*X*_assigned.list /.peaks	Assigned peaks in Sparky/XEASY format (positions from deposited BMRB shifts)
*X*_assigned_folded.list /.peaks	Assigned peaks in Sparky/XEASY format, folded

**Table 4:** Derived data files for proteins in the Dataset.

File name	Content
ARTINA.cif /.pdb	Structure determined by ARTINA in mmCIF and PDB format
ARTINA_all.nef /.prot	All assignments determined by ARTINA in NEF and XEASY format
ARTINA_strong.nef /.prot	Strong assignments^14^ determined by ARTINA in NEF, XEASY format
AlphaFold.cif /.pdb	AlphaFold structure models in mmCIF and PDB format
UCBShift_AlphaFold.csv /.nef /.prot	Shifts predicted by UCBShift in CSV, NEF, XEASY format
UCBShift_AlphaFold_referenced.nef /.prot	Re-referenced shifts from UCBShift in NEF and XEASY format

**Table 5:** Files for the recalculation of structures with CYANA using the manually determined restraints deposited in the PDB.

File name	Content
sequence.seq	Amino acid sequence in CYANA format
protein.seq	Sequence for structure calculation comprising the residues with coordinates in pdb.cif, renumbered according to sequence.seq
pdb.cif /.pdb	Manually determined structure from PDB in mmCIF and PDB format
pdb.mr	Restraints file from PDB in XPLOR, CYANA, or AMBER format
pdb.nef	Restraints and shifts file from PDB, if available (not used for recalculation)
bmrb.str	Manually determined chemical shifts from BMRB in NMR-STAR format
ref.cif /.pdb	Reference structure obtained from pdb.cif in mmCIF and PDB format
ref.prot	Reference chemical shifts obtained from bmrb.str in XEASY format
ref.nef	Distance and torsion angle restraints from pdb.mr in NEF format
noe.upl	NOE upper distance limits from pdb.mr in CYANA format
hbond.upl /.lol	Hydrogen bond upper and lower distance bounds from pdb.mr in CYANA format
angle.aco	Torsion angle restraints from pdb.mr in CYANA format
ref.ovw	NMR and van der Waals restraint violations, ref.nef vs. ref.cif structure
refdat.ovw	NMR restraint violations, ref.nef vs. ref.cif structure
cyana.cif /.pdb	Structure obtained by CYANA from ref.nef restraints in mmCIF and PDB format
cyana.ovw	NMR and van der Waals restraint violations, ref.nef vs. cyana.cif structure
N15NOESY_ref.peaks	^15^N-edited [^1^H-^1^H] NOESY peak list simulated by CYANA using ref.cif structure and ref.nef shifts
C13NOESY_ref.peaks	^13^C-edited [^1^H-^1^H] NOESY peak list simulated by CYANA using ref.cif structure and ref.nef shifts
init.cya	CYANA initialization script
rmsdrange.cya	Residue range for RMSD calculation
CALC.cya	CYANA structure calculation script

For each protein, the subdirectory ‘spectra’ contains the multidimensional NMR spectra, each represented with the data listed in Table 1. All spectra are named by the spectrum type according to FLYA conventions^14^ (Supplementary Table 3), possibly followed by tags @ALI or @ARO to indicate that the spectrum contains only aliphatic or aromatic ^13^C signals, respectively.

**Fig. 3:**
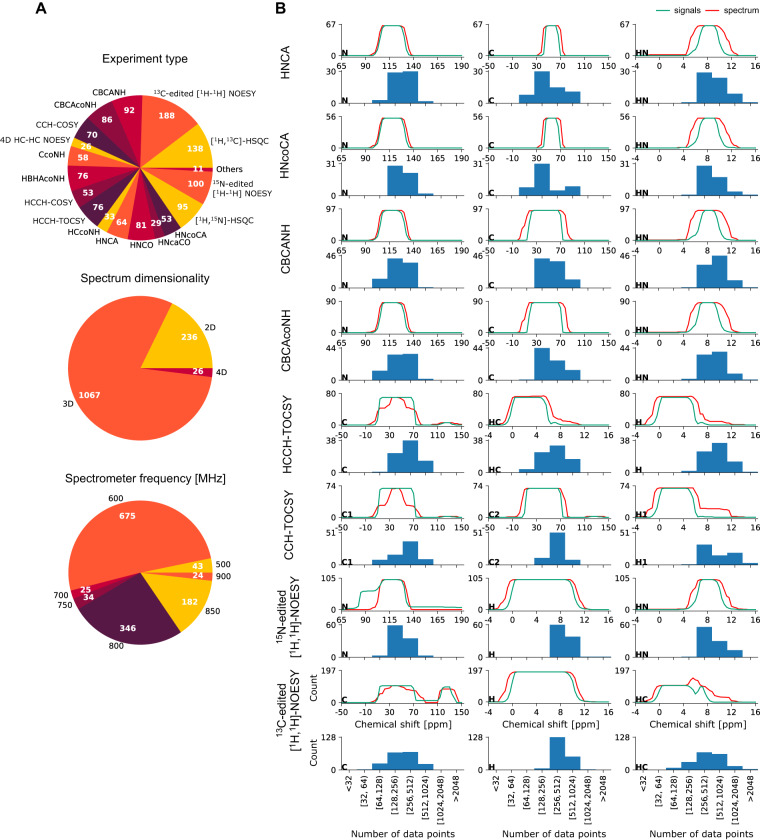
Statistics for data records in the Dataset. **(a)** Distribution of experiment types, spectrum dimensionality (2D–4D), and spectrometer frequency. **(b)** Distribution of number of data points and chemical shift ranges (ppm) across different dimensions for common 3D experiment types in the Dataset (Supplementary Table 4). For each spectrum type, the bottom row features histograms that represent the number of spectra with the specified number of data points in the given dimension, as indicated by the dimension label in the lower left corner. The upper row provides information about the chemical shift range in the spectrum file and the distribution of expected peaks in each dimension. The red line gives the number of spectra for which a given chemical shift value falls within the experimental spectral width in the given dimension. Similarly, the green line represents the number of spectra for which a given chemical shift value coincides (within tolerance) with at least one expected peak position based on the (unfolded) chemical shift assignments from the BMRB. Where the green line exceeds the red line, it indicates that folding is typically applied along that spectral axis.

A complete list of the 1329 spectra is given in Supplementary Table 4. Statistics about spectra and assignments are given in Fig. 3. About 80% of the spectra are three-dimensional, 18% are 2D,and 2% 4D (all HC-HC NOESY experiments). The dataset includes 3D ^13^C-edited [^1^H-^1^H] NOESY and ^15^N-edited [^1^H-^1^H] NOESY spectra for all 100 proteins, and for each protein the complete set of spectra of various types that was used for the original backbone and sidechain chemical shift assignment deposited in the BMRB. About 51% of the spectra were recorded at 600 MHz ^1^H frequency, 3% at lower frequency (500 MHz), 4% at 700–750 MHz, and 42% at higher frequencies of 800–900 MHz.

Another factor that plays an important role in the development of computational approaches for NMR spectra analysis are the number of data points in each dimension of the spectrum and the type of aliasing (folding) used in the experiment. Such information can be extracted directly from data provided in the Dataset. Figure 3 presents a summary for eight popular experiment types. Folding is typically applied to a ^13^C dimension in sidechain assignment spectra (axis labels C for HCCH-TOCSY or C1 for CCHTOCSY) and the ^15^N or ^13^C dimension in 3D NOESY experiments. Most triple-resonance NMR data used for determining backbone resonance assignments are collected using the constant-time frequency labeling approach^54^, which avoids the need for folding to improve digital resolution in these indirect dimensions.

NMR measurements are complemented with reference data, stored in the ‘others’ subdirectory, comprising the protein sequence, the manually determined structure from the PDB database, and the manually determined chemical shift assignments from the BMRB (Table 2).

**Fig. 4:**
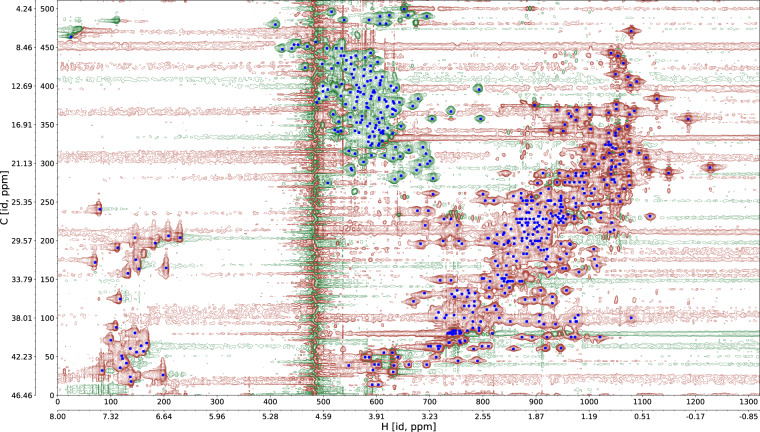
Contour plot of a [^1^H,^13^C]-HSQC spectrum for the protein 1VDY. Positions of peaks back-calculated from the chemical shifts deposited in the BMRB are indicated by blue crosses.

Expected peak lists for each spectrum are in the subdirectory ‘peak_lists’ (Table 3). These are not experimental peak lists (which are not available from BMRB or PDB depositions for most spectra) but lists of peaks expected based on sequence and experiment type (see Methods). As an example, the back-calculated peak list for the [^1^H,^13^C]-HSQC spectrum of the protein with PDB code 1VDY is overlayed on the spectrum in Fig. 4.

Additional derived data are available in the subdirectory ‘others’ (Table 4). Files ARTINA*.* are the result of fully automated spectra analysis^8^. These comprise chemical shift assignments for all atoms and the subset of “strong” (reliable) assignments^14^ as well as the three-dimensional structures obtained by ARTINA using only sequence and spectra as input. Additionally, we included, in each data record, structure predictions by AlphaFold^17^ and chemical shift predictions by UCBShift^18^ using the AlphaFold structure as input.

The subdirectory ‘recalc’ contains the manually determined conformational restraints deposited in the PDB, if available, and data from a recalculation of the PDB structure with CYANA^52,55^ using the available NOE distance restraints, hydrogen bond distance restraints, and torsion angle restraints (Table 5).

## Technical Validation

Data stored in the Dataset underwent rigorous qualitative and quantitative validation, as well as consistency checks, which verified that different data modalities (i.e., chemical shift lists obtained from the BMRB, and spectra acquired on-line from the web portal) are consistent with each other.

The first data validation procedure involved the spectra files and expected assigned peaks lists stored in each Dataset record. For each multidimensional spectrum, we calculated all 2D projections of the spectral data along one (3D spectra) or two (4D spectra) spectrum axes. Afterwards, we visualized each projection as a contour plot overlaid with the expected peaks back-calculated from the reference chemical shifts in the BMRB and the structure in the PDB (Fig. 4; Supplementary Fig. 1). In this way we prepared 3593 data visualizations, which were inspected manually and are included in the Dataset together with the source spectra files (Table 1). This verification procedure ensured consistency between raw spectral data, corresponding reference data (shift lists, protein structure), and derived data (expected peak lists). All spectra in the Dataset agree well with the corresponding reference data from BMRB/PDB. Subsequently, we performed a similar round of qualitative evaluation using randomly selected 2D planes (3279 in total) instead of projections from the multidimensional spectra (Supplementary Fig. 2).

**Fig. 5:**
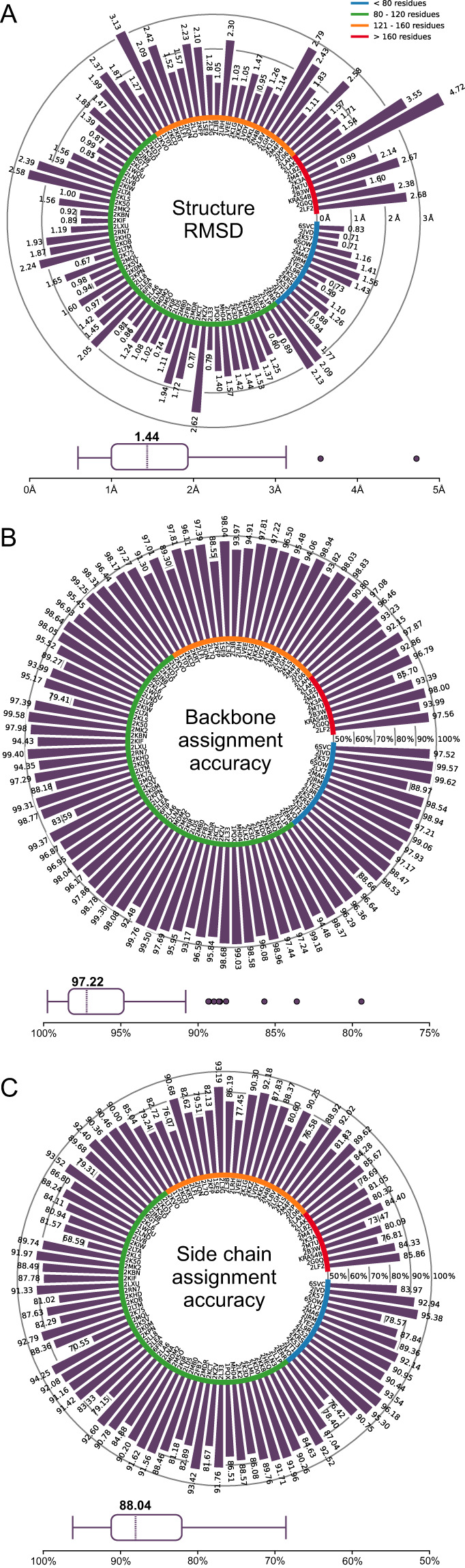
Data validation by automated spectrum analysis with ARTINA. The three panels show, for 100 proteins, the backbone RMSD between the ARTINA structure and the NMR structure deposited in the PDB, as well as the accuracy of the backbone and sidechain assignment by ARTINA relative to the assignments deposited in the BMRB. Proteins presented in bar plots are sorted clockwise by sequence length. Box plots present the distribution and the median of these quantities.

In quantitative data validation, we used as input 1329 2D–4D spectra to automatically reproduce 100 protein structures and assignments with the ARTINA algorithm^8^. The results (Fig. 5) indicate good agreement between the automated spectra analysis and the manual annotations deposited in the BMRB and PDB databases. Consistency of the ARTINA result, obtained exclusively from spectra and sequence, was confirmed by 91.36% identical chemical shift assignments and a median RMSD of 1.44 Å (Supplementary Table 5) with respect to the manually determined chemical shift assignments and structures deposited in the BMRB and PDB, respectively^8^.

The accuracy of the AlphaFold models was validated by the RMSD to the structures deposited in the PDB (Supplementary Table 5). The median accuracy of the AlphaFold models on the benchmark dataset is 0.96 Å for the backbone atoms. There are only four proteins with RMSD above 2 Å and all RMSDs are below 3 Å. Excellent agreement between AlphaFold models and NMR NOESY data, including for some of the proteins in the Dataset, has been described elsewhere^56,57^.

Validations of the standardized manually determined conformational restraints deposited in the PDB were performed by recalculating the structures using CYANA. Structure recalculation could be performed for 96 out of 100 proteins for which more than 2 manually determined NOE distance restraints per residue are available from the PDB. Statistics of the reported conformational restraints and the structures deposited in the PDB or recalculated from this data are given in Supplementary Table 6. Consistency of the interpretation of the restraints and the recalculation of the structures (e.g., regarding residue numbering, atom nomenclature, and handling of degenerate or non-stereospecifically assigned atoms) is confirmed by low CYANA target function values for the recalculated structures (0.01–14.27 Å^2^, median 0.28 Å^2^) and small backbone RMSD values between the deposited and recalculated structures (0.25–1.98 Å, median 0.79 Å).

The above analysis confirmed consistency of all data modalities, namely NMR spectra, expected peak lists, manual chemical shift assignments, manually solved protein structures, and AlphaFold predictions. This agrees with our previous study^8^, in which the same spectra dataset was used to develop ARTINA method.

## Usage Notes

Since NMR spectra are the fundamental result of NMR measurements, the Dataset facilitates the development of computational methods and software packages for protein NMR, covering a broad range of downstream tasks, ranging from automated visual spectrum analysis to hybrid approaches for assignment and protein structure determination that merge in-silico predictions (e.g., AlphaFold) with experimental data^58,59^, as well as testing of software for manual NMR data analysis.

We believe that one of the primary applications of the Dataset will involve the development of new computational approaches to classical problems in NMR spectroscopy, such as automated peak picking, chemical shift assignment, structure determination, as well as spectrum quality enhancement. The Dataset opens new avenues in these areas by providing enough data for the training of deep learning architectures, which have so far been used rarely in NMR spectroscopy, mainly because of the lack of adequate training/benchmark data. In addition, the Dataset provides a means to establish links between core machine learning problems and protein NMR, such as the adaptation of well-performing model architectures to NMR spectroscopy.

Likewise, the Dataset may find its application in the development and testing of software packages designed for manual analysis, storage, or validation of NMR data. In essence, the Dataset serves as a robust testing ground, enabling software developers to evaluate and refine their tools, ultimately contributing to the advancement of their software packages and NMR research generally.

The Dataset also opens new opportunities for comparative studies of existing methods in NMR spectroscopy. By providing a large, comprehensive, and standardized dataset, it allows for systematic and unbiased evaluations of current methods in terms of accuracy, efficiency, and robustness. It enables the identification of potential limitations and areas for improvement in current methodologies. This, in turn, can drive the refinement and optimization of existing tools, ultimately contributing to the advancement of computational approaches in the field of protein NMR spectroscopy.

After the recent breakthroughs in deep learning-based protein structure prediction, the research community seeks hybrid approaches that combine in-silico methods with experimental data^60^. The Dataset facilitates these studies by providing primary experimental data as well as ground truth protein folds and the reference structure quality, obtained by automated data analysis with the ARTINA method.

Finally, the Dataset can be used for educational purposes, serving as a resource for both students and researchers who seek to deepen their understanding of NMR spectroscopy and computational methods for protein structure determination. The comprehensive nature of the Dataset allows for a wide range of applications, from basic spectrum analysis to advanced structure determination exercises. The Dataset can serve as a basis for practical assignments and projects in courses related to bioinformatics, cheminformatics, and structural biology.

In summary, the Dataset addresses a crucial gap in biomolecular NMR research by providing a large-scale, standardized set of spectra, reference data, and derived annotations for 100 proteins. It is significantly larger and more comprehensive than previous datasets that were typically compiled ad hoc for the development of specific methods or for a smaller number of spectral data types^4,5,61^. The Dataset not only enables reproduction of the entire structure determination process, but also facilitates the development and evaluation of new computational and machine learning-based approaches, holding the promise of becoming a crucial asset for future computational methods development in NMR spectroscopy.

It may be surprising that more than 30 years after the initial development of NMR protein structure determination, and with currently about 14,000 NMR-derived structures in the PDB and a similar number of chemical shift assignment datasets in the BMRB, no comparable collection of primary, time- or frequency-domain NMR data exists. In contrast, it has long been universal practice in X-ray crystallography to deposit the primary data, i.e., the structure factors, along with the protein structure derived from it. One reason for the scarcity of NMR primary data depositions (available for < 0.5% of the NMR structures in the PDB) is that this data is significantly more complex than the structure factors in X-ray crystallography: a protein structure determination requires a series of multidimensional spectra of different types, a variety of binary encoded formats and variants thereof are in use for time- and frequency-domain NMR data, the data size is typically 2–4 orders of magnitude larger than for structure factors, and, significantly, the connection between spectra and structure is much less direct than in X-ray crystallography, where the degree of agreement between structure factors and the structure model derived from them can be quantified readily, e.g., by R-factors. In the absence of a fully automated method such as ARTINA, any verification of an NMR protein structure from spectra required a significant amount of manual work and would therefore have been undertaken only rarely even if the primary data were available. With machine learning-based methods like ARTINA, the situation has changed: NMR analyses of many proteins can now be done efficiently, and, at the same time, the demand for large amounts of standardized training data has become acute. While some solutions to this data management challenge have been proposed^6,62–64^, none have been adopted by the wider biomolecular NMR community. The Dataset constitutes a significant step towards solving this problem. In the medium and long term, however, it would be best to establish the simultaneous deposition of primary NMR data (including time-domain FID data, frequency-domain spectra, and peak lists) together with chemical shift assignments and structures in public databases as a standard in structural biology. The necessary standardization and validation will likely require the public databases to provide seamless automated procedures such as those that have been developed, in part, for the preparation of the 100-protein NMR spectra data set.

### Supplementary information

Supplementary Information

## Data Availability

No custom code is required to access and use the ARTINA spectra database.
