# Supplementary material for: The 100-protein NMR spectra dataset: A resource for biomolecular NMR data analysis
Source: Sci Data. 2024 Jan 4;11:30. doi: 10.1038/s41597-023-02879-5 (PMC10767026; doi:10.1038/s41597-023-02879-5)
Supplement: Supplementary file 1 — Supplementary Information [file 41597_2023_2879_MOESM1_ESM.pdf]

Supplementary Information

The 100-protein NMR spectra dataset: A resource for biomolecular NMR data analysis

Piotr Klukowski[1], Fred F. Damberger[2], Frédéric H.-T. Allain[2], Hideo Iwai[3], Harindranath Kadavath[1], Theresa A. Ramelot[4], Gaetano T. Montelione[4], Roland Riek[1], Peter Güntert[1,5,6]

1. Institute of Molecular Physical Science, ETH Zurich, 8093 Zurich, Switzerland
2. Institute of Biochemistry, ETH Zurich, 8093 Zurich, Switzerland
3. Institute of Biotechnology, University of Helsinki, 00100 Helsinki, Finland
4. Department of Chemistry and Chemical Biology, and Center for Biotechnology and Interdisciplinary Sciences, Rensselaer Polytechnic Institute, Troy, NY, 12180, USA
5. Institute of Biophysical Chemistry, Goethe University, 60438 Frankfurt am Main, Germany
6. Department of Chemistry, Tokyo Metropolitan University, Hachioji, 192-0397 Tokyo, Japan

corresponding author(s): Piotr Klukowski (piotr.klukowski@phys.chem.ethz.ch), Roland Riek (roland.riek@phys.chem.ethz.ch), Peter Güntert (peter.guentert@phys.chem.ethz.ch)

Contents

Supplementary Table 1. Proteins and spectra in the 100-protein NMR spectra dataset

Supplementary Table 2. Metadata for PDB reference structures in the 100-protein NMR spectra dataset

Supplementary Table 3. Spectrum types, axis labels, and expected peaks for the spectra in the 100-protein NMR spectra dataset

Supplementary Table 4. Spectra in the 100-protein NMR spectra dataset

Supplementary Table 5. Accuracy of AlphaFold models and ARTINA structures

Supplementary Table 6. Structure recalculation with CYANA from deposited restraints

Supplementary Fig. 1. Projections of the HNCA spectrum of the protein 1VDY

Supplementary Fig. 2. Example 2D planes of the ^{15}N-resolved [^{1}H-^{1}H] NOESY spectrum for the protein 1VDY

Supplementary Table 1. Proteins and spectra in the 100-protein NMR spectra dataset.

ID	Protein	Residues	Number of spectra			
			Backbone assignment	Side-chain assignment	NOESY	Total
1	6SVC	35	2	1	3	6
2	2JVD	54	6	3	2	11
3	2K57	55	6	6	2	14
4	6SOW	58	5	2	3	10
5	2LX7	60	7	7	4	18
6	2MA6	61	5	7	4	16
7	2JRM	65	5	4	3	12
8	1YEZ	68	8	5	3	16
9	2L9R	69	5	1	3	9
10	2K52	74	7	4	3	14
11	2KRS	74	5	7	4	16
12	2K53	76	4	4	3	11
13	2JT1	77	6	1	3	10
14	2JVO	77	4	4	2	10
15	2ERR	81	3	2	2	7
16	2L1P	83	4	2	3	9
17	2LN3	83	5	3	3	11
18	2HEQ	84	6	3	4	13
19	2KK8	84	5	4	3	12
20	2KD0	85	6	4	3	13
21	2LML	86	6	5	4	15
22	2K3D	87	6	2	4	12
23	2LK2	89	7	6	4	17
24	MH04	90	7	3	2	12
25	1PQX	91	4	5	3	12
26	2L33	91	4	3	3	10
27	2KZV	92	7	6	4	17
28	2KCT	94	6	6	3	15
29	2MDR	94	4	3	3	10
30	2FB7	95	5	4	2	11
31	2MB0	95	2	3	3	8
32	2L05	95	6	4	3	13
33	2KJR	95	5	4	3	12
34	2M5O	97	5	1	3	9
35	MDM2	97	3	2	2	7
36	2LNA	99	6	7	4	17
37	2LA6	99	5	2	3	10
38	6FIP	99	6	5	2	13
39	2LEA	100	4	3	3	10
40	2LL8	101	6	6	3	15
41	2KPN	103	7	4	3	14
42	2K0M	104	7	4	3	14
43	2K5V	104	8	4	3	15
44	2MQL	105	2	4	3	9
45	2K75	106	6	6	3	15
46	2LTM	107	5	3	3	11
47	2KOB	108	7	5	4	16
48	2KHD	108	7	7	3	17
49	2RN7	108	5	4	3	12
50	2LXU	108	5	5	4	14
51	2KIF	108	1	1	3	5
52	2KBN	109	6	5	4	15
53	2MK2	109	7	4	3	14
54	2K50	110	8	3	3	14
55	2KL5	110	3	2	3	8
56	2LTA	110	5	2	3	10
57	2KIW	111	7	7	4	18
58	2LVB	112	4	1	3	8
59	2LND	112	5	3	3	11
60	1WQU	114	8	6	2	16
61	2KL6	114	8	5	3	16
62	6GT7	115	5	3	3	11
63	2JN8	115	7	8	3	18
64	2K5D	116	7	4	3	14
65	2KD1	118	6	4	3	13
66	2LTL	119	4	3	3	10
67	2KVO	120	7	7	4	18
68	1T0Y	120	6	5	3	14
69	2KCD	120	8	6	5	19
70	2KRT	121	7	5	3	15
71	2LFI	122	5	5	3	13
72	2JQN	122	5	3	3	11
73	2L7Q	124	6	4	3	13
74	2KFP	125	7	6	4	17
75	1SE9	126	6	3	3	12
76	2L3G	126	5	3	3	11
77	2L3B	130	7	4	4	15
78	2LRH	134	5	3	3	11
79	1VEE	134	8	5	3	16
80	2K1G	136	7	7	3	17
81	2KKZ	140	7	5	3	15
82	1VDY	140	8	7	2	17
83	2KKL	140	7	7	4	18
84	2N4B	142	6	5	4	15
85	2L8V	143	7	4	4	15
86	2LGH	144	7	6	4	17
87	2K1S	149	6	6	4	16
88	2M4F	151	8	6	2	16
89	2JXP	155	8	7	2	17
90	2L06	155	6	6	3	15
91	2LAH	160	4	3	3	10
92	2LAK	160	7	6	4	17
93	2L82	162	4	2	3	9
94	2M47	163	7	7	3	17
95	2K3A	163	8	6	3	17
96	2M7U	165	5	3	3	11
97	2B3W	168	8	7	5	20
98	KRAS4B	169	6	5	3	14
99	2G0Q	173	7	2	3	12
100	2LF2	175	7	7	4	18

Supplementary Table 2. Metadata for PDB reference structures in the 100-protein NMR spectra dataset. Extracted from PDB entries (except for proteins MH04, MDM2, KRAS that lack PDB depositions).

**Protein number**	1
**PDB code**	6SVC, doi:10.2210/pdb6SVC/pdb
**BMRB code**	34432, doi:10.13018/BMR34432
**UniProt code**	Q13526, PIN1_HUMAN
**PDB Header**	PEPTIDE BINDING PROTEIN
**Protein name**	PROTEIN ALLOSTERY OF WW DOMAIN AT ATOMIC RESOLUTION: APO STRUCTURE
**Deposition date**	18.09.2019
**PDB title**	PROTEIN ALLOSTERY OF THE WW DOMAIN AT ATOMIC RESOLUTION: APO STRUCTURE
**PDB authors**	D.STROTZ, J.ORTS, M.FRIEDMANN, P.GUNTERT, B.VOGELI, R.RIEK
**Last author**	RIEK
**Reference**	ANGEW.CHEM.INT.ED.ENGL. 59, 22132 (2020), doi:10.1002/ANIE.202008734
**Reference authors**	D.STROTZ, J.ORTS, H.KADAVATH, M.FRIEDMANN, D.GHOSH, S.OLSSON, C.N.CHI, A.POKHARNA, P.GUNTERT, B.VOGELI, R.RIEK
**Reference title**	PROTEIN ALLOSTERY AT ATOMIC RESOLUTION
**Software listed**	CCPNMR, CYANA, NMRDRAW, NMRPIPE
**Spectrometer**	BRUKER (700 MHZ)

**Protein number**	2
**PDB code**	2JVD, doi:10.2210/pdb2JVD/pdb
**BMRB code**	15476, doi:10.13018/BMR15476
**UniProt code**	O31818, YNZC_BACSU
**PDB Header**	STRUCTURAL GENOMICS, UNKNOWN FUNCTION
**Protein name**	FOLDED N-TERMINAL FRAGMENT OF UPF0291 PROTEIN YNZC FROM BACILLUS SUBTILIS
**Deposition date**	18.09.2007
**PDB title**	SOLUTION NMR STRUCTURE OF THE FOLDED N-TERMINAL FRAGMENT OF UPF0291 PROTEIN YNZC FROM BACILLUS SUBTILIS. NORTHEAST STRUCTURAL GENOMICS TARGET SR384-1-46
**PDB authors**	J.M.ARAMINI, S.SHARMA, Y.J.HUANG, L.ZHAO, L.A.OWENS, K.STOKES, M.JIANG, R.XIAO, M.C.BARAN, G.V.T.SWAPNA, T.B.ACTON, G.T.MONTELIONE, NORTHEAST STRUCTURAL GENOMICS CONSORTIUM (NESG)
**Last author**	MONTELIONE
**Reference**	PROTEINS 72, 526 (2008), doi:10.1002/PROT.22064
**Reference authors**	J.M.ARAMINI, S.SHARMA, Y.J.HUANG, G.V.SWAPNA, C.K.HO, K.SHETTY, K.CUNNINGHAM, L.C.MA, L.ZHAO, L.A.OWENS, M.JIANG, R.XIAO, J.LIU, M.C.BARAN, T.B.ACTON, B.ROST, G.T.MONTELIONE
**Reference title**	SOLUTION NMR STRUCTURE OF THE SOS RESPONSE PROTEIN YNZC FROM BACILLUS SUBTILIS
**Software listed**	AUTOASSIGN, AUTOSTRUCTURE, CYANA, MOLPROBITY, NMRPIPE, PDBSTAT, PROCHECK, PSVS, SPARKY, TOPSPIN, VNMR
**Spectrometer**	BRUKER, VARIAN (800 MHZ, 600 MHZ)

**Protein number**	3
**PDB code**	2K57, doi:10.2210/pdb2K57/pdb
**BMRB code**	15825, doi:10.13018/BMR15825
**UniProt code**	Q48JU9, Q48JU9_PSE14
**PDB Header**	STRUCTURAL GENOMICS, UNKNOWN FUNCTION
**Protein name**	PUTATIVE LIPOPROTEIN FROM PSEUDOMONAS SYRINGAE GENE LOCUS PSPTO2350
**Deposition date**	25.06.2008
**PDB title**	SOLUTION NMR STRUCTURE OF PUTATIVE LIPOPROTEIN FROM PSEUDOMONAS SYRINGAE GENE LOCUS PSPTO2350. NORTHEAST STRUCTURAL GENOMICS TARGET PSR76A
**PDB authors**	D.HANG, J.A.ARAMINI, P.ROSSI, D.WANG, M.JIANG, M.MAGLAQUI, R.XIAO, J.LIU, M.C.BARAN, T.B.ACTON, B.ROST, G.T.MONTELIONE, NORTHEAST STRUCTURAL GENOMICS CONSORTIUM (NESG)
**Last author**	MONTELIONE
**Reference**	
**Reference authors**	
**Reference title**	
**Software listed**	AUTOASSIGN, CNS, CYANA, MOLMOL, MOLPROBITY, NMRPIPE, PDBSTAT, PROCHECK, PSVS, RPF(AUTOSTRUCTURE), SPARKY, TALOS, TOPSPIN
**Spectrometer**	BRUKER, VARIAN (800 MHZ, 600 MHZ)

**Protein number**	4
**PDB code**	6SOW, doi:10.2210/pdb6SOW/pdb
**BMRB code**	34430, doi:10.13018/BMR34430
**UniProt code**	Q2UW48, Q2UW48_STAAU
**PDB Header**	PROTEIN BINDING
**Protein name**	STAPHYLOCOCCAL PROTEIN A, C DOMAIN
**Deposition date**	30.08.2019
**PDB title**	NMR SOLUTION STRUCTURE OF STAPHYLOCOCCAL PROTEIN A, C DOMAIN
**PDB authors**	S.M.BACKLUND, H.IWAI
**Last author**	IWAI
**Reference**	MOLECULES 2, 6 (2021), doi:10.3390/MOLECULES26030747

**Reference authors**	H.A.HEIKKINEN, S.M.BACKLUND, H.IWAI
**Reference title**	NMR STRUCTURE DETERMINATIONS OF SMALL PROTEINS USING ONLY ONE FRACTIONALLY 20% 13 C- AND UNIFORMLY 100% 15 N-LABELED SAMPLE
**Software listed**	AMBER, CCPNMR, CYANA, PSVS, TALOS
**Spectrometer**	BRUKER (850 MHZ)

**Protein number**	5
**PDB code**	2LX7, doi:10.2210/pdb2LX7/pdb
**BMRB code**	18662, doi:10.13018/BMR18662
**UniProt code**	O60861, GAS7_HUMAN
**PDB Header**	PROTEIN BINDING
**Protein name**	SH3 DOMAIN OF GROWTH ARREST-SPECIFIC PROTEIN 7 (GAS7) (FRAGMENT 1-60) FROM HOMO SAPIENS
**Deposition date**	15.08.2012
**PDB title**	SOLUTION NMR STRUCTURE OF SH3 DOMAIN OF GROWTH ARREST-SPECIFIC PROTEIN 7 (GAS7) (FRAGMENT 1-60) FROM HOMO SAPIENS, NORTHEAST STRUCTURAL GENOMICS CONSORTIUM (NESG) TARGET HR8574A
**PDB authors**	Y.YANG, T.A.RAMELOT, L.DAN, E.KOHAN, H.JANJUA, R.XIAO, T.ACTON, J.K.EVERETT, G.T.MONTELIONE, M.A.KENNEDY, NORTHEAST STRUCTURAL GENOMICS CONSORTIUM (NESG)
**Last author**	KENNEDY
**Reference**	
**Reference authors**	
**Reference title**	
**Software listed**	AUTOASSIGN, AUTOSTRUCTURE, CNS, CYANA, NMRPIPE, PINE, PSVS, SPARKY, TALOS+, TOPSPIN, VNMRJ
**Spectrometer**	BRUKER, VARIAN (850 MHZ, 600 MHZ)

**Protein number**	6
**PDB code**	2MA6, doi:10.2210/pdb2MA6/pdb
**BMRB code**	19329, doi:10.13018/BMR19329
**UniProt code**	Q5XPI4, RN123_HUMAN
**PDB Header**	LIGASE
**Protein name**	RING FINGER DOMAIN FROM KIP1 UBIQUITINATION-PROMOTING E3 COMPLEX PROTEIN 1 (KPC1/RNF123) FROM HOMO SAPIENS
**Deposition date**	28.06.2013
**PDB title**	SOLUTION NMR STRUCTURE OF THE RING FINGER DOMAIN FROM THE KIP1 UBIQUITINATION-PROMOTING E3 COMPLEX PROTEIN 1 (KPC1/RNF123) FROM HOMO SAPIENS, NORTHEAST STRUCTURAL GENOMICS CONSORTIUM (NESG) TARGET HR8700A
**PDB authors**	T.A.RAMELOT, Y.YANG, H.JANJUA, E.KOHAN, H.WANG, R.XIAO, T.B.ACTON, J.K.EVERETT, G.T.MONTELIONE, M.A.KENNEDY, NORTHEAST STRUCTURAL GENOMICS CONSORTIUM (NESG)
**Last author**	KENNEDY
**Reference**	
**Reference authors**	
**Reference title**	
**Software listed**	AUTOSTRUCTURE, CNS, CYANA, FMCGUI, NMRPIPE, PINE, PSVS, SPARKY, TALOS+, TOPSPIN, VNMRJ
**Spectrometer**	BRUKER, VARIAN (850 MHZ, 600 MHZ)

**Protein number**	7
**PDB code**	2JRM, doi:10.2210/pdb2JRM/pdb
**BMRB code**	15339, doi:10.13018/BMR15339
**UniProt code**	Q87PC4, Q87PC4_VIBPA
**PDB Header**	STRUCTURAL GENOMICS, UNKNOWN FUNCTION
**Protein name**	RIBOSOME MODULATION FACTOR VP1593 FROM VIBRIO PARAHAEMOLYTICUS
**Deposition date**	27.06.2007
**PDB title**	SOLUTION NMR STRUCTURE OF RIBOSOME MODULATION FACTOR VP1593 FROM VIBRIO PARAHAEMOLYTICUS. NORTHEAST STRUCTURAL GENOMICS TARGET VPR55
**PDB authors**	Y.TANG, P.ROSSI, G.SWAPNA, H.WANG, M.JIANG, K.CUNNINGHAM, L.OWENS, L.MA, R.XIAO, J.LIU, M.C.BARAN, T.B.ACTON, B.ROST, G.T.MONTELIONE, NORTHEAST STRUCTURAL GENOMICS CONSORTIUM (NESG)
**Last author**	MONTELIONE
**Reference**	
**Reference authors**	
**Reference title**	
**Software listed**	AUTOASSIGN, AUTOSTRUCTURE, CNS, CYANA, NMRPIPE, PDBSTAT, PSVS, SPARKY, TOPSPIN, X-PLOR
**Spectrometer**	BRUKER (800 MHZ, 600 MHZ)

**Protein number**	8
**PDB code**	1YEZ, doi:10.2210/pdb1YEZ/pdb
**BMRB code**	6505, doi:10.13018/BMR6505
**UniProt code**	Q8PX65, Q8PX65_METMA
**PDB Header**	STRUCTURAL GENOMICS, UNKNOWN FUNCTION
**Protein name**	CONSERVED PROTEIN FROM GENE LOCUS MM1357 OF METHANOSARCINA MAZEI
**Deposition date**	29.12.2004
**PDB title**	SOLUTION STRUCTURE OF THE CONSERVED PROTEIN FROM THE GENE LOCUS MM1357 OF METHANOSARCINA MAZEI. NORTHEAST STRUCTURAL GENOMICS TARGET MAR30

**PDB authors**	P.ROSSI, J.M.ARAMINI, G.V.T.SWAPNA, Y.P.HUANG, R.XIAO, C.K.HO, L.C.MA, T.B.ACTON, G.T.MONTELIONE, NORTHEAST STRUCTURAL GENOMICS CONSORTIUM (NESG)
**Last author**	MONTELIONE
**Reference**	
**Reference authors**	
**Reference title**	
**Software listed**	AUTOASSIGN, AUTOSTRUCTURE, NMRPIPE, SPARKY, VNMR, XWINNMR
**Spectrometer**	BRUKER, VARIAN (600 MHZ, 500 MHZ)

**Protein number**	9
**PDB code**	2L9R, doi:10.2210/pdb2L9R/pdb
**BMRB code**	17484, doi:10.13018/BMR17484
**UniProt code**	Q99801, NKX31_HUMAN
**PDB Header**	TRANSCRIPTION
**Protein name**	HOMEOBOX DOMAIN OF HOMEOBOX PROTEIN NKX-3.1 FROM HOMO SAPIENS
**Deposition date**	22.02.2011
**PDB title**	SOLUTION NMR STRUCTURE OF HOMEOBOX DOMAIN OF HOMEOBOX PROTEIN NKX-3.1 FROM HOMO SAPIENS, NORTHEAST STRUCTURAL GENOMICS CONSORTIUM TARGET HR6470A
**PDB authors**	G.LIU, R.XIAO, H.-W.LEE, K.HAMILTON, C.CICCOSANTI, H.B.WANG, T.B.ACTON, J.K.EVERETT, Y.J.HUANG, G.T.MONTELIONE, NORTHEAST STRUCTURAL GENOMICS CONSORTIUM (NESG)
**Last author**	MONTELIONE
**Reference**	
**Reference authors**	
**Reference title**	
**Software listed**	AUTOASSIGN, AUTOSTRUCTURE, CNS, CYANA, NMRPIPE, TALOS+, TOPSPIN, VNMRJ, XEASY
**Spectrometer**	BRUKER, VARIAN (800 MHZ, 600 MHZ)

**Protein number**	10
**PDB code**	2K52, doi:10.2210/pdb2K52/pdb
**BMRB code**	15821, doi:10.13018/BMR15821
**UniProt code**	Q58598, Y1198_METJA
**PDB Header**	STRUCTURAL GENOMICS, UNKNOWN FUNCTION
**Protein name**	UNCHARACTERIZED PROTEIN MJ1198 FROM METHANOCALDOCOCCUS JANNASCHII
**Deposition date**	24.06.2008
**PDB title**	STRUCTURE OF UNCHARACTERIZED PROTEIN MJ1198 FROM METHANOCALDOCOCCUS JANNASCHII. NORTHEAST STRUCTURAL GENOMICS TARGET MJR117B
**PDB authors**	P.ROSSI, M.MAGLAQUI, E.L.FOOTE, K.HAMILTON, C.CICCOSANTI, R.XIAO, R.NAIR, G.SWAPNA, J.K.EVERETT, T.B.ACTON, B.ROST, G.T.MONTELIONE, NORTHEAST STRUCTURAL GENOMICS CONSORTIUM (NESG)
**Last author**	MONTELIONE
**Reference**	
**Reference authors**	
**Reference title**	
**Software listed**	AUTOASSIGN, CNS, CYANA, MOLMOL, MOLPROBITY, NMRPIPE, PROCHECK, PSVS, RPF(AUTOSTRUCTURE), SPARKY, TALOS, TOPSPIN
**Spectrometer**	BRUKER (800 MHZ, 600 MHZ)

**Protein number**	11
**PDB code**	2KRS, doi:10.2210/pdb2KRS/pdb
**BMRB code**	16647, doi:10.13018/BMR16647
**UniProt code**	Q8XMT2, Q8XMT2_CLOPE
**PDB Header**	STRUCTURAL GENOMICS, UNKNOWN FUNCTION
**Protein name**	SH3 DOMAIN FROM CPF_0587 (FRAGMENT 415-479) FROM CLOSTRIDIUM PERFRINGENS
**Deposition date**	22.12.2009
**PDB title**	SOLUTION NMR STRUCTURE OF SH3 DOMAIN FROM CPF_0587 (FRAGMENT 415-479) FROM CLOSTRIDIUM PERFRINGENS. NORTHEAST STRUCTURAL GENOMICS CONSORTIUM (NESG) TARGET CPR74A
**PDB authors**	T.A.RAMELOT, J.R.CORT, M.MAGLAQUI, C.CICCOSANTI, H.JANJUA, R.NAIR, B.ROST, T.B.ACTON, R.XIAO, J.K.EVERETT, G.T.MONTELIONE, M.A.KENNEDY, NORTHEAST STRUCTURAL GENOMICS CONSORTIUM (NESG)
**Last author**	KENNEDY
**Reference**	
**Reference authors**	
**Reference title**	
**Software listed**	AUTOASSIGN, AUTOSTRUCTURE, CNS, NMRPIPE, PDBSTAT, PSVS, SPARKY, TOPSPIN, VNMR, X-PLOR
**Spectrometer**	BRUKER, VARIAN (850 MHZ, 600 MHZ)

**Protein number**	12
**PDB code**	2K53, doi:10.2210/pdb2K53/pdb
**BMRB code**	15822, doi:10.13018/BMR15822
**UniProt code**	A3DK08, A3DK08_CLOTH
**PDB Header**	STRUCTURAL GENOMICS, UNKNOWN FUNCTION
**Protein name**	A3DK08 PROTEIN FROM CLOSTRIDIUM THERMOCELLUM
**Deposition date**	24.06.2008

**PDB title**	NMR SOLUTION STRUCTURE OF A3DK08 PROTEIN FROM CLOSTRIDIUM THERMOCELLUM: NORTHEAST STRUCTURAL GENOMICS CONSORTIUM TARGET CMR9
**PDB authors**	G.V.T.SWAPNA, W.HUANG, M.JIANG, E.L.FOOTE, R.XIAO, R.NAIR, J.EVERETT, T.B.ACTON, B.ROST, G.T.MONTELIONE, NORTHEAST STRUCTURAL GENOMICS CONSORTIUM (NESG)
**Last author**	MONTELIONE
**Reference**	
**Reference authors**	
**Reference title**	
**Software listed**	AUTOASSIGN, AUTOSTRUCTURE, CNS, CYANA
**Spectrometer**	BRUKER, VARIAN (800 MHZ, 600 MHZ)

**Protein number**	13
**PDB code**	2JT1, doi:10.2210/pdb2JT1/pdb
**BMRB code**	15386, doi:10.13018/BMR15386
**UniProt code**	Q04822, Q04822_SALTY
**PDB Header**	TRANSCRIPTION
**Protein name**	PEFI (PLASMID-ENCODED FIMBRIAE REGULATORY) PROTEIN FROM SALMONELLA TYPHIMURIUM
**Deposition date**	17.07.2007
**PDB title**	SOLUTION NMR STRUCTURE OF PEFI (PLASMID-ENCODED FIMBRIAE REGULATORY) PROTEIN FROM SALMONELLA TYPHIMURIUM. NORTHEAST STRUCTURAL GENOMICS TARGET STR82
**PDB authors**	J.M.ARAMINI, P.ROSSI, H.WANG, C.NWOSU, K.CUNNINGHAM, L.-C.MA, R.XIAO, J.LIU, M.C.BARAN, G.V.T.SWAPNA, T.B.ACTON, B.ROST, G.T.MONTELIONE, NORTHEAST STRUCTURAL GENOMICS CONSORTIUM (NESG)
**Last author**	MONTELIONE
**Reference**	PROTEINS 79, 335 (2011), doi:10.1002/PROT.22869
**Reference authors**	J.M.ARAMINI, P.ROSSI, J.R.CORT, L.C.MA, R.XIAO, T.B.ACTON, G.T.MONTELIONE
**Reference title**	SOLUTION NMR STRUCTURE OF THE PLASMID-ENCODED FIMBRIAE REGULATORY PROTEIN PEFI FROM SALMONELLA ENTERICA SEROVAR TYPHIMURIUM
**Software listed**	AUTOASSIGN, AUTOSTRUCTURE, CNS, CYANA, MOLMOL, MOLPROBITY, NMRPIPE, PDBSTAT, PROCHECK, PSVS, SPARKY, TOPSPIN
**Spectrometer**	BRUKER (800 MHZ, 600 MHZ)

**Protein number**	14
**PDB code**	2JVO, doi:10.2210/pdb2JVO/pdb
**BMRB code**	15485, doi:10.13018/BMR15485
**UniProt code**	Q01560, NOP3_YEAST
**PDB Header**	RNA BINDING PROTEIN
**Protein name**	SEGMENTAL ISOTOPE LABELING OF NPL3
**Deposition date**	24.09.2007
**PDB title**	SEGMENTAL ISOTOPE LABELING OF NPL3
**PDB authors**	L.SKRISOVSKA, F.H.-T.ALLAIN
**Last author**	ALLAIN
**Reference**	J.MOL.BIOL. 375, 151 (2008), doi:10.1016/J.JMB.2007.09.030
**Reference authors**	L.SKRISOVSKA, F.H.ALLAIN
**Reference title**	IMPROVED SEGMENTAL ISOTOPE LABELING METHODS FOR THE NMR STUDY OF MULTIDOMAIN OR LARGE PROTEINS: APPLICATION TO THE RRMS OF NPL3P AND HNRNP L
**Software listed**	ATNOS/CANDID, SPARKY, XWINNMR
**Spectrometer**	BRUKER (900 MHZ, 600 MHZ, 500 MHZ)

**Protein number**	15
**PDB code**	2ERR, doi:10.2210/pdb2ERR/pdb
**BMRB code**	6895, doi:10.13018/BMR6895
**UniProt code**	Q9NWB1, A2BP1_HUMAN
**PDB Header**	RNA BINDING PROTEIN
**Protein name**	RNA BINDING DOMAIN OF HUMAN FOX-1 IN COMPLEX WITH UGCAUGU
**Deposition date**	25.10.2005
**PDB title**	NMR STRUCTURE OF THE RNA BINDING DOMAIN OF HUMAN FOX-1 IN COMPLEX WITH UGCAUGU
**PDB authors**	F.H.ALLAIN, S.D.AUWETER
**Last author**	AUWETER
**Reference**	EMBO J. 25, 163 (2006), doi:10.1038/SJ.EMBOJ.7600918
**Reference authors**	S.D.AUWETER, R.FASAN, L.REYMOND, J.G.UNDERWOOD, D.L.BLACK, S.PITSCH, F.H.ALLAIN
**Reference title**	MOLECULAR BASIS OF RNA RECOGNITION BY THE HUMAN ALTERNATIVE SPLICING FACTOR FOX-1
**Software listed**	AMBER, CYANA
**Spectrometer**	BRUKER (900 MHZ, 600 MHZ, 500 MHZ)

**Protein number**	16
**PDB code**	2L1P, doi:10.2210/pdb2L1P/pdb
**BMRB code**	17092, doi:10.13018/BMR17092
**UniProt code**	Q01826, SATB1_HUMAN
**PDB Header**	DNA BINDING PROTEIN
**Protein name**	N-TERMINAL DOMAIN OF DNA-BINDING PROTEIN SATB1 FROM HOMO SAPIENS
**Deposition date**	02.08.2010

**PDB title**	NMR SOLUTION STRUCTURE OF THE N-TERMINAL DOMAIN OF DNA-BINDING PROTEIN SATB1 FROM HOMO SAPIENS: NORTHEAST STRUCTURAL GENOMICS TARGET HR4435B(179-250)
**PDB authors**	G.V.T.SWAPNA, A.F.MONTELIONE, R.SHASTRY, C.CICCOSANTI, H.JANJUA, R.XIAO, T.B.ACTON, J.K.EVERETT, G.T.MONTELIONE, NORTHEAST STRUCTURAL GENOMICS CONSORTIUM (NESG)
**Last author**	MONTELIONE
**Reference**	
**Reference authors**	
**Reference title**	
**Software listed**	AUTOASSIGN, AUTOSTRUCTURE, CNS, CYANA
**Spectrometer**	BRUKER (800 MHZ, 600 MHZ)

**Protein number**	17
**PDB code**	2LN3, doi:10.2210/pdb2LN3/pdb
**BMRB code**	18145, doi:10.13018/BMR18145
**UniProt code**	
**PDB Header**	DE NOVO PROTEIN
**Protein name**	DE NOVO DESIGNED PROTEIN, IF3-LIKE FOLD
**Deposition date**	15.12.2011
**PDB title**	SOLUTION NMR STRUCTURE OF DE NOVO DESIGNED PROTEIN, IF3-LIKE FOLD, NORTHEAST STRUCTURAL GENOMICS CONSORTIUM TARGET OR135 (CASD TARGET)
**PDB authors**	G.LIU, R.KOGA, N.KOGA, R.XIAO, H.LEE, H.JANJUA, E.KOHAN, T.B.ACTON, J.K.EVERETT, D.BAKER, G.T.MONTELIONE, NORTHEAST STRUCTURAL GENOMICS CONSORTIUM (NESG)
**Last author**	MONTELIONE
**Reference**	NATURE 491, 222 (2012), doi:10.1038/NATURE11600
**Reference authors**	N.KOGA, R.TATSUMI-KOGA, G.LIU, R.XIAO, T.B.ACTON, G.T.MONTELIONE, D.BAKER
**Reference title**	PRINCIPLES FOR DESIGNING IDEAL PROTEIN STRUCTURES
**Software listed**	AUTOASSIGN, AUTOSTRUCTURE, CNS, CYANA, NMRPIPE, REDCAT, SPARKY, TALOS+, TOPSPIN, VNMRJ, XEASY
**Spectrometer**	BRUKER, VARIAN (800 MHZ, 600 MHZ)

**Protein number**	18
**PDB code**	2HEQ, doi:10.2210/pdb2HEQ/pdb
**BMRB code**	7175, doi:10.13018/BMR7175
**UniProt code**	O31898, O31898_BACSU
**PDB Header**	STRUCTURAL GENOMICS, UNKNOWN FUNCTION
**Protein name**	BACILLUS SUBTILIS PROTEIN YORP
**Deposition date**	21.06.2006
**PDB title**	NMR STRUCTURE OF BACILLUS SUBTILIS PROTEIN YORP, NORTHEAST STRUCTURAL GENOMICS TARGET SR399
**PDB authors**	T.A.RAMELOT, J.R.CORT, D.WANG, H.JANJUA, K.CUNNINGHAM, L.-C.MA, R.XIAO, J.LIU, M.BARAN, G.V.T.SWAPNA, T.B.ACTON, B.ROST, G.M.MONTELIONE, M.A.KENNEDY, NORTHEAST STRUCTURAL GENOMICS CONSORTIUM (NESG)
**Last author**	KENNEDY
**Reference**	
**Reference authors**	
**Reference title**	
**Software listed**	AUTOSTRUCTURE, CNS, NMRPIPE, SPARKY, VNMR, X-PLOR_NIH
**Spectrometer**	VARIAN (750 MHZ, 600 MHZ)

**Protein number**	19
**PDB code**	2KK8, doi:10.2210/pdb2KK8/pdb
**BMRB code**	16355, doi:10.13018/BMR16355
**UniProt code**	Q9M0W8, Q9M0W8_ARATH
**PDB Header**	STRUCTURAL GENOMICS, UNKNOWN FUNCTION
**Protein name**	A PUTATIVE UNCHARACTERIZED PROTEIN OBTAINED FROM ARABIDOPSIS THALIANA
**Deposition date**	16.06.2009
**PDB title**	NMR SOLUTION STRUCTURE OF A PUTATIVE UNCHARACTERIZED PROTEIN OBTAINED FROM ARABIDOPSIS THALIANA: NORTHEAST STRUCTURAL GENOMICS CONSORTIUM TARGET AR3449A
**PDB authors**	R.MANI, S.V.T.GURLA, R.SHASTRY, C.CICCOSANTI, E.FOOTE, M.JIANG, R.XIAO, R.NAIR, J.EVERETT, Y.HUANG, T.ACTON, B.ROST, G.T.MONTELIONE, NORTHEAST STRUCTURAL GENOMICS CONSORTIUM (NESG)
**Last author**	MONTELIONE
**Reference**	
**Reference authors**	
**Reference title**	
**Software listed**	AUTOASSIGN, AUTOSTRUCTURE, CNS, CYANA
**Spectrometer**	BRUKER, VARIAN (800 MHZ, 600 MHZ, 500 MHZ)

**Protein number**	20
**PDB code**	2KD0, doi:10.2210/pdb2KD0/pdb
**BMRB code**	16101, doi:10.13018/BMR16101
**UniProt code**	P0C895, Y2010_ARATH
**PDB Header**	SIGNALING PROTEIN
**Protein name**	O64736 PROTEIN FROM ARABIDOPSIS THALIANA
**Deposition date**	31.12.2008

**PDB title**	NMR SOLUTION STRUCTURE OF O64736 PROTEIN FROM ARABIDOPSIS THALIANA. NORTHEAST STRUCTURAL GENOMICS CONSORTIUM MEGA TARGET AR3445A
**PDB authors**	G.V.T.SWAPNA, R.SHASTRY, E.FOOTE, C.CICCOSANTI, M.JIANG, R.XIAO, R.NAIR, J.EVERETT, Y.HUANG, T.B.ACTON, B.ROST, G.T.MONTELIONE, NORTHEAST STRUCTURAL GENOMICS CONSORTIUM (NESG)
**Last author**	MONTELIONE
**Reference**	
**Reference authors**	
**Reference title**	
**Software listed**	AUTOASSIGN, AUTOSTRUCTURE, CNS, CYANA, NMRPIPE, SPARKY
**Spectrometer**	BRUKER (800 MHZ, 600 MHZ)

**Protein number**	21
**PDB code**	2LML, doi:10.2210/pdb2LML/pdb
**BMRB code**	16860, doi:10.13018/BMR16860
**UniProt code**	Q39T60, Q39T60_GEOMG
**PDB Header**	TRANSPORT PROTEIN
**Protein name**	HOLO ACYL CARRIER PROTEIN FROM GEOBACTER METALLIREDUCENS REFINED WITH NH RDCS
**Deposition date**	05.12.2011
**PDB title**	SOLUTION NMR STRUCTURE OF HOLO ACYL CARRIER PROTEIN FROM GEOBACTER METALLIREDUCENS REFINED WITH NH RDCS, NORTHEAST STRUCTURAL GENOMICS CONSORTIUM TARGET GMR141
**PDB authors**	T.A.RAMELOT, M.J.SMOLA, H.LEE, L.ZHAO, C.CICCOSANTI, E.L.FOOTE, K.HAMILTON, R.NAIR, B.ROST, G.SWAPNA, T.B.ACTON, R.XIAO, J.K.EVERETT, J.H.PRESTEGARD, G.T.MONTELIONE, M.A.KENNEDY, NORTHEAST STRUCTURAL GENOMICS CONSORTIUM (NESG)
**Last author**	KENNEDY
**Reference**	BIOCHEMISTRY 50, 1442 (2011), doi:10.1021/BI101932S
**Reference authors**	T.A.RAMELOT, M.J.SMOLA, H.W.LEE, C.CICCOSANTI, K.HAMILTON, T.B.ACTON, R.XIAO, J.K.EVERETT, J.H.PRESTEGARD, G.T.MONTELIONE, M.A.KENNEDY
**Reference title**	SOLUTION STRUCTURE OF 4'-PHOSPHOPANTETHEINE - GMACP3 FROM GEOBACTER METALLIREDUCENS: A SPECIALIZED ACYL CARRIER PROTEIN WITH ATYPICAL STRUCTURAL FEATURES AND A PUTATIVE ROLE IN LIPOPOLYSACCHARIDE BIOSYNTHESIS
**Software listed**	AUTOASSIGN, AUTOSTRUCTURE, CNS, CYANA, FMCGUI, NMRPIPE, PDBSTAT, PINE_SERVER, PSVS, SPARKY, TOPSPIN, VNMR, X-PLOR_NIH
**Spectrometer**	BRUKER, VARIAN (850 MHZ, 600 MHZ)

**Protein number**	22
**PDB code**	2K3D, doi:10.2210/pdb2K3D/pdb
**BMRB code**	15750, doi:10.13018/BMR15750
**UniProt code**	Q92EX4, Q92EX4_LISIN
**PDB Header**	STRUCTURAL GENOMICS, UNKNOWN FUNCTION
**Protein name**	FOLDED 79 RESIDUE FRAGMENT OF LIN0334 FROM LISTERIA INNOCUA
**Deposition date**	02.05.2008
**PDB title**	SOLUTION NMR STRUCTURE OF THE FOLDED 79 RESIDUE FRAGMENT OF LIN0334 FROM LISTERIA INNOCUA. NORTHEAST STRUCTURAL GENOMICS CONSORTIUM TARGET LKR15
**PDB authors**	T.A.RAMELOT, L.ZHAO, M.JIANG, E.L.FOOTE, R.XIAO, J.LIU, M.C.BARAN, G.V.T.SWAPNA, T.B.ACTON, B.ROST, G.T.MONTELIONE, M.A.KENNEDY, NORTHEAST STRUCTURAL GENOMICS CONSORTIUM (NESG)
**Last author**	KENNEDY
**Reference**	
**Reference authors**	
**Reference title**	
**Software listed**	AUTOASSIGN, AUTOSTRUCTURE, NMRPIPE, PSVS, SPARKY, TOPSPIN, VNMR, X-PLOR
**Spectrometer**	BRUKER, VARIAN (850 MHZ, 600 MHZ)

**Protein number**	23
**PDB code**	2LK2, doi:10.2210/pdb2LK2/pdb
**BMRB code**	17971, doi:10.13018/BMR17971
**UniProt code**	Q15583, TGIF1_HUMAN
**PDB Header**	TRANSCRIPTION
**Protein name**	HOMEOBOX DOMAIN (171-248) OF HUMAN HOMEOBOX PROTEIN TGIF1
**Deposition date**	30.09.2011
**PDB title**	SOLUTION NMR STRUCTURE OF HOMEOBOX DOMAIN (171-248) OF HUMAN HOMEOBOX PROTEIN TGIF1, NORTHEAST STRUCTURAL GENOMICS CONSORTIUM TARGET HR4411B
**PDB authors**	Y.YANG, T.A.RAMELOT, J.R.CORT, R.SHASTRY, C.CICCOSANTI, K.HAMILTON, T.B.ACTON, R.XIAO, J.K.EVERETT, G.T.MONTELIONE, M.A.KENNEDY, NORTHEAST STRUCTURAL GENOMICS CONSORTIUM (NESG)
**Last author**	KENNEDY
**Reference**	
**Reference authors**	
**Reference title**	
**Software listed**	ASDP, AUTOASSIGN, CNS, CYANA, NMRPIPE, PDBSTAT, PINE_SERVER, PSVS, SPARKY, TOPSPIN, VNMR, X-PLOR_NIH
**Spectrometer**	BRUKER, VARIAN (850 MHZ, 600 MHZ)

**Protein number**	24

**PDB code**	(MH04)
**BMRB code**	
**PDB Header**	
**Protein name**	
**Deposition date**	
**PDB title**	
**PDB authors**	
**Last author**	(IWAI)
**Reference**	
**Reference authors**	
**Reference title**	
**Software listed**	
**Spectrometer**	(BRUKER (800 MHZ, 600 MHZ))

**Protein number**	25
**PDB code**	1PQX, doi:10.2210/pdb1PQX/pdb
**BMRB code**	5844, doi:10.13018/BMR5844
**UniProt code**	Q99U58, Q99U58_STAAM
**PDB Header**	STRUCTURAL GENOMICS, UNKNOWN FUNCTION
**Protein name**	STAPHYLOCOCCUS AUREUS PROTEIN SAV1430
**Deposition date**	19.06.2003
**PDB title**	SOLUTION NMR STRUCTURE OF STAPHYLOCOCCUS AUREUS PROTEIN SAV1430. NORTHEAST STRUCTURAL GENOMICS CONSORTIUM TARGET ZR18
**PDB authors**	M.C.BARAN, J.M.ARAMINI, R.XIAO, Y.J.HUANG, T.B.ACTON, L.SHIH, G.T.MONTELIONE, NORTHEAST STRUCTURAL GENOMICS CONSORTIUM (NESG)
**Last author**	MONTELIONE
**Reference**	
**Reference authors**	
**Reference title**	
**Software listed**	AUTOASSIGN, AUTOPROC, AUTOSTRUCTURE, NMRPIPE, SPARKY, VNMR, X-PLOR
**Spectrometer**	VARIAN (600 MHZ, 500 MHZ)

**Protein number**	26
**PDB code**	2L33, doi:10.2210/pdb2L33/pdb
**BMRB code**	17169, doi:10.13018/BMR17169
**UniProt code**	Q12906, ILF3_HUMAN
**PDB Header**	TRANSCRIPTION REGULATOR
**Protein name**	DRBM 2 DOMAIN OF INTERLEUKIN ENHANCER- BINDING FACTOR 3 FROM HOMO SAPIENS
**Deposition date**	03.09.2010
**PDB title**	SOLUTION NMR STRUCTURE OF DRBM 2 DOMAIN OF INTERLEUKIN ENHANCER- BINDING FACTOR 3 FROM HOMO SAPIENS, NORTHEAST STRUCTURAL GENOMICS CONSORTIUM TARGET HR4527E
**PDB authors**	G.LIU, H.JANJUA, R.XIAO, T.B.ACTON, A.CICCOSANTI, R.B.SHASTRY, J.EVERETT, G.T.MONTELIONE, NORTHEAST STRUCTURAL GENOMICS CONSORTIUM, NORTHEAST STRUCTURAL GENOMICS CONSORTIUM (NESG)
**Last author**	MONTELIONE
**Reference**	
**Reference authors**	
**Reference title**	
**Software listed**	AUTOASSIGN, AUTOSTRUCTURE, CNS, CYANA, NMRPIPE, TALOS+, TOPSPIN, VNMRJ, XEASY
**Spectrometer**	BRUKER, VARIAN (800 MHZ, 600 MHZ)

**Protein number**	27
**PDB code**	2KZV, doi:10.2210/pdb2KZV/pdb
**BMRB code**	17020, doi:10.13018/BMR17020
**UniProt code**	Q7P141, Q7P141_CHRVO
**PDB Header**	STRUCTURAL GENOMICS, UNKNOWN FUNCTION
**Protein name**	CV_0373(175-257) PROTEIN FROM CHROMOBACTERIUM VIOLACEUM
**Deposition date**	25.06.2010
**PDB title**	SOLUTION NMR STRUCTURE OF CV_0373(175-257) PROTEIN FROM CHROMOBACTERIUM VIOLACEUM, NORTHEAST STRUCTURAL GENOMICS CONSORTIUM TARGET CVR118A
**PDB authors**	Y.YANG, T.A.RAMELOT, D.WANG, C.CICCOSANTI, L.MAO, H.JANJUA, T.B.ACTON, R.XIAO, J.K.EVERETT, G.T.MONTELIONE, M.A.KENNEDY, NORTHEAST STRUCTURAL GENOMICS CONSORTIUM (NESG)
**Last author**	KENNEDY
**Reference**	
**Reference authors**	
**Reference title**	
**Software listed**	AUTOASSIGN, AUTOSTRUCTURE, CNS, CYANA, NMRPIPE, PDBSTAT, PINE, PSVS, SPARKY, TOPSPIN, VNMR, X-PLOR
**Spectrometer**	BRUKER, VARIAN (850 MHZ, 600 MHZ)

**Protein number**	28
**PDB code**	2KCT, doi:10.2210/pdb2KCT/pdb
**BMRB code**	16096, doi:10.13018/BMR16096
**UniProt code**	Q72D78, Q72D78_DESVH

**PDB Header**	CHAPERONE
**Protein name**	OB-FOLD DOMAIN OF HEME CHAPERONE CCME FROM DESULFOVIBRIO VULGARIS
**Deposition date**	29.12.2008
**PDB title**	SOLUTION NMR STRUCTURE OF THE OB-FOLD DOMAIN OF HEME CHAPERONE CCME FROM DESULFOVIBRIO VULGARIS. NORTHEAST STRUCTURAL GENOMICS TARGET DVR115G
**PDB authors**	J.M.ARAMINI, P.ROSSI, H.LEE, A.LEMAK, H.WANG, E.L.FOOTE, M.JIANG, R.XIAO, R.NAIR, G.V.T.SWAPNA, T.B.ACTON, B.ROST, J.K.EVERETT, G.T.MONTELIONE, NORTHEAST STRUCTURAL GENOMICS CONSORTIUM (NESG)
**Last author**	MONTELIONE
**Reference**	
**Reference authors**	
**Reference title**	
**Software listed**	AUTOASSIGN, AUTOSTRUCTURE, CNS, CYANA, NMRPIPE, PALES, PDBSTAT, PINE, PSVS, SPARKY, TOPSPIN, VNMRJ
**Spectrometer**	BRUKER, VARIAN (800 MHZ, 600 MHZ)

**Protein number**	29
**PDB code**	2MDR, doi:10.2210/pdb2MDR/pdb
**BMRB code**	19502, doi:10.13018/BMR19502
**UniProt code**	P55265, DSRAD_HUMAN
**PDB Header**	HYDROLASE
**Protein name**	THIRD DOUBLE-STRANDED RNA-BINDING DOMAIN (DSRBD3) OF HUMAN ADENOSINE-DEAMINASE ADAR1
**Deposition date**	17.09.2013
**PDB title**	SOLUTION STRUCTURE OF THE THIRD DOUBLE-STRANDED RNA-BINDING DOMAIN (DSRBD3) OF HUMAN ADENOSINE-DEAMINASE ADAR1
**PDB authors**	P.BARRAUD, S.BANERJEE, W.I.MOHAMED, M.F.JANTSCH, F.H.ALLAIN
**Last author**	ALLAIN
**Reference**	PROC.NATL.ACAD.SCI.USA 111, E1852 (2014), doi:10.1073/PNAS.1323698111
**Reference authors**	P.BARRAUD, S.BANERJEE, W.I.MOHAMED, M.F.JANTSCH, F.H.ALLAIN
**Reference title**	A BIMODULAR NUCLEAR LOCALIZATION SIGNAL ASSEMBLED VIA AN EXTENDED DOUBLE-STRANDED RNA-BINDING DOMAIN ACTS AS AN RNA-SENSING SIGNAL FOR TRANSPORTIN 1
**Software listed**	ATNOS, CING, CNS, CYANA, PROCHECKNMR, SPARKY, TOPSPIN
**Spectrometer**	BRUKER (900 MHZ, 750 MHZ, 700 MHZ, 600 MHZ, 500 MHZ)

**Protein number**	30
**PDB code**	2FB7, doi:10.2210/pdb2FB7/pdb
**BMRB code**	7084, doi:10.13018/BMR7084
**UniProt code**	Q7SXR4, Q7SXR4_BRARE
**PDB Header**	STRUCTURAL GENOMICS, UNKNOWN FUNCTION
**Protein name**	PROTEIN FROM ZEBRA FISH DR.13312
**Deposition date**	08.12.2005
**PDB title**	NMR SOLUTION STRUCTURE OF PROTEIN FROM ZEBRA FISH DR.13312
**PDB authors**	R.C.TYLER, J.SONG, J.L.MARKLEY, CENTER FOR EUKARYOTIC STRUCTURAL GENOMICS (CESG)
**Last author**	MARKLEY
**Reference**	
**Reference authors**	
**Reference title**	
**Software listed**	ARIA, CNS, NMRPIPE, NMRVIEW
**Spectrometer**	VARIAN (600 MHZ)

**Protein number**	31
**PDB code**	2MB0, doi:10.2210/pdb2MB0/pdb
**BMRB code**	19382, doi:10.13018/BMR19382
**UniProt code**	P38159, RBMX_HUMAN
**PDB Header**	SPLICING/RNA
**Protein name**	HNRNP G RRM IN COMPLEX WITH RNA 5'-AUCAAA-3'
**Deposition date**	22.07.2013
**PDB title**	SOLUTION STRUCTURE OF HNRNP G RRM IN COMPLEX WITH THE RNA 5'-AUCAAA-3'
**PDB authors**	A.MOURSY, F.H.-T.ALLAIN, A.CLERY
**Last author**	CLERY
**Reference**	NUCLEIC ACIDS RES. 42, 6659 (2014), doi:10.1093/NAR/GKU244
**Reference authors**	A.MOURSY, F.H.ALLAIN, A.CLERY
**Reference title**	CHARACTERIZATION OF THE RNA RECOGNITION MODE OF HNRNP G EXTENDS ITS ROLE IN SMN2 SPLICING REGULATION
**Software listed**	AMBER
**Spectrometer**	BRUKER (900 MHZ, 700 MHZ, 600 MHZ, 500 MHZ)

**Protein number**	32
**PDB code**	2L05, doi:10.2210/pdb2L05/pdb
**BMRB code**	17030, doi:10.13018/BMR17030
**UniProt code**	P15056, BRAF_HUMAN
**PDB Header**	TRANSFERASE
**Protein name**	RAS-BINDING DOMAIN OF SERINE/THREONINE- PROTEIN KINASE B-RAF FROM HOMO SAPIENS
**Deposition date**	30.06.2010

**PDB title**	SOLUTION NMR STRUCTURE OF THE RAS-BINDING DOMAIN OF SERINE/THREONINE- PROTEIN KINASE B-RAF FROM HOMO SAPIENS, NORTHEAST STRUCTURAL GENOMICS CONSORTIUM TARGET HR4694F
**PDB authors**	J.M.ARAMINI, H.JANJUA, C.CICCOSANTI, R.SHASTRY, Y.J.HUANG, T.B.ACTON, R.XIAO, J.K.EVERETT, G.T.MONTELIONE, NORTHEAST STRUCTURAL GENOMICS CONSORTIUM (NESG)
**Last author**	MONTELIONE
**Reference**	
**Reference authors**	
**Reference title**	
**Software listed**	AUTOSTRUCTURE, CNS, CYANA, MOLPROBITY, NMRPIPE, PDBSTAT, PINE, PSVS, SPARKY, TALOS+, TOPSPIN
**Spectrometer**	BRUKER (800 MHZ, 600 MHZ)

**Protein number**	33
**PDB code**	2KJR, doi:10.2210/pdb2KJR/pdb
**BMRB code**	16338, doi:10.13018/BMR16338
**UniProt code**	A1ZBM2, A1ZBM2_DROME
**PDB Header**	CHAPERONE
**Protein name**	N-TERMINAL UBIQUITIN-LIKE DOMAIN FROM TUBULIN-BINDING COFACTOR B, CG11242, FROM DROSOPHILA MELANOGASTER
**Deposition date**	08.06.2009
**PDB title**	SOLUTION NMR STRUCTURE OF THE N-TERMINAL UBIQUITIN-LIKE DOMAIN FROM TUBULIN-BINDING COFACTOR B, CG11242, FROM DROSOPHILA MELANOGASTER. NORTHEAST STRUCTURAL GENOMICS CONSORTIUM TARGET FR629A (RESIDUES 8- 92)
**PDB authors**	T.A.RAMELOT, J.R.CORT, R.SHASTRY, C.CICCOSANTI, M.JIANG, R.NAIR, B.ROST, G.SWAPNA, T.B.ACTON, R.XIAO, J.K.EVERETT, G.T.MONTELIONE, M.A.KENNEDY, NORTHEAST STRUCTURAL GENOMICS CONSORTIUM (NESG)
**Last author**	KENNEDY
**Reference**	
**Reference authors**	
**Reference title**	
**Software listed**	AUTOASSIGN, AUTOSTRUCTURE, NMRPIPE, PDBSTAT, PSVS, SPARKY, TOPSPIN, VNMR, X-PLOR
**Spectrometer**	BRUKER, VARIAN (850 MHZ, 500 MHZ)

**Protein number**	34
**PDB code**	2M5O, doi:10.2210/pdb2M5O/pdb
**BMRB code**	19068, doi:10.13018/BMR19068
**UniProt code**	Q9UMS0, NFU1_HUMAN
**PDB Header**	BIOSYNTHETIC PROTEIN
**Protein name**	SOLUTION NMR STRUCTURE CTD DOMAIN OF NFU1 IRON-SULFUR CLUSTER SCAFFOLD HOMOLOG FROM HOMO SAPIENS
**Deposition date**	01.03.2013
**PDB title**	SOLUTION NMR STRUCTURE CTD DOMAIN OF NFU1 IRON-SULFUR CLUSTER SCAFFOLD HOMOLOG FROM HOMO SAPIENS, NORTHEAST STRUCTURAL GENOMICS CONSORTIUM (NESG) TARGET HR2876C
**PDB authors**	G.LIU, R.XIAO, H.JANJUA, K.HAMILTON, R.SHASTRY, E.KOHAN, T.B.ACTON, J.K.EVERETT, K.PEDERSON, Y.J.HUANG, G.T.MONTELIONE, NORTHEAST STRUCTURAL GENOMICS CONSORTIUM (NESG), MITOCHONDRIAL PROTEIN PARTNERSHIP (MPP)
**Last author**	MONTELIONE
**Reference**	
**Reference authors**	
**Reference title**	
**Software listed**	AUTOASSIGN, AUTOSTRUCTURE, CNS, CYANA, NMRPIPE, PSVS, REDCAT, SPARKY, TALOS+, TOPSPIN, VNMRJ, XEASY
**Spectrometer**	BRUKER, VARIAN (800 MHZ, 600 MHZ)

**Protein number**	35
**PDB code**	(MDM2)
**BMRB code**	
**PDB Header**	
**Protein name**	(MDM2)
**Deposition date**	
**PDB title**	
**PDB authors**	
**Last author**	(RIEK)
**Reference**	
**Reference authors**	
**Reference title**	
**Software listed**	
**Spectrometer**	(BRUKER (700 MHZ))

**Protein number**	36
**PDB code**	2LNA, doi:10.2210/pdb2LNA/pdb
**BMRB code**	18156, doi:10.13018/BMR18156
**UniProt code**	Q9Y4W6, AFG32_HUMAN
**PDB Header**	HYDROLASE

**Protein name**	MITOCHONDRIAL INNER MEMBRANE DOMAIN (RESIDUES 164-251), FTSH_EXT, FROM PARAPLEGIN-LIKE PROTEIN AFG3L2 FROM HOMO SAPIENS
**Deposition date**	20.12.2011
**PDB title**	SOLUTION NMR STRUCTURE OF THE MITOCHONDRIAL INNER MEMBRANE DOMAIN (RESIDUES 164-251), FTSH_EXT, FROM THE PARAPLEGIN-LIKE PROTEIN AFG3L2 FROM HOMO SAPIENS, NORTHEAST STRUCTURAL GENOMICS CONSORTIUM TARGET HR6741A
**PDB authors**	T.A.RAMELOT, Y.YANG, H.LEE, H.JANUA, E.KOHAN, R.SHASTRY, T.B.ACTON, R.XIAO, J.K.EVERETT, J.H.PRESTEGARD, G.T.MONTELIONE, M.A.KENNEDY, NORTHEAST STRUCTURAL GENOMICS CONSORTIUM (NESG), MITOCHONDRIAL PROTEIN PARTNERSHIP (MPP)
**Last author**	KENNEDY
**Reference**	FEBS LETT. 587, 3522 (2013), doi:10.1016/J.FEBSLET.2013.09.009
**Reference authors**	T.A.RAMELOT, Y.YANG, I.D.SAHU, H.W.LEE, R.XIAO, G.A.LORIGAN, G.T.MONTELIONE, M.A.KENNEDY
**Reference title**	NMR STRUCTURE AND MD SIMULATIONS OF THE AAA PROTEASE INTERMEMBRANE SPACE DOMAIN INDICATES PERIPHERAL MEMBRANE LOCALIZATION WITHIN THE HEXAOLIGOMER
**Software listed**	AUTOSTRUCTURE, CNS, CYANA, NMRPIPE, PALES, PINE, PSVS, SPARKY, TALOS+, TOPSPIN, VNMRJ, X-PLOR_NIH
**Spectrometer**	BRUKER, VARIAN (850 MHZ, 600 MHZ)

**Protein number**	37
**PDB code**	2LA6, doi:10.2210/pdb2LA6/pdb
**BMRB code**	17508, doi:10.13018/BMR17508
**UniProt code**	P35637, FUS_HUMAN
**PDB Header**	RNA BINDING PROTEIN
**Protein name**	RRM DOMAIN OF RNA-BINDING PROTEIN FUS FROM HOMO SAPIENS
**Deposition date**	04.03.2011
**PDB title**	SOLUTION NMR STRUCTURE OF RRM DOMAIN OF RNA-BINDING PROTEIN FUS FROM HOMO SAPIENS, NORTHEAST STRUCTURAL GENOMICS CONSORTIUM TARGET HR6430A
**PDB authors**	G.LIU, R.XIAO, H.JANJUA, C.CICCOSANTI, H.WANG, H.LEE, T.B.ACTON, J.K.EVERETT, Y.J.HUANG, G.T.MONTELIONE, NORTHEAST STRUCTURAL GENOMICS CONSORTIUM (NESG)
**Last author**	MONTELIONE
**Reference**	
**Reference authors**	
**Reference title**	
**Software listed**	AUTOASSIGN, AUTOSTRUCTURE, CNS, CYANA, NMRPIPE, SPARKY, TALOS+, TOPSPIN, VNMRJ, XEASY
**Spectrometer**	BRUKER, VARIAN (800 MHZ, 600 MHZ)

**Protein number**	38
**PDB code**	6FIP, doi:10.2210/pdb6FIP/pdb
**BMRB code**	34235, doi:10.13018/BMR34235
**UniProt code**	Q51368, TONB_PSEAE
**PDB Header**	TRANSPORT PROTEIN
**Protein name**	PSEUDOMONAS AERUGINOSA TONB CTD
**Deposition date**	19.01.2018
**PDB title**	SOLUTION NMR STRUCTURE OF PSEUDOMONAS AERUGINOSA TONB CTD
**PDB authors**	J.S.OEEMIG, O.H.SAMULI OLLILA, H.A.HEIKKINEN, H.IWAI
**Last author**	IWAI
**Reference**	PEERJ 6, E5412 (2018), doi:10.7717/PEERJ.5412
**Reference authors**	J.S.OEEMIG, O.H.S.OLLILA, H.IWAI
**Reference title**	NMR STRUCTURE OF THE C-TERMINAL DOMAIN OF TONB PROTEIN FROMPSEUDOMONAS AERUGINOSA
**Software listed**	AMBER, CCPNMR, CYANA, NMRPIPE
**Spectrometer**	VARIAN (800 MHZ)

**Protein number**	39
**PDB code**	2LEA, doi:10.2210/pdb2LEA/pdb
**BMRB code**	17705, doi:10.13018/BMR17705
**UniProt code**	Q01130, SRSF2_HUMAN
**PDB Header**	RNA BINDING PROTEIN
**Protein name**	HUMAN SRSF2 (SC35) RRM
**Deposition date**	15.06.2011
**PDB title**	SOLUTION STRUCTURE OF HUMAN SRSF2 (SC35) RRM
**PDB authors**	G.M.DAUBNER, A.CLERY, S.JAYNE, J.STEVENIN, F.H.-T.ALLAIN
**Last author**	ALLAIN
**Reference**	EMBO J. 31, 162 (2012), doi:10.1038/EMBOJ.2011.367
**Reference authors**	G.M.DAUBNER, A.CLERY, S.JAYNE, J.STEVENIN, F.H.ALLAIN
**Reference title**	A SYN-ANTI CONFORMATIONAL DIFFERENCE ALLOWS SRSF2 TO RECOGNIZE GUANINES AND CYTOSINES EQUALLY WELL
**Software listed**	AMBER, CYANA, SPARKY
**Spectrometer**	BRUKER (900 MHZ, 700 MHZ, 600 MHZ, 500 MHZ)

**Protein number**	40
**PDB code**	2LL8, doi:10.2210/pdb2LL8/pdb
**BMRB code**	18032, doi:10.13018/BMR18032
**UniProt code**	Q6N882, Q6N882_RHOPA

**PDB Header**	TRANSFERASE
**Protein name**	SPECIALIZED HOLO-ACYL CARRIER PROTEIN RPA2022 FROM RHODOPSEUDOMONAS PALUSTRIS REFINED WITH NH RDCS
**Deposition date**	31.10.2011
**PDB title**	SOLUTION NMR STRUCTURE OF THE SPECIALIZED HOLO-ACYL CARRIER PROTEIN RPA2022 FROM RHODOPSEUDOMONAS PALUSTRIS REFINED WITH NH RDCS, NORTHEAST STRUCTURAL GENOMICS CONSORTIUM TARGET RPR324
**PDB authors**	T.A.RAMELOT, S.NI, P.ROSSI, Y.YANG, H.WANG, C.CICCOSANTI, M.MAGLAQUI, H.JANJUA, R.NAIR, B.ROSET, T.B.ACTON, R.XIAO, J.K.EVERETT, J.H.PRESTEGARD, G.T.MONTELIONE, M.A.KENNEDY, NORTHEAST STRUCTURAL GENOMICS CONSORTIUM (NESG)
**Last author**	KENNEDY
**Reference**	BIOCHEMISTRY 51, 7239 (2012), doi:10.1021/BI300546B
**Reference authors**	T.A.RAMELOT, P.ROSSI, F.FOROUHAR, H.W.LEE, Y.YANG, S.NI, S.UNSER, S.LEW, J.SEETHARAMAN, R.XIAO, T.B.ACTON, J.K.EVERETT, J.H.PRESTEGARD, J.F.HUNT, G.T.MONTELIONE, M.A.KENNEDY
**Reference title**	STRUCTURE OF A SPECIALIZED ACYL CARRIER PROTEIN ESSENTIAL FOR LIPID A BIOSYNTHESIS WITH VERY LONG-CHAIN FATTY ACIDS IN OPEN AND CLOSED CONFORMATIONS
**Software listed**	AUTOASSIGN, AUTOSTRUCTURE, CNS, CYANA, FMCGUI, NMRPIPE, PDBSTAT, PINE_SERVER, PSVS, SPARKY, TOPSPIN, VNMR, X-PLOR_NIH
**Spectrometer**	BRUKER, VARIAN (850 MHZ, 600 MHZ)

**Protein number**	41
**PDB code**	2KPN, doi:10.2210/pdb2KPN/pdb
**BMRB code**	16561, doi:10.13018/BMR16561
**UniProt code**	Q81D73, Q81D73_BACCR
**PDB Header**	HYDROLASE
**Protein name**	A BACTERIAL IG-LIKE (BIG_3) DOMAIN FROM BACILLUS CEREUS
**Deposition date**	16.10.2009
**PDB title**	SOLUTION NMR STRUCTURE OF A BACTERIAL IG-LIKE (BIG_3) DOMAIN FROM BACILLUS CEREUS. NORTHEAST STRUCTURAL GENOMICS CONSORTIUM TARGET BCR147A
**PDB authors**	J.M.ARAMINI, D.WANG, C.T.CICCOSANTI, H.JANJUA, B.ROST, T.B.ACTON, R.XIAO, G.V.T.SWAPNA, J.K.EVERETT, G.T.MONTELIONE, NORTHEAST STRUCTURAL GENOMICS CONSORTIUM (NESG)
**Last author**	MONTELIONE
**Reference**	
**Reference authors**	
**Reference title**	
**Software listed**	AUTOSTRUCTURE, CNS, CYANA, MOLPROBITY, NMRPIPE, PDBSTAT, PINE, PSVS, SPARKY, TALOS, TOPSPIN
**Spectrometer**	BRUKER (800 MHZ, 600 MHZ)

**Protein number**	42
**PDB code**	2K0M, doi:10.2210/pdb2K0M/pdb
**BMRB code**	15652, doi:10.13018/BMR15652
**UniProt code**	Q2RW81, Q2RW81_RHORT
**PDB Header**	STRUCTURAL GENOMICS, UNKNOWN FUNCTION
**Protein name**	UNCHARACTERIZED PROTEIN FROM RHODOSPIRILLUM RUBRUM GENE LOCUS RRU_A0810
**Deposition date**	04.02.2008
**PDB title**	SOLUTION NMR STRUCTURE OF THE UNCHARACTERIZED PROTEIN FROM RHODOSPIRILLUM RUBRUM GENE LOCUS RRU_A0810. NORTHEAST STRUCTURAL GENOMICS TARGET RRR43
**PDB authors**	P.ROSSI, H.WANG, M.JIANG, E.L.FOOTE, R.XIAO, J.LIU, G.SWAPNA, T.B.ACTON, M.C.BARAN, B.ROST, G.T.MONTELIONE, NORTHEAST STRUCTURAL GENOMICS CONSORTIUM (NESG)
**Last author**	MONTELIONE
**Reference**	
**Reference authors**	
**Reference title**	
**Software listed**	AUTOASSIGN, CNS, CYANA, MOLMOL, MOLPROBITY, NMRPIPE, PROCHECKNMR, PSVS, SPARKY, TALOS, TOPSPIN
**Spectrometer**	BRUKER (800 MHZ)

**Protein number**	43
**PDB code**	2K5V, doi:10.2210/pdb2K5V/pdb
**BMRB code**	15849, doi:10.13018/BMR15849
**UniProt code**	Q6LYF9, Q6LYF9_METMP
**PDB Header**	DNA BINDING PROTEIN
**Protein name**	SECOND OB-FOLD DOMAIN OF REPLICATION PROTEIN A FROM METHANOCOCCUS MARIPALUDIS
**Deposition date**	30.06.2008
**PDB title**	SOLUTION NMR STRUCTURE OF THE SECOND OB-FOLD DOMAIN OF REPLICATION PROTEIN A FROM METHANOCOCCUS MARIPALUDIS. NORTHEAST STRUCTURAL GENOMICS TARGET MRR110B
**PDB authors**	J.M.ARAMINI, M.MAGLAQUI, M.JIANG, C.CICCOSANTI, R.XIAO, R.NAIR, J.K.EVERETT, G.VT.SWAPNA, T.B.ACTON, B.ROST, G.T.MONTELIONE, NORTHEAST STRUCTURAL GENOMICS CONSORTIUM (NESG)
**Last author**	MONTELIONE
**Reference**	
**Reference authors**	
**Reference title**	
**Software listed**	AUTOASSIGN, AUTOSTRUCTURE, CNS, CYANA, NMRPIPE, PDBSTAT, PSVS, SPARKY, TOPSPIN

**Spectrometer**	BRUKER (800 MHZ, 600 MHZ)

**Protein number**	44
**PDB code**	2MQL, doi:10.2210/pdb2MQL/pdb
**BMRB code**	25038, doi:10.13018/BMR25038
**UniProt code**	F2Z3R2, F2Z3R2_RAT
**PDB Header**	RNA BINDING PROTEIN
**Protein name**	STRUCTURAL INVESTIGATION OF HNRNP L
**Deposition date**	24.06.2014
**PDB title**	STRUCTURAL INVESTIGATION OF HNRNP L
**PDB authors**	M.BLATTER, F.ALLAIN
**Last author**	ALLAIN
**Reference**	J.MOL.BIOL. 427, 3001 (2015), doi:10.1016/J.JMB.2015.05.020
**Reference authors**	M.BLATTER, S.DUNIN-HORKAWICZ, I.GRISHINA, C.MARIS, S.THORE, T.MAIER, A.BINDEREIF, J.M.BUJNICKI, F.H.ALLAIN
**Reference title**	THE SIGNATURE OF THE FIVE-STRANDED VRRM FOLD DEFINED BY FUNCTIONAL, STRUCTURAL AND COMPUTATIONAL ANALYSIS OF THE HNRNP L PROTEIN
**Software listed**	AMBER, CYANA, SPARKY, TOPSPIN
**Spectrometer**	BRUKER (900 MHZ, 700 MHZ)

**Protein number**	45
**PDB code**	2K75, doi:10.2210/pdb2K75/pdb
**BMRB code**	15902, doi:10.13018/BMR15902
**UniProt code**	Q9HL44, Q9HL44_THEAC
**PDB Header**	DNA BINDING PROTEIN
**Protein name**	OB DOMAIN OF TA0387 FROM THERMOPLASMA ACIDOPHILUM
**Deposition date**	01.08.2008
**PDB title**	SOLUTION NMR STRUCTURE OF THE OB DOMAIN OF TA0387 FROM THERMOPLASMA ACIDOPHILUM. NORTHEAST STRUCTURAL GENOMICS CONSORTIUM TARGET TAR80B
**PDB authors**	T.A.RAMELOT, K.DING, D.LEE, M.JIANG, C.CICCOSANTI, R.XIAO, R.NAIR, J.K.EVERETT, G.SWAPNA, T.B.ACTON, B.ROST, G.T.MONTELIONE, M.A.KENNEDY, NORTHEAST STRUCTURAL GENOMICS CONSORTIUM (NESG)
**Last author**	KENNEDY
**Reference**	
**Reference authors**	
**Reference title**	
**Software listed**	AUTOASSIGN, AUTOSTRUCTURE, NMRPIPE, PSVS, SPARKY, TOPSPIN, VNMR, X-PLOR
**Spectrometer**	BRUKER, VARIAN (850 MHZ, 600 MHZ)

**Protein number**	46
**PDB code**	2LTM, doi:10.2210/pdb2LTM/pdb
**BMRB code**	18489, doi:10.13018/BMR18489
**UniProt code**	Q9UMS0, NFU1_HUMAN
**PDB Header**	ELECTRON TRANSPORT
**Protein name**	NFU1 IRON-SULFUR CLUSTER SCAFFOLD HOMOLOG FROM HOMO SAPIENS
**Deposition date**	29.05.2012
**PDB title**	SOLUTION NMR STRUCTURE OF NFU1 IRON-SULFUR CLUSTER SCAFFOLD HOMOLOG FROM HOMO SAPIENS, NORTHEAST STRUCTURAL GENOMICS CONSORTIUM (NESG) TARGET HR2876B
**PDB authors**	G.LIU, R.XIAO, H.JANJUA, K.HAMILTON, R.SHASTRY, E.KOHAN, T.B.ACTON, J.K.EVERETT, H.LEE, Y.J.HUANG, G.T.MONTELIONE, NORTHEAST STRUCTURAL GENOMICS CONSORTIUM (NESG), MITOCHONDRIAL PROTEIN PARTNERSHIP (MPP)
**Last author**	MONTELIONE
**Reference**	
**Reference authors**	
**Reference title**	
**Software listed**	AUTOASSIGN, AUTOSTRUCTURE, CNS, CYANA, NMRPIPE, PALES, PSVS, REDCAT, SPARKY, TALOS+, TOPSPIN, VNMRJ, XEASY
**Spectrometer**	BRUKER, VARIAN (800 MHZ, 600 MHZ)

**Protein number**	47
**PDB code**	2KOB, doi:10.2210/pdb2KOB/pdb
**BMRB code**	16498, doi:10.13018/BMR16498
**UniProt code**	A7VTE5, A7VTE5_9CLOT
**PDB Header**	STRUCTURAL GENOMICS, UNKNOWN FUNCTION
**Protein name**	CLOLEP_01837 (FRAGMENT 61-160) FROM CLOSTRIDIUM LEPTUM
**Deposition date**	15.09.2009
**PDB title**	SOLUTION NMR STRUCTURE OF CLOLEP_01837 (FRAGMENT 61-160) FROM CLOSTRIDIUM LEPTUM. NORTHEAST STRUCTURAL GENOMICS CONSORTIUM TARGET QLR8A
**PDB authors**	T.A.RAMELOT, D.LEE, C.CICCOSANTI, M.JIANG, R.NAIR, B.ROST, T.B.ACTON, R.XIAO, J.K.EVERETT, G.T.MONTELIONE, M.A.KENNEDY, NORTHEAST STRUCTURAL GENOMICS CONSORTIUM (NESG)
**Last author**	KENNEDY
**Reference**	
**Reference authors**	

**Reference title**	
**Software listed**	AUTOASSIGN, AUTOSTRUCTURE, CNS, NMRPIPE, PDBSTAT, PSVS, SPARKY, TOPSPIN, VNMR, X-PLOR
**Spectrometer**	BRUKER, VARIAN (850 MHZ, 600 MHZ)

**Protein number**	48
**PDB code**	2KHD, doi:10.2210/pdb2KHD/pdb
**BMRB code**	16238, doi:10.13018/BMR16238
**UniProt code**	Q9KL30, Q9KL30_VIBCH
**PDB Header**	STRUCTURAL GENOMICS, UNKNOWN FUNCTION
**Protein name**	VC_A0919 FROM VIBRIO CHOLERAE
**Deposition date**	02.04.2009
**PDB title**	SOLUTION NMR STRUCTURE OF VC_A0919 FROM VIBRIO CHOLERAE. NORTHEAST STRUCTURAL GENOMICS CONSORTIUM TARGET VCR52
**PDB authors**	T.A.RAMELOT, J.R.CORT, H.WANG, C.CICCOSANTI, M.JIANG, J.LIU, B.ROST, G.V.T.SWAPNA, T.B.ACTON, R.XIAO, J.K.EVERETT, G.T.MONTELIONE, M.A.KENNEDY, NORTHEAST STRUCTURAL GENOMICS CONSORTIUM (NESG)
**Last author**	KENNEDY
**Reference**	
**Reference authors**	
**Reference title**	
**Software listed**	AUTOASSIGN, AUTOSTRUCTURE, NMRPIPE, PDBSTAT, PSVS, SPARKY, TOPSPIN, VNMR, X-PLOR
**Spectrometer**	BRUKER, VARIAN (850 MHZ, 750 MHZ, 600 MHZ)

**Protein number**	49
**PDB code**	2RN7, doi:10.2210/pdb2RN7/pdb
**BMRB code**	11017, doi:10.13018/BMR11017
**UniProt code**	Q7UDG6, Q7UDG6_SHIFL
**PDB Header**	UNKNOWN FUNCTION
**Protein name**	TNPE PROTEIN FROM SHIGELLA FLEXNERI
**Deposition date**	08.12.2007
**PDB title**	NMR SOLUTION STRUCTURE OF TNPE PROTEIN FROM SHIGELLA FLEXNERI. NORTHEAST STRUCTURAL GENOMICS TARGET SFR125
**PDB authors**	T.A.RAMELOT, J.R.CORT, A.SEMESI, M.GARCIA, A.A.YEE, C.H.ARROWSMITH, M.A.KENNEDY, NORTHEAST STRUCTURAL GENOMICS CONSORTIUM (NESG)
**Last author**	KENNEDY
**Reference**	
**Reference authors**	
**Reference title**	
**Software listed**	AUTOSTRUCTURE, CNS, NMRPIPE, SPARKY, VNMR, X-PLOR
**Spectrometer**	VARIAN (750 MHZ, 600 MHZ)

**Protein number**	50
**PDB code**	2LXU, doi:10.2210/pdb2LXU/pdb
**BMRB code**	18698, doi:10.13018/BMR18698
**UniProt code**	P31943, HNRH1_HUMAN
**PDB Header**	RNA BINDING PROTEIN
**Protein name**	EUKARYOTIC RNA RECOGNITION MOTIF, RRM1, FROM HETEROGENEOUS NUCLEAR RIBONUCLEOPROTEIN H FROM HOMO SAPIENS
**Deposition date**	31.08.2012
**PDB title**	SOLUTION NMR STRUCTURE OF THE EUKARYOTIC RNA RECOGNITION MOTIF, RRM1, FROM THE HETEROGENEOUS NUCLEAR RIBONUCLEOPROTEIN H FROM HOMO SAPIENS, NORTHEAST STRUCTURAL GENOMICS CONSORTIUM (NESG) TARGET HR8614A
**PDB authors**	T.A.RAMELOT, Y.YANG, K.PEDERSON, R.SHASTRY, E.KOHAN, H.JANJUA, R.XIAO, T.B.ACTON, J.K.EVERETT, J.H.PRESTEGARD, G.T.MONTELIONE, M.A.KENNEDY, NORTHEAST STRUCTURAL GENOMICS CONSORTIUM (NESG)
**Last author**	KENNEDY
**Reference**	
**Reference authors**	
**Reference title**	
**Software listed**	AUTOSTRUCTURE, CNS, CYANA, FMCGUI, NMRPIPE, PALES, PINE, PSVS, SPARKY, TALOS+, TOPSPIN, VNMRJ
**Spectrometer**	BRUKER, VARIAN (800 MHZ, 600 MHZ)

**Protein number**	51
**PDB code**	2KIF, doi:10.2210/pdb2KIF/pdb
**BMRB code**	16272, doi:10.13018/BMR16272
**UniProt code**	A6B4U8, A6B4U8_VIBPA
**PDB Header**	TRANSFERASE
**Protein name**	AN O6-METHYLGUANINE DNA METHYLTRANSFERASE FAMILY PROTEIN FROM VIBRIO PARAHAEMOLYTICUS
**Deposition date**	03.05.2009
**PDB title**	SOLUTION NMR STRUCTURE OF AN O6-METHYLGUANINE DNA METHYLTRANSFERASE FAMILY PROTEIN FROM VIBRIO PARAHAEMOLYTICUS. NORTHEAST STRUCTURAL GENOMICS CONSORTIUM TARGET VPR247
**PDB authors**	J.M.ARAMINI, R.L.BELOTE, C.T.CICCOSANTI, M.JIANG, B.ROST, R.NAIR, G.V.T.SWAPNA, T.B.ACTON, R.XIAO, J.K.EVERETT, G.T.MONTELIONE, NORTHEAST STRUCTURAL GENOMICS CONSORTIUM (NESG)
**Last author**	MONTELIONE

**Reference**	J.BIOL.CHEM. 285, 13736 (2010), doi:10.1074/JBC.M109.093591
**Reference authors**	J.M.ARAMINI, J.L.TUBBS, S.KANUGULA, P.ROSSI, A.ERTEKIN, M.MAGLAQUI, K.HAMILTON, C.T.CICCOSANTI, M.JIANG, R.XIAO, T.T.SOONG, B.ROST, T.B.ACTON, J.K.EVERETT, A.E.PEGG, J.A.TAINER, G.T.MONTELIONE
**Reference title**	STRUCTURAL BASIS OF O6-ALKYLGUANINE RECOGNITION BY A BACTERIAL ALKYLTRANSFERASE-LIKE DNA REPAIR PROTEIN
**Software listed**	AUTOASSIGN, AUTOSTRUCTURE, CNS, CYANA, MOLPROBITY, NMRPIPE, PDBSTAT, PINE, PSVS, SPARKY, TOPSPIN
**Spectrometer**	BRUKER (800 MHZ, 600 MHZ)

**Protein number**	52
**PDB code**	2KBN, doi:10.2210/pdb2KBN/pdb
**BMRB code**	16051, doi:10.13018/BMR16051
**UniProt code**	Q8Q045, Q8Q045_METMA
**PDB Header**	STRUCTURAL GENOMICS, UNKNOWN FUNCTION
**Protein name**	OB DOMAIN (RESIDUES 67-166) OF MM0293 FROM METHANOSARCINA MAZEI
**Deposition date**	03.12.2008
**PDB title**	SOLUTION NMR STRUCTURE OF THE OB DOMAIN (RESIDUES 67-166) OF MM0293 FROM METHANOSARCINA MAZEI. NORTHEAST STRUCTURAL GENOMICS CONSORTIUM TARGET MAR214A
**PDB authors**	T.A.RAMELOT, K.DING, M.MAGLIQUI, M.JIANG, C.CICCOSANTI, R.XIAO, J.LUI, J.K.EVERETT, G.SWAPNA, T.B.ACTON, B.ROST, G.T.MONTELIONE, M.A.KENNEDY, NORTHEAST STRUCTURAL GENOMICS CONSORTIUM (NESG)
**Last author**	KENNEDY
**Reference**	
**Reference authors**	
**Reference title**	
**Software listed**	AUTOASSIGN, AUTOSTRUCTURE, CNS, CYANA, PSVS, SPARKY, TOPSPIN, X-PLOR
**Spectrometer**	BRUKER, VARIAN (850 MHZ, 600 MHZ)

**Protein number**	53
**PDB code**	2MK2, doi:10.2210/pdb2MK2/pdb
**BMRB code**	19749, doi:10.13018/BMR19749
**UniProt code**	O15357, SHIP2_HUMAN
**PDB Header**	HYDROLASE
**Protein name**	N-TERMINAL DOMAIN (SH2 DOMAIN) OF HUMAN INOSITOL POLYPHOSPHATE PHOSPHATASE-LIKE PROTEIN 1 (INPPL1) (FRAGMENT 20-117)
**Deposition date**	23.01.2014
**PDB title**	SOLUTION NMR STRUCTURE OF N-TERMINAL DOMAIN (SH2 DOMAIN) OF HUMAN INOSITOL POLYPHOSPHATE PHOSPHATASE-LIKE PROTEIN 1 (INPPL1) (FRAGMENT 20-117), NORTHEAST STRUCTURAL GENOMICS CONSORTIUM TARGET HR9134A
**PDB authors**	Y.YANG, T.A.RAMELOT, H.JANJUA, R.XIAO, J.K.EVERETT, G.T.MONTELIONE, M.A.KENNEDY, NORTHEAST STRUCTURAL GENOMICS CONSORTIUM (NESG)
**Last author**	KENNEDY
**Reference**	
**Reference authors**	
**Reference title**	
**Software listed**	AUTOASSIGN, AUTOSTRUCTURE, CNS, CYANA, NMRPIPE, PALES, PINE, PSVS, REDCAT, SPARKY, TALOS+, TOPSPIN, VNMRJ, XEASY
**Spectrometer**	BRUKER, VARIAN (850 MHZ, 600 MHZ)

**Protein number**	54
**PDB code**	2K50, doi:10.2210/pdb2K50/pdb
**BMRB code**	15819, doi:10.13018/BMR15819
**UniProt code**	O27438, O27438_METTH
**PDB Header**	STRUCTURAL GENOMICS, UNKNOWN FUNCTION
**Protein name**	REPLICATION FACTOR A RELATED PROTEIN FROM METHANOBACTERIUM THERMOAUTOTROPHICUM
**Deposition date**	23.06.2008
**PDB title**	SOLUTION NMR STRUCTURE OF THE REPLICATION FACTOR A RELATED PROTEIN FROM METHANOBACTERIUM THERMOAUTOTROPHICUM. NORTHEAST STRUCTURAL GENOMICS TARGET TR91A
**PDB authors**	P.ROSSI, R.XIAO, M.MAGLAQUI, E.L.FOOTE, C.CICCOSANTI, G.SWAPNA, T.B.ACTON, B.ROST, J.K.EVERETT, M.JIANG, R.NAIR, G.T.MONTELIONE, NORTHEAST STRUCTURAL GENOMICS CONSORTIUM (NESG)
**Last author**	MONTELIONE
**Reference**	
**Reference authors**	
**Reference title**	
**Software listed**	AUTOASSIGN, CNS, CYANA, MOLMOL, MOLPROBITY, NMRPIPE, PROCHECK, PSVS, RPF(AUTOSTRUCTURE), SPARKY, TALOS, TOPSPIN
**Spectrometer**	BRUKER (800 MHZ, 600 MHZ)

**Protein number**	55
**PDB code**	2KL5, doi:10.2210/pdb2KL5/pdb
**BMRB code**	16384, doi:10.13018/BMR16384
**UniProt code**	O32127, O32127_BACSU
**PDB Header**	STRUCTURAL GENOMICS, UNKNOWN FUNCTION
**Protein name**	PROTEIN YUTD FROM B.SUBTILIS

**Deposition date**	30.06.2009
**PDB title**	SOLUTION NMR STRUCTURE OF PROTEIN YUTD FROM B.SUBTILIS, NORTHEAST STRUCTURAL GENOMICS CONSORTIUM TARGET SR232
**PDB authors**	G.LIU, K.HAMILTON, R.XIAO, C.CICCOSANTI, C.J.HO, J.EVERETT, R.NAIR, T.ACTON, B.ROST, G.T.MONTELIONE, NORTHEAST STRUCTURAL GENOMICS CONSORTIUM (NESG)
**Last author**	MONTELIONE
**Reference**	
**Reference authors**	
**Reference title**	
**Software listed**	AUTOASSIGN, AUTOSTRUCTURE, CNS, CYANA, NMRPIPE, TOPSPIN, VNMRJ, XEASY
**Spectrometer**	BRUKER, VARIAN (800 MHZ, 600 MHZ)

**Protein number**	56
**PDB code**	2LTA, doi:10.2210/pdb2LTA/pdb
**BMRB code**	18465, doi:10.13018/BMR18465
**UniProt code**	
**PDB Header**	DE NOVO PROTEIN
**Protein name**	DE NOVO DESIGNED PROTEIN, ROSSMANN 3X1 FOLD
**Deposition date**	15.05.2012
**PDB title**	SOLUTION NMR STRUCTURE OF DE NOVO DESIGNED PROTEIN, ROSSMANN 3X1 FOLD, NORTHEAST STRUCTURAL GENOMICS CONSORTIUM TARGET OR157
**PDB authors**	G.LIU, R.KOGA, N.KOGA, R.XIAO, K.PEDERSON, K.HAMILTON, E.KOHAN, T.B.ACTON, G.KORNHABER, J.K.EVERETT, D.BAKER, G.T.MONTELIONE, NORTHEAST STRUCTURAL GENOMICS CONSORTIUM (NESG)
**Last author**	MONTELIONE
**Reference**	NATURE 491, 222 (2012), doi:10.1038/NATURE11600
**Reference authors**	N.KOGA, R.TATSUMI-KOGA, G.LIU, R.XIAO, T.B.ACTON, G.T.MONTELIONE, D.BAKER
**Reference title**	PRINCIPLES FOR DESIGNING IDEAL PROTEIN STRUCTURES
**Software listed**	AUTOASSIGN, AUTOSTRUCTURE, CNS, CYANA, NMRPIPE, PSVS, REDCAT, SPARKY, TALOS+, TOPSPIN, VNMRJ, XEASY
**Spectrometer**	BRUKER, VARIAN (800 MHZ, 600 MHZ)

**Protein number**	57
**PDB code**	2KIW, doi:10.2210/pdb2KIW/pdb
**BMRB code**	16298, doi:10.13018/BMR16298
**UniProt code**	Q4L7R3, Q4L7R3_STAHJ
**PDB Header**	DNA BINDING PROTEIN
**Protein name**	DOMAIN N-TERMINAL TO INTEGRASE DOMAIN OF SH1003 FROM STAPHYLOCOCCUS HAEMOLYTICUS
**Deposition date**	12.05.2009
**PDB title**	SOLUTION NMR STRUCTURE OF THE DOMAIN N-TERMINAL TO THE INTEGRASE DOMAIN OF SH1003 FROM STAPHYLOCOCCUS HAEMOLYTICUS. NORTHEAST STRUCTURAL GENOMICS CONSORTIUM TARGET SHR105F (64-166)
**PDB authors**	Y.YANG, T.A.RAMELOT, R.L.BELOTE, E.L.FOOTE, H.JANJUA, R.NAIR, B.ROST, G.SWAPNA, T.B.ACTON, R.XIAO, J.K.EVERETT, G.T.MONTELIONE, M.A.KENNEDY, NORTHEAST STRUCTURAL GENOMICS CONSORTIUM (NESG)
**Last author**	KENNEDY
**Reference**	
**Reference authors**	
**Reference title**	
**Software listed**	AUTOASSIGN, AUTOSTRUCTURE, CNS, NMRPIPE, PDBSTAT, PSVS, SPARKY, TOPSPIN, VNMR, X-PLOR
**Spectrometer**	BRUKER, VARIAN (850 MHZ, 600 MHZ)

**Protein number**	58
**PDB code**	2LVB, doi:10.2210/pdb2LVB/pdb
**BMRB code**	18561, doi:10.13018/BMR18561
**UniProt code**	
**PDB Header**	DE NOVO PROTEIN
**Protein name**	SOLUTION NMR STRUCTURE DE NOVO DESIGNED PFK FOLD PROTEIN
**Deposition date**	30.06.2012
**PDB title**	SOLUTION NMR STRUCTURE DE NOVO DESIGNED PFK FOLD PROTEIN, NORTHEAST STRUCTURAL GENOMICS CONSORTIUM (NESG) TARGET OR250
**PDB authors**	G.LIU, N.KOGA, R.KOGA, R.XIAO, K.HAMILTON, E.KOHAN, T.B.ACTON, G.KORNHABER, J.K.EVERETT, D.BAKER, G.T.MONTELIONE, NORTHEAST STRUCTURAL GENOMICS CONSORTIUM (NESG)
**Last author**	MONTELIONE
**Reference**	NATURE 491, 222 (2012), doi:10.1038/NATURE11600
**Reference authors**	N.KOGA, R.TATSUMI-KOGA, G.LIU, R.XIAO, T.B.ACTON, G.T.MONTELIONE, D.BAKER
**Reference title**	PRINCIPLES FOR DESIGNING IDEAL PROTEIN STRUCTURES
**Software listed**	AUTOASSIGN, AUTOSTRUCTURE, CNS, CYANA, NMRPIPE, PSVS, SPARKY, TALOS+, TOPSPIN, VNMRJ, XEASY
**Spectrometer**	BRUKER, VARIAN (800 MHZ, 600 MHZ)

**Protein number**	59
**PDB code**	2LND, doi:10.2210/pdb2LND/pdb
**BMRB code**	18161, doi:10.13018/BMR18161
**UniProt code**	

**PDB Header**	DE NOVO PROTEIN
**Protein name**	DE NOVO DESIGNED PROTEIN, PFK FOLD
**Deposition date**	23.12.2011
**PDB title**	SOLUTION NMR STRUCTURE OF DE NOVO DESIGNED PROTEIN, PFK FOLD, NORTHEAST STRUCTURAL GENOMICS CONSORTIUM TARGET OR134
**PDB authors**	G.LIU, N.KOGA, R.KOGA, R.XIAO, H.LEE, H.JANJUA, E.KOHAN, T.B.ACTON, J.K.EVERETT, D.BAKER, G.T.MONTELIONE, NORTHEAST STRUCTURAL GENOMICS CONSORTIUM (NESG)
**Last author**	MONTELIONE
**Reference**	
**Reference authors**	
**Reference title**	
**Software listed**	AUTOASSIGN, AUTOSTRUCTURE, CNS, CYANA, NMRPIPE, PSVS, SPARKY, TALOS+, TOPSPIN, VNMRJ, XEASY
**Spectrometer**	BRUKER, VARIAN (800 MHZ, 600 MHZ)

**Protein number**	60
**PDB code**	1WQU, doi:10.2210/pdb1WQU/pdb
**BMRB code**	6331, doi:10.13018/BMR6331
**UniProt code**	P07332, FES_HUMAN
**PDB Header**	TRANSFERASE
**Protein name**	HUMAN FES SH2 DOMAIN
**Deposition date**	02.10.2004
**PDB title**	SOLUTION STRUCTURE OF THE HUMAN FES SH2 DOMAIN
**PDB authors**	A.SCOTT, D.PANTOJA-UCEDA, S.KOSHIBA, M.INOUE, T.KIGAWA, T.TERADA, M.SHIROUZU, A.TANAKA, S.SUGANO, S.YOKOYAMA, P.GUNTERT, RIKEN STRUCTURAL GENOMICS/PROTEOMICS INITIATIVE (RSGI)
**Last author**	GUNTERT
**Reference**	J.BIOMOL.NMR 31, 357 (2005), doi:10.1007/S10858-005-0946-6
**Reference authors**	A.SCOTT, D.PANTOJA-UCEDA, S.KOSHIBA, M.INOUE, T.KIGAWA, T.TERADA, M.SHIROUZU, A.TANAKA, S.SUGANO, S.YOKOYAMA, P.GUNTERT
**Reference title**	SOLUTION STRUCTURE OF THE SRC HOMOLOGY 2 DOMAIN FROM THE HUMAN FELINE SARCOMA ONCOGENE FES
**Software listed**	CYANA, NMRPIPE, NMRVIEW, OPALP
**Spectrometer**	BRUKER (800 MHZ, 600 MHZ)

**Protein number**	61
**PDB code**	2KL6, doi:10.2210/pdb2KL6/pdb
**BMRB code**	16385, doi:10.13018/BMR16385
**UniProt code**	Q8U1U6, Q8U1U6_PYRFU
**PDB Header**	STRUCTURAL GENOMICS, UNKNOWN FUNCTION
**Protein name**	CARDB DOMAIN OF PF1109 FROM PYROCOCCUS FURIOSUS
**Deposition date**	30.06.2009
**PDB title**	SOLUTION NMR STRUCTURE OF THE CARDB DOMAIN OF PF1109 FROM PYROCOCCUS FURIOSUS. NORTHEAST STRUCTURAL GENOMICS CONSORTIUM TARGET PFR193A
**PDB authors**	J.M.ARAMINI, D.LEE, C.CICCOSANTI, K.HAMILTON, R.NAIR, B.ROST, T.B.ACTON, R.XIAO, G.V.T.SWAPNA, J.K.EVERETT, G.T.MONTELIONE, NORTHEAST STRUCTURAL GENOMICS CONSORTIUM (NESG)
**Last author**	MONTELIONE
**Reference**	
**Reference authors**	
**Reference title**	
**Software listed**	AUTOSTRUCTURE, CNS, CYANA, MOLPROBITY, NMRPIPE, PDBSTAT, PINE, PSVS, SPARKY, TALOS, TOPSPIN
**Spectrometer**	BRUKER (800 MHZ, 600 MHZ)

**Protein number**	62
**PDB code**	6GT7, doi:10.2210/pdb6GT7/pdb
**BMRB code**	34287, doi:10.13018/BMR34287
**UniProt code**	Q54324, Q54324_SULIS
**PDB Header**	TRANSFERASE
**Protein name**	FREE HELIX BUNDLE DOMAIN FROM FUNCTIONAL PRN1 PRIMASE
**Deposition date**	15.06.2018
**PDB title**	NMR STRUCTURE OF THE FREE HELIX BUNDLE DOMAIN FROM THE FUNCTIONAL PRN1 PRIMASE
**PDB authors**	J.BOUDET, G.LIPPS, F.ALLAIN
**Last author**	ALLAIN
**Reference**	CELL 176, 154 (2019), doi:10.1016/J.CELL.2018.11.031
**Reference authors**	J.BOUDET, J.C.DEVILLIER, T.WIEGAND, L.SALMON, B.H.MEIER, G.LIPPS, F.H.ALLAIN
**Reference title**	A SMALL HELICAL BUNDLE PREPARES PRIMER SYNTHESIS BY BINDING TWO NUCLEOTIDES THAT ENHANCE SEQUENCE-SPECIFIC RECOGNITION OF THE DNA TEMPLATE
**Software listed**	AMBER, CANDID, CYANA, SPARKY
**Spectrometer**	BRUKER (900 MHZ, 700 MHZ, 600 MHZ)

**Protein number**	63
**PDB code**	2JN8, doi:10.2210/pdb2JN8/pdb
**BMRB code**	15089, doi:10.13018/BMR15089
**UniProt code**	Q8ZRJ2, Q8ZRJ2_SALTY
**PDB Header**	STRUCTURAL GENOMICS, UNKNOWN FUNCTION

**Protein name**	Q8ZRJ2 FROM SALMONELLA TYPHIMURIUM
**Deposition date**	29.12.2006
**PDB title**	SOLUTION NMR STRUCTURE OF Q8ZRJ2 FROM SALMONELLA TYPHIMURIUM. NORTHEAST STRUCTURAL GENOMICS TARGET STR65
**PDB authors**	J.M.ARAMINI, J.R.CORT, C.K.HO, K.CUNNINGHAM, L.-C.MA, R.XIAO, J.LIU, M.C.BARAN, G.V.T.SWAPNA, T.B.ACTON, B.ROST, G.T.MONTELIONE, NORTHEAST STRUCTURAL GENOMICS CONSORTIUM (NESG)
**Last author**	MONTELIONE
**Reference**	
**Reference authors**	
**Reference title**	
**Software listed**	AUTOASSIGN, AUTOSTRUCTURE, BRUKER, NMRPIPE, PDBSTAT, PSVS, SPARKY, VNMR, X-PLOR
**Spectrometer**	BRUKER, VARIAN (750 MHZ, 600 MHZ, 500 MHZ)

**Protein number**	64
**PDB code**	2K5D, doi:10.2210/pdb2K5D/pdb
**BMRB code**	15829, doi:10.13018/BMR15829
**UniProt code**	Q8E006, Q8E006_STRA5
**PDB Header**	STRUCTURAL GENOMICS, UNKNOWN FUNCTION
**Protein name**	SAG0934 FROM STREPTOCOCCUS AGALACTIAE
**Deposition date**	26.06.2008
**PDB title**	SOLUTION NMR STRUCTURE OF SAG0934 FROM STREPTOCOCCUS AGALACTIAE. NORTHEAST STRUCTURAL GENOMICS TARGET SAR32[1-108]
**PDB authors**	J.M.ARAMINI, P.ROSSI, L.ZHAO, E.L.FOOTE, M.JIANG, R.XIAO, S.SHARMA, G.VT.SWAPNA, R.NAIR, J.K.EVERETT, T.B.ACTON, B.ROST, G.T.MONTELIONE, NORTHEAST STRUCTURAL GENOMICS CONSORTIUM (NESG)
**Last author**	MONTELIONE
**Reference**	
**Reference authors**	
**Reference title**	
**Software listed**	AUTOASSIGN, AUTOSTRUCTURE, CNS, CYANA, NMRPIPE, PDBSTAT, PSVS, SPARKY, TOPSPIN
**Spectrometer**	BRUKER (800 MHZ, 600 MHZ)

**Protein number**	65
**PDB code**	2KD1, doi:10.2210/pdb2KD1/pdb
**BMRB code**	16102, doi:10.13018/BMR16102
**UniProt code**	Q81GD4, Q81GD4_BACCR
**PDB Header**	STRUCTURAL GENOMICS, UNKNOWN FUNCTION
**Protein name**	INTEGRASE-LIKE DOMAIN FROM BACILLUS CEREUS ORDERED LOCUS BC_1272
**Deposition date**	31.12.2008
**PDB title**	SOLUTION NMR STRUCTURE OF THE INTEGRASE-LIKE DOMAIN FROM BACILLUS CEREUS ORDERED LOCUS BC_1272. NORTHEAST STRUCTURAL GENOMICS CONSORTIUM TARGET BCR268F
**PDB authors**	P.ROSSI, H.LEE, M.MAGLAQUI, E.L.FOOTE, W.A.BUCHWALD, M.JIANG, G.V.T.SWAPNA, R.NAIR, R.XIAO, T.B.ACTON, B.ROST, J.H.PRESTEGARD, G.T.MONTELIONE, NORTHEAST STRUCTURAL GENOMICS CONSORTIUM (NESG)
**Last author**	MONTELIONE
**Reference**	
**Reference authors**	
**Reference title**	
**Software listed**	CNS, CYANA, MOLMOL, MOLPROBITY, NMRPIPE, PDBSTAT, PINE, PROCHECK, PSVS, SPARKY, TALOS, TOPSPIN
**Spectrometer**	BRUKER, VARIAN (800 MHZ, 600 MHZ)

**Protein number**	66
**PDB code**	2LTL, doi:10.2210/pdb2LTL/pdb
**BMRB code**	18487, doi:10.13018/BMR18487
**UniProt code**	P32860, NFU1_YEAST
**PDB Header**	ELECTRON TRANSPORT
**Protein name**	NIFU-LIKE PROTEIN FROM SACCHAROMYCES CEREVISIAE
**Deposition date**	29.05.2012
**PDB title**	SOLUTION NMR STRUCTURE OF NIFU-LIKE PROTEIN FROM SACCHAROMYCES CEREVISIAE, NORTHEAST STRUCTURAL GENOMICS CONSORTIUM (NESG) TARGET YR313A
**PDB authors**	G.LIU, R.XIAO, K.HAMILTON, H.JANJUA, R.SHASTRY, E.KOHAN, T.B.ACTON, J.K.EVERETT, H.LEE, Y.J.HUANG, G.T.MONTELIONE, NORTHEAST STRUCTURAL GENOMICS CONSORTIUM (NESG), MITOCHONDRIAL PROTEIN PARTNERSHIP (MPP)
**Last author**	MONTELIONE
**Reference**	
**Reference authors**	
**Reference title**	
**Software listed**	AUTOASSIGN, AUTOSTRUCTURE, CNS, CYANA, NMRPIPE, PALES, PINE, PSVS, REDCAT, SPARKY, TALOS+, TOPSPIN, VNMRJ, XEASY
**Spectrometer**	BRUKER, VARIAN (800 MHZ, 600 MHZ)

**Protein number**	67
**PDB code**	2KVO, doi:10.2210/pdb2KVO/pdb
**BMRB code**	16782, doi:10.13018/BMR16782

**UniProt code**	Q55356, PSB28_SYNY3
**PDB Header**	PHOTOSYNTHESIS
**Protein name**	PHOTOSYSTEM II REACTION CENTER PSB28 PROTEIN FROM SYNECHOCYSTIS SP.(STRAIN PCC 6803)
**Deposition date**	22.03.2010
**PDB title**	SOLUTION NMR STRUCTURE OF PHOTOSYSTEM II REACTION CENTER PSB28 PROTEIN FROM SYNECHOCYSTIS SP.(STRAIN PCC 6803), NORTHEAST STRUCTURAL GENOMICS CONSORTIUM TARGET SGR171
**PDB authors**	Y.YANG, T.A.RAMELOT, J.R.CORT, D.WANG, C.CICCOSANTI, K.HAMILTON, R.NAIR, B.ROST, T.B.ACTON, R.XIAO, J.K.EVERETT, G.T.MONTELIONE, M.A.KENNEDY, NORTHEAST STRUCTURAL GENOMICS CONSORTIUM (NESG)
**Last author**	KENNEDY
**Reference**	PROTEINS 79, 340 (2011), doi:10.1002/PROT.22876
**Reference authors**	Y.YANG, T.A.RAMELOT, J.R.CORT, D.WANG, C.CICCOSANTI, K.HAMILTON, R.NAIR, B.ROST, T.B.ACTON, R.XIAO, J.K.EVERETT, G.T.MONTELIONE, M.A.KENNEDY
**Reference title**	SOLUTION NMR STRUCTURE OF PHOTOSYSTEM II REACTION CENTER PROTEIN PSB28 FROM SYNECHOCYSTIS SP. STRAIN PCC 6803
**Software listed**	AUTOASSIGN, AUTOSTRUCTURE, CNS, NMRPIPE, PDBSTAT, PINE_SERVER, PSVS, SPARKY, TOPSPIN, VNMR, X-PLOR_NIH
**Spectrometer**	BRUKER, VARIAN (850 MHZ, 600 MHZ)

**Protein number**	68
**PDB code**	1T0Y, doi:10.2210/pdb1T0Y/pdb
**BMRB code**	6176, doi:10.13018/BMR6176
**UniProt code**	Q20728, YXHK_CAEEL
**PDB Header**	CHAPERONE
**Protein name**	A UBIQUITIN-LIKE DOMAIN FROM TUBULIN-BINDING COFACTOR B
**Deposition date**	13.04.2004
**PDB title**	SOLUTION STRUCTURE OF A UBIQUITIN-LIKE DOMAIN FROM TUBULIN-BINDING COFACTOR B
**PDB authors**	B.L.LYTLE, F.C.PETERSON, S.H.QUI, M.LUO, B.F.VOLKMAN, J.L.MARKLEY, CENTER FOR EUKARYOTIC STRUCTURAL GENOMICS (CESG)
**Last author**	MARKLEY
**Reference**	J.BIOL.CHEM. 279, 46787 (2004), doi:10.1074/JBC.M409422200
**Reference authors**	B.L.LYTLE, F.C.PETERSON, S.H.QIU, M.LUO, Q.ZHAO, J.L.MARKLEY, B.F.VOLKMAN
**Reference title**	SOLUTION STRUCTURE OF A UBIQUITIN-LIKE DOMAIN FROM TUBULIN-BINDING COFACTOR B
**Software listed**	CYANA, NMRPIPE, SPSCAN, X-PLOR_NIH, XEASY, XWINNMR
**Spectrometer**	BRUKER (600 MHZ)

**Protein number**	69
**PDB code**	2KCD, doi:10.2210/pdb2KCD/pdb
**BMRB code**	16072, doi:10.13018/BMR16072
**UniProt code**	Q4A134, Q4A134_STAS1
**PDB Header**	STRUCTURAL GENOMICS, UNKNOWN FUNCTION
**Protein name**	SSP0047 FROM STAPHYLOCOCCUS SAPROPHYTICUS
**Deposition date**	19.12.2008
**PDB title**	SOLUTION NMR STRUCTURE OF SSP0047 FROM STAPHYLOCOCCUS SAPROPHYTICUS. NORTHEAST STRUCTURAL GENOMICS CONSORTIUM TARGET SYR6
**PDB authors**	T.A.RAMELOT, K.DING, C.X.CHEN, M.JIANG, C.CICCOSANTI, R.XIAO, J.LIU, M.C.BARAN, G.SWAPNA, T.B.ACTON, B.ROST, G.T.MONTELIONE, M.A.KENNEDY, NORTHEAST STRUCTURAL GENOMICS CONSORTIUM (NESG)
**Last author**	KENNEDY
**Reference**	
**Reference authors**	
**Reference title**	
**Software listed**	AUTOASSIGN, AUTOSTRUCTURE, NMRPIPE, PDBSTAT, PSVS, SPARKY, TOPSPIN, VNMR, X-PLOR
**Spectrometer**	BRUKER, VARIAN (850 MHZ, 750 MHZ, 600 MHZ)

**Protein number**	70
**PDB code**	2KRT, doi:10.2210/pdb2KRT/pdb
**BMRB code**	16648, doi:10.13018/BMR16648
**UniProt code**	Q9PRA0, Q9PRA0_UREPA
**PDB Header**	LIPID BINDING PROTEIN
**Protein name**	A CONSERVED HYPOTHETICAL MEMBRANE LIPOPROTEIN OBTAINED FROM UREAPLASMA PARVUM
**Deposition date**	22.12.2009
**PDB title**	SOLUTION NMR STRUCTURE OF A CONSERVED HYPOTHETICAL MEMBRANE LIPOPROTEIN OBTAINED FROM UREAPLASMA PARVUM: NORTHEAST STRUCTURAL GENOMICS CONSORTIUM TARGET UUR17A (139-239)
**PDB authors**	R.MANI, G.SWAPNA, H.JANJUA, C.CICCOSANTI, Y.HUANG, D.PATEL, R.XIAO, T.ACTON, J.EVERETT, G.T.MONTELIONE, NORTHEAST STRUCTURAL GENOMICS CONSORTIUM (NESG)
**Last author**	MONTELIONE
**Reference**	
**Reference authors**	
**Reference title**	
**Software listed**	AUTOASSIGN, AUTOSTRUCTURE, CNS, CYANA, PINE
**Spectrometer**	BRUKER, VARIAN (800 MHZ, 600 MHZ, 500 MHZ)

**Protein number**	71

**PDB code**	2LFI, doi:10.2210/pdb2LFI/pdb
**BMRB code**	17754, doi:10.13018/BMR17754
**UniProt code**	
**PDB Header**	METAL BINDING PROTEIN
**Protein name**	A MUCBP DOMAIN (FRAGMENT 187-294) OF PROTEIN LBA1460 FROM LACTOBACILLUS ACIDOPHILUS
**Deposition date**	30.06.2011
**PDB title**	SOLUTION NMR STRUCTURE OF A MUCBP DOMAIN (FRAGMENT 187-294) OF THE PROTEIN LBA1460 FROM LACTOBACILLUS ACIDOPHILUS, NORTHEAST STRUCTURAL GENOMICS CONSORTIUM TARGET LAR80A
**PDB authors**	E.A.FELDMANN, T.A.RAMELOT, Y.YANG, H.LEE, C.CICCOSANTI, H.JANJUA, R.NAIR, T.B.ACTON, R.XIAO, J.K.EVERETT, J.H.PRESTEGARD, G.T.MONTELIONE, M.A.KENNEDY, NORTHEAST STRUCTURAL GENOMICS CONSORTIUM (NESG)
**Last author**	KENNEDY
**Reference**	
**Reference authors**	
**Reference title**	
**Software listed**	AUTOSTRUCTURE, CNS, CYANA, NMRPIPE, PDBSTAT, PINE_SERVER, PSVS, SPARKY, TOPSPIN, VNMR, X-PLOR_NIH
**Spectrometer**	BRUKER, VARIAN (850 MHZ, 600 MHZ)

**Protein number**	72
**PDB code**	2JQN, doi:10.2210/pdb2JQN/pdb
**BMRB code**	15281, doi:10.13018/BMR15281
**UniProt code**	Q9AAR9, Q9AAR9_CAUCR
**PDB Header**	STRUCTURAL GENOMICS
**Protein name**	CC0527 FROM CAULOBACTER CRESCENTUS
**Deposition date**	05.06.2007
**PDB title**	SOLUTION NMR STRUCTURE OF CC0527 FROM CAULOBACTER CRESCENTUS. NORTHEAST STRUCTURAL GENOMICS TARGET CCR55
**PDB authors**	J.M.ARAMINI, P.ROSSI, H.N.B.MOSELEY, D.WANG, C.NWOSU, K.CUNNINGHAM, L.MA, R.XIAO, J.LIU, M.C.BARAN, G.V.T.SWAPNA, T.B.ACTON, B.ROST, G.T.MONTELIONE, NORTHEAST STRUCTURAL GENOMICS CONSORTIUM (NESG)
**Last author**	MONTELIONE
**Reference**	
**Reference authors**	
**Reference title**	
**Software listed**	AUTOASSIGN, AUTOSTRUCTURE, CNS, NMRPIPE, PDBSTAT, PSVS, SPARKY, TOPSPIN, VNMR, X-PLOR
**Spectrometer**	BRUKER, VARIAN (800 MHZ, 600 MHZ)

**Protein number**	73
**PDB code**	2L7Q, doi:10.2210/pdb2L7Q/pdb
**BMRB code**	17370, doi:10.13018/BMR17370
**UniProt code**	A6L0P5, A6L0P5_BACV8
**PDB Header**	STRUCTURAL GENOMICS, UNKNOWN FUNCTION
**Protein name**	CONJUGATE TRANSPOSON PROTEIN BVU_1572(27- 141) FROM BACTEROIDES VULGATUS
**Deposition date**	20.12.2010
**PDB title**	SOLUTION NMR STRUCTURE OF CONJUGATE TRANSPOSON PROTEIN BVU_1572(27- 141) FROM BACTEROIDES VULGATUS, NORTHEAST STRUCTURAL GENOMICS CONSORTIUM TARGET BVR155
**PDB authors**	Y.YANG, T.A.RAMELOT, J.R.CORT, D.WANG, C.CICCOSANTI, H.JANJUA, T.B.ACTON, R.XIAO, J.K.EVERETT, G.T.MONTELIONE, M.A.KENNEDY, NORTHEAST STRUCTURAL GENOMICS CONSORTIUM (NESG)
**Last author**	KENNEDY
**Reference**	PROTEINS 80, 667 (2012), doi:10.1002/PROT.23235
**Reference authors**	T.A.RAMELOT, Y.YANG, R.XIAO, T.B.ACTON, J.K.EVERETT, G.T.MONTELIONE, M.A.KENNEDY
**Reference title**	SOLUTION NMR STRUCTURE OF BT_0084, A CONJUGATIVE TRANSPOSON LIPOPROTEIN FROM BACTEROIDES THETAIOTAMICRON
**Software listed**	AUTOASSIGN, AUTOSTRUCTURE, CNS, CYANA, NMRPIPE, PDBSTAT, PINE_SERVER, PSVS, SPARKY, TOPSPIN, VNMR, X-PLOR_NIH
**Spectrometer**	BRUKER, VARIAN (850 MHZ, 600 MHZ)

**Protein number**	74
**PDB code**	2KFP, doi:10.2210/pdb2KFP/pdb
**BMRB code**	16186, doi:10.13018/BMR16186
**UniProt code**	Q880Y4, Q880Y4_PSESM
**PDB Header**	STRUCTURAL GENOMICS, UNKNOWN FUNCTION
**Protein name**	PSPTO_3016 FROM PSEUDOMONAS SYRINGAE
**Deposition date**	24.02.2009
**PDB title**	SOLUTION NMR STRUCTURE OF PSPTO_3016 FROM PSEUDOMONAS SYRINGAE. NORTHEAST STRUCTURAL GENOMICS CONSORTIUM TARGET PSR293
**PDB authors**	E.A.FELDMANN, T.A.RAMELOT, L.ZHAO, K.HAMILTON, C.CICCOSANTI, R.XIAO, R.NAIR, J.K.EVERETT, G.SWAPNA, T.B.ACTON, B.ROST, G.T.MONTELIONE, M.A.KENNEDY, NORTHEAST STRUCTURAL GENOMICS CONSORTIUM (NESG)
**Last author**	KENNEDY
**Reference**	J.STRUCT.FUNCT.GENOM. 13, 155 (2012), doi:10.1007/S10969-012-9140-8
**Reference authors**	E.A.FELDMANN, J.SEETHARAMAN, T.A.RAMELOT, S.LEW, L.ZHAO, K.HAMILTON, C.CICCOSANTI, R.XIAO, T.B.ACTON, J.K.EVERETT, L.TONG, G.T.MONTELIONE, M.A.KENNEDY

Reference title	SOLUTION NMR AND X-RAY CRYSTAL STRUCTURES OF PSEUDOMONAS SYRINGAE PSPTO_3016 FROM PROTEIN DOMAIN FAMILY PF04237 (DUF419) ADOPT A "DOUBLE WING" DNA BINDING MOTIF
Software listed	AUTOASSIGN, AUTOSTRUCTURE, NMRPIPE, PSVS, SPARKY, TOPSPIN, VNMR, X-PLOR
Spectrometer	BRUKER, VARIAN (850 MHZ, 600 MHZ)

Protein number	75
PDB code	1SE9, doi:10.2210/pdb1SE9/pdb
BMRB code	6128, doi:10.13018/BMR6128
UniProt code	Q9MAB9, Y3105_ARATH
PDB Header	PLANT PROTEIN
Protein name	AT3G01050, A UBIQUITIN-FOLD PROTEIN FROM ARABIDOPSIS THALIANA
Deposition date	16.02.2004
PDB title	STRUCTURE OF AT3G01050, A UBIQUITIN-FOLD PROTEIN FROM ARABIDOPSIS THALIANA
PDB authors	B.F.VOLKMAN, B.L.LYTLE, F.C.PETERSON, CENTER FOR EUKARYOTIC STRUCTURAL GENOMICS (CESG)
Last author	PETERSON
Reference	NAT.METHODS 1, 149 (2004), doi:10.1038/NMETH716
Reference authors	D.A.VINAROV, B.L.LYTLE, F.C.PETERSON, E.M.TYLER, B.F.VOLKMAN, J.L.MARKLEY
Reference title	CELL-FREE PROTEIN PRODUCTION AND LABELING PROTOCOL FOR NMR-BASED STRUCTURAL PROTEOMICS
Software listed	CYANA, NMRPIPE, SPSCAN, X-PLOR_NIH, XEASY, XWINNMR
Spectrometer	BRUKER (600 MHZ)

Protein number	76
PDB code	2L3G, doi:10.2210/pdb2L3G/pdb
BMRB code	17192, doi:10.13018/BMR17192
UniProt code	Q14155, ARHG7_HUMAN
PDB Header	SIGNALING PROTEIN
Protein name	CH DOMAIN OF RHO GUANINE NUCLEOTIDE EXCHANGE FACTOR 7 FROM HOMO SAPIENS
Deposition date	13.09.2010
PDB title	SOLUTION NMR STRUCTURE OF CH DOMAIN OF RHO GUANINE NUCLEOTIDE EXCHANGE FACTOR 7 FROM HOMO SAPIENS, NORTHEAST STRUCTURAL GENOMICS CONSORTIUM TARGET HR4495E
PDB authors	G.LIU, R.XIAO, H.JANJUA, T.B.ACTON, A.CICCOSANTI, R.SHASTRY, J.EVERETT, G.T.MONTELIONE, NORTHEAST STRUCTURAL GENOMICS CONSORTIUM (NESG)
Last author	MONTELIONE
Reference	
Reference authors	
Reference title	
Software listed	AUTOASSIGN, AUTOSTRUCTURE, CNS, CYANA, NMRPIPE, TALOS+, TOPSPIN, VNMRJ, XEASY
Spectrometer	BRUKER, VARIAN (800 MHZ, 600 MHZ)

Protein number	77
PDB code	2L3B, doi:10.2210/pdb2L3B/pdb
BMRB code	17176, doi:10.13018/BMR17176
UniProt code	Q8ABM6, Q8ABM6_BACTN
PDB Header	STRUCTURAL GENOMICS, UNKNOWN FUNCTION
Protein name	BT_0084 LIPOPROTEIN FROM BACTEROIDES THETAIOTAOMICRON
Deposition date	10.09.2010
PDB title	SOLUTION NMR STRUCTURE OF THE BT_0084 LIPOPROTEIN FROM BACTEROIDES THETAIOTAOMICRON, NORTHEAST STRUCTURAL GENOMICS CONSORTIUM TARGET BTR376
PDB authors	T.A.RAMELOT, Y.YANG, D.WANG, C.CICCOSANTI, H.JANJUA, T.B.ACTON, R.XIAO, J.K.EVERETT, G.T.MONTELIONE, M.A.KENNEDY, NORTHEAST STRUCTURAL GENOMICS CONSORTIUM (NESG)
Last author	KENNEDY
Reference	PROTEINS 80, 667 (2012), doi:10.1002/PROT.23235
Reference authors	T.A.RAMELOT, Y.YANG, R.XIAO, T.B.ACTON, J.K.EVERETT, G.T.MONTELIONE, M.A.KENNEDY
Reference title	SOLUTION NMR STRUCTURE OF BT_0084, A CONJUGATIVE TRANSPOSON LIPOPROTEIN FROM BACTEROIDES THETAIOTAMICRON
Software listed	AUTOASSIGN, AUTOSTRUCTURE, CYANA, NMRPIPE, PDBSTAT, PSVS, SPARKY, TOPSPIN, VNMR, X-PLOR
Spectrometer	BRUKER, VARIAN (850 MHZ, 600 MHZ)

Protein number	7
PDB code	2LRH, doi:10.2210/pdb2LRH/pdb
BMRB code	18372, doi:10.13018/BMR18372
UniProt code	
PDB Header	DE NOVO PROTEIN
Protein name	DE NOVO DESIGNED PROTEIN, P-LOOP NTPASE FOLD
Deposition date	30.03.2012
PDB title	SOLUTION NMR STRUCTURE OF DE NOVO DESIGNED PROTEIN, P-LOOP NTPASE FOLD, NORTHEAST STRUCTURAL GENOMICS CONSORTIUM TARGET OR137
PDB authors	G.LIU, N.KOGA, R.KOGA, R.XIAO, H.LEE, H.JANJUA, E.KOHAN, T.B.ACTON, J.K.EVERETT, D.BAKER, G.T.MONTELIONE, NORTHEAST STRUCTURAL GENOMICS CONSORTIUM (NESG)
Last author	MONTELIONE
Reference	
Reference authors	

**Reference title**	
**Software listed**	AUTOASSIGN, AUTOSTRUCTURE, CNS, CYANA, NMRPIPE, PALES, PSVS, REDCAT, SPARKY, TALOS+, TOPSPIN, VNMRJ, XEASY
**Spectrometer**	BRUKER, VARIAN (800 MHZ, 600 MHZ)

**Protein number**	79
**PDB code**	1VEE, doi:10.2210/pdb1VEE/pdb
**BMRB code**	5929, doi:10.13018/BMR5929
**UniProt code**	Q9M158, Y4105_ARATH
**PDB Header**	STRUCTURAL GENOMICS, UNKNOWN FUNCTION
**Protein name**	HYPOTHETICAL RHODANESE DOMAIN AT4G01050 FROM ARABIDOPSIS THALIANA
**Deposition date**	30.03.2004
**PDB title**	NMR STRUCTURE OF THE HYPOTHETICAL RHODANESE DOMAIN AT4G01050 FROM ARABIDOPSIS THALIANA
**PDB authors**	D.PANTOJA-UCEDA, B.LOPEZ-MENDEZ, S.KOSHIBA, M.INOUE, T.KIGAWA, T.TERADA, M.SHIROUZU, A.TANAKA, M.SEKI, K.SHINOZAKI, S.YOKOYAMA, P.GUNTERT, RIKEN STRUCTURAL GENOMICS/PROTEOMICS INITIATIVE (RSGI)
**Last author**	GUNTERT
**Reference**	PROTEIN SCI. 14, 224 (2005), doi:10.1110/PS.041138705
**Reference authors**	D.PANTOJA-UCEDA, B.LOPEZ-MENDEZ, S.KOSHIBA, M.INOUE, T.KIGAWA, T.TERADA, M.SHIROUZU, A.TANAKA, M.SEKI, K.SHINOZAKI, S.YOKOYAMA, P.GUNTERT
**Reference title**	SOLUTION STRUCTURE OF THE RHODANESE HOMOLOGY DOMAIN AT4G01050(175-295) FROM ARABIDOPSIS THALIANA
**Software listed**	CYANA, OPALP
**Spectrometer**	BRUKER (800 MHZ, 600 MHZ)

**Protein number**	80
**PDB code**	2K1G, doi:10.2210/pdb2K1G/pdb
**BMRB code**	15603, doi:10.13018/BMR15603
**UniProt code**	P0AFV4, SPR_ECOLI
**PDB Header**	LIPOPROTEIN
**Protein name**	LIPOPROTEIN SPR FROM ESCHERICHIA COLI K12
**Deposition date**	03.03.2008
**PDB title**	SOLUTION NMR STRUCTURE OF LIPOPROTEIN SPR FROM ESCHERICHIA COLI K12. NORTHEAST STRUCTURAL GENOMICS TARGET ER541-37-162
**PDB authors**	J.M.ARAMINI, P.ROSSI, L.ZHAO, M.JIANG, M.MAGLAQUI, R.XIAO, J.LIU, M.C.BARAN, G.V.T.SWAPNA, Y.J.HUANG, T.B.ACTON, B.ROST, G.T.MONTELIONE, NORTHEAST STRUCTURAL GENOMICS CONSORTIUM (NESG)
**Last author**	MONTELIONE
**Reference**	BIOCHEMISTRY 47, 9715 (2008), doi:10.1021/BI8010779
**Reference authors**	J.M.ARAMINI, P.ROSSI, Y.J.HUANG, L.ZHAO, M.JIANG, M.MAGLAQUI, R.XIAO, J.LOCKE, R.NAIR, B.ROST, T.B.ACTON, M.INOUYE, G.T.MONTELIONE
**Reference title**	SOLUTION NMR STRUCTURE OF THE NLPC/P60 DOMAIN OF LIPOPROTEIN SPR FROM ESCHERICHIA COLI: STRUCTURAL EVIDENCE FOR A NOVEL CYSTEINE PEPTIDASE CATALYTIC TRIAD
**Software listed**	AUTOASSIGN, AUTOSTRUCTURE, CNS, CYANA, NMRPIPE, PDBSTAT, PSVS, SPARKY, TOPSPIN, VNMR
**Spectrometer**	BRUKER, VARIAN (800 MHZ, 600 MHZ)

**Protein number**	81
**PDB code**	2KKZ, doi:10.2210/pdb2KKZ/pdb
**BMRB code**	16376, doi:10.13018/BMR16376
**UniProt code**	Q6XSU2, Q6XSU2_9INFA
**PDB Header**	ANTIVIRAL PROTEIN
**Protein name**	MONOMERIC W187R MUTANT OF A/UDORN NS1 EFFECTOR DOMAIN
**Deposition date**	29.06.2009
**PDB title**	SOLUTION NMR STRUCTURE OF THE MONOMERIC W187R MUTANT OF A/UDORN NS1 EFFECTOR DOMAIN. NORTHEAST STRUCTURAL GENOMICS TARGET OR8C[W187R]
**PDB authors**	J.M.ARAMINI, L.MA, H.LEE, L.ZHAO, K.CUNNINGHAM, C.CICCOSANTI, H.JANJUA, Y.FANG, R.XIAO, R.M.KRUG, G.T.MONTELIONE, NORTHEAST STRUCTURAL GENOMICS CONSORTIUM (NESG)
**Last author**	MONTELIONE
**Reference**	
**Reference authors**	
**Reference title**	
**Software listed**	AUTOASSIGN, AUTOSTRUCTURE, CNS, CYANA, MOLPROBITY, NMRPIPE, PDBSTAT, PSVS, SPARKY, TALOS, TOPSPIN
**Spectrometer**	BRUKER (800 MHZ, 600 MHZ)

**Protein number**	82
**PDB code**	1VDY, doi:10.2210/pdb1VDY/pdb
**BMRB code**	5928, doi:10.13018/BMR5928
**UniProt code**	Q9C5H4, Q9C5H4_ARATH
**PDB Header**	STRUCTURAL GENOMICS, UNKNOWN FUNCTION
**Protein name**	HYPOTHETICAL ENTH-VHS DOMAIN AT3G16270 FROM ARABIDOPSIS THALIANA
**Deposition date**	25.03.2004
**PDB title**	NMR STRUCTURE OF THE HYPOTHETICAL ENTH-VHS DOMAIN AT3G16270 FROM ARABIDOPSIS THALIANA

**PDB authors**	B.LOPEZ-MENDEZ, D.PANTOJA-UCEDA, T.TOMIZAWA, S.KOSHIBA, T.KIGAWA, M.SHIROUZU, T.TERADA, M.INOUE, T.YABUKI, M.AOKI, E.SEKI, T.MATSUDA, H.HIROTA, M.YOSHIDA, A.TANAKA, T.OSANAI, M.SEKI, K.SHINOZAKI, S.YOKOYAMA, P.GUNTERT, RIKEN STRUCTURAL GENOMICS/PROTEOMICS INITIATIVE (RSGI)
**Last author**	GUNTERT
**Reference**	
**Reference authors**	
**Reference title**	
**Software listed**	CYANA, OPALP
**Spectrometer**	BRUKER (800 MHZ, 600 MHZ)

**Protein number**	83
**PDB code**	2KKL, doi:10.2210/pdb2KKL/pdb
**BMRB code**	16364, doi:10.13018/BMR16364
**UniProt code**	P64898, Y1858_MYCBO
**PDB Header**	STRUCTURAL GENOMICS, UNKNOWN FUNCTION
**Protein name**	FHA DOMAIN OF MB1858 FROM MYCOBACTERIUM BOVIS
**Deposition date**	25.06.2009
**PDB title**	SOLUTION NMR STRUCTURE OF FHA DOMAIN OF MB1858 FROM MYCOBACTERIUM BOVIS. NORTHEAST STRUCTURAL GENOMICS CONSORTIUM TARGET MBR243C (24- 155)
**PDB authors**	Y.YANG, T.A.RAMELOT, D.WANG, E.L.FOOTE, M.JIANG, R.NAIR, B.ROST, G.SWAPNA, T.B.ACTON, R.XIAO, J.K.EVERETT, G.T.MONTELIONE, M.A.KENNEDY, NORTHEAST STRUCTURAL GENOMICS CONSORTIUM (NESG)
**Last author**	KENNEDY
**Reference**	
**Reference authors**	
**Reference title**	
**Software listed**	AUTOASSIGN, AUTOSTRUCTURE, CNS, NMRPIPE, PDBSTAT, PSVS, SPARKY, TOPSPIN, VNMR, X-PLOR
**Spectrometer**	BRUKER, VARIAN (850 MHZ, 600 MHZ)

**Protein number**	84
**PDB code**	2N4B, doi:10.2210/pdb2N4B/pdb
**BMRB code**	17611, doi:10.13018/BMR17611
**UniProt code**	Q1LD49, Q1LD49_RALME
**PDB Header**	STRUCTURAL GENOMICS, UNKNOWN FUNCTION
**Protein name**	RALSTONIA METALLIDURANS RMET_5065 DETERMINED BY COMBINING EVOLUTIONARY COUPLINGS (EC) AND SPARSE NMR DATA
**Deposition date**	17.06.2015
**PDB title**	EC-NMR STRUCTURE OF RALSTONIA METALLIDURANS RMET_5065 DETERMINED BY COMBINING EVOLUTIONARY COUPLINGS (EC) AND SPARSE NMR DATA. NORTHEAST STRUCTURAL GENOMICS CONSORTIUM TARGET CRR115
**PDB authors**	Y.TANG, Y.J.HUANG, T.A.HOPF, C.SANDER, D.MARKS, G.T.MONTELIONE, NORTHEAST STRUCTURAL GENOMICS CONSORTIUM (NESG)
**Last author**	MONTELIONE
**Reference**	NAT.METHODS 12, 751 (2015), doi:10.1038/NMETH.3455
**Reference authors**	Y.TANG, Y.J.HUANG, T.A.HOPF, C.SANDER, D.S.MARKS, G.T.MONTELIONE
**Reference title**	PROTEIN STRUCTURE DETERMINATION BY COMBINING SPARSE NMR DATA WITH EVOLUTIONARY COUPLINGS
**Software listed**	ASDP, CYANA, EC-NMR, EVFOLD-PLM, REDUCE, ROSETTA, TALOS+
**Spectrometer**	

**Protein number**	85
**PDB code**	2L8V, doi:10.2210/pdb2L8V/pdb
**BMRB code**	17429, doi:10.13018/BMR17429
**UniProt code**	P50034, PYR1_THEEB
**PDB Header**	PHOTOSYNTHESIS
**Protein name**	PHYCOBILISOME LINKER POLYPEPTIDE DOMAIN OF CPCC (20-153) FROM THERMOSYNECHOCOCCUS ELONGATUS
**Deposition date**	26.01.2011
**PDB title**	SOLUTION NMR STRUCTURE OF THE PHYCOBILISOME LINKER POLYPEPTIDE DOMAIN OF CPCC (20-153) FROM THERMOSYNECHOCOCCUS ELONGATUS, NORTHEAST STRUCTURAL GENOMICS CONSORTIUM TARGET TER219A
**PDB authors**	T.A.RAMELOT, Y.YANG, J.R.CORT, D.LEE, C.CICCOSANTI, K.HAMILTON, T.B.ACTON, R.XIAO, J.K.EVERETT, G.T.MONTELIONE, M.A.KENNEDY, NORTHEAST STRUCTURAL GENOMICS CONSORTIUM (NESG)
**Last author**	KENNEDY
**Reference**	
**Reference authors**	
**Reference title**	
**Software listed**	AUTOASSIGN, AUTOSTRUCTURE, CNS, CYANA, NMRPIPE, PDBSTAT, PINE_SERVER, PSVS, SPARKY, TOPSPIN, VNMR, X-PLOR_NIH
**Spectrometer**	BRUKER, VARIAN (850 MHZ, 600 MHZ)

**Protein number**	86
**PDB code**	2LGH, doi:10.2210/pdb2LGH/pdb
**BMRB code**	17809, doi:10.13018/BMR17809
**UniProt code**	A0KKT0, A0KKT0_AERHH
**PDB Header**	STRUCTURAL GENOMICS, UNKNOWN FUNCTION
**Protein name**	AHSA1-LIKE PROTEIN AHA_2358 FROM AEROMONAS HYDROPHILA REFINED WITH NH RDCS

**Deposition date**	26.07.2011
**PDB title**	SOLUTION NMR STRUCTURE OF THE AHSA1-LIKE PROTEIN AHA_2358 FROM AEROMONAS HYDROPHILA REFINED WITH NH RDCS, NORTHEAST STRUCTURAL GENOMICS CONSORTIUM TARGET AHR99
**PDB authors**	T.A.RAMELOT, Y.YANG, H.LEE, D.WANG, C.CICCOSANTI, H.JANJUA, R.NAIR, T.B.ACTON, R.XIAO, J.K.EVERETT, J.H.PRESTEGARD, G.T.MONTELIONE, M.A.KENNEDY, NORTHEAST STRUCTURAL GENOMICS CONSORTIUM (NESG)
**Last author**	KENNEDY
**Reference**	
**Reference authors**	
**Reference title**	
**Software listed**	AUTOASSIGN, AUTOSTRUCTURE, CNS, CYANA, NMRPIPE, PDBSTAT, PINE_SERVER, PSVS, SPARKY, TOPSPIN, VNMR, X-PLOR_NIH
**Spectrometer**	BRUKER, VARIAN (850 MHZ, 600 MHZ)

**Protein number**	87
**PDB code**	2K1S, doi:10.2210/pdb2K1S/pdb
**BMRB code**	15683, doi:10.13018/BMR15683
**UniProt code**	P37665, YIAD_ECOLI
**PDB Header**	LIPOPROTEIN
**Protein name**	FOLDED C-TERMINAL FRAGMENT OF YIAD FROM ESCHERICHIA COLI
**Deposition date**	14.03.2008
**PDB title**	SOLUTION NMR STRUCTURE OF THE FOLDED C-TERMINAL FRAGMENT OF YIAD FROM ESCHERICHIA COLI. NORTHEAST STRUCTURAL GENOMICS CONSORTIUM TARGET ER553
**PDB authors**	T.A.RAMELOT, L.ZHAO, K.HAMILTON, M.MAGLAQUI, R.XIAO, J.LIU, M.C.BARAN, G.SWAPNA, T.B.ACTON, B.ROST, G.T.MONTELIONE, M.A.KENNEDY, NORTHEAST STRUCTURAL GENOMICS CONSORTIUM (NESG)
**Last author**	KENNEDY
**Reference**	
**Reference authors**	
**Reference title**	
**Software listed**	AUTOASSIGN, AUTOSTRUCTURE, CNS, NMRPIPE, PSVS, SPARKY, TOPSPIN, VNMR, X-PLOR
**Spectrometer**	BRUKER, VARIAN (850 MHZ, 600 MHZ)

**Protein number**	88
**PDB code**	2M4F, doi:10.2210/pdb2M4F/pdb
**BMRB code**	19001, doi:10.13018/BMR19001
**UniProt code**	E4QGX1, E4QGX1_BORBN
**PDB Header**	IMMUNE SYSTEM
**Protein name**	OUTER SURFACE PROTEIN E
**Deposition date**	05.02.2013
**PDB title**	SOLUTION STRUCTURE OF OUTER SURFACE PROTEIN E
**PDB authors**	A.BHATTACHARJEE, J.S.OEEMIG, R.KOLODZIEJCZYK, T.MERI, T.KAJANDER, H.IWAI, T.JOKIRANTA, A.GOLDMAN
**Last author**	GOLDMAN
**Reference**	J.BIOL.CHEM. 288, 18685 (2013), doi:10.1074/JBC.M113.459040
**Reference authors**	A.BHATTACHARJEE, J.S.OEEMIG, R.KOLODZIEJCZYK, T.MERI, T.KAJANDER, M.J.LEHTINEN, H.IWAI, T.S.JOKIRANTA, A.GOLDMAN
**Reference title**	STRUCTURAL BASIS FOR COMPLEMENT EVASION BY LYME DISEASE PATHOGEN BORRELIA BURGDORFERI
**Software listed**	AMBER, CCPNMR_ANALYSIS, CING, CYANA, NMRPIPE, VNMRJ
**Spectrometer**	VARIAN (800 MHZ, 600 MHZ)

**Protein number**	89
**PDB code**	2JXP, doi:10.2210/pdb2JXP/pdb
**BMRB code**	15568, doi:10.13018/BMR15568
**UniProt code**	Q82VF2, Q82VF2_NITEU
**PDB Header**	LIPOPROTEIN
**Protein name**	UNCHARACTERIZED LIPOPROTEIN B FROM NITROSOMONAS EUROPAEA
**Deposition date**	27.11.2007
**PDB title**	SOLUTION NMR STRUCTURE OF UNCHARACTERIZED LIPOPROTEIN B FROM NITROSOMONAS EUROPAEA. NORTHEAST STRUCTURAL GENOMICS TARGET NER45A
**PDB authors**	P.ROSSI, D.WANG, H.JANJUA, L.OWENS, R.XIAO, M.C.BARAN, G.SWAPNA, T.B.ACTON, B.ROST, G.T.MONTELIONE, NORTHEAST STRUCTURAL GENOMICS CONSORTIUM (NESG)
**Last author**	MONTELIONE
**Reference**	
**Reference authors**	
**Reference title**	
**Software listed**	AUTOASSIGN, CNS, CYANA, MOLMOL, MOLPROBITY, NMRPIPE, PROCHECK, PSVS, RPF, SPARKY, TOPSPIN, XEASY
**Spectrometer**	BRUKER (800 MHZ)

**Protein number**	90
**PDB code**	2L06, doi:10.2210/pdb2L06/pdb
**BMRB code**	17031, doi:10.13018/BMR17031
**UniProt code**	Q55544, Q55544_SYNY3
**PDB Header**	PROTEIN BINDING

**Protein name**	PBS LINKER POLYPEPTIDE DOMAIN (FRAGMENT 254-400) OF PHYCOBILISOME LINKER PROTEIN APCE FROM SYNECHOCYSTIS SP. PCC 6803
**Deposition date**	30.06.2010
**PDB title**	SOLUTION NMR STRUCTURE OF THE PBS LINKER POLYPEPTIDE DOMAIN (FRAGMENT 254-400) OF PHYCOBILISOME LINKER PROTEIN APCE FROM SYNECHOCYSTIS SP. PCC 6803. NORTHEAST STRUCTURAL GENOMICS CONSORTIUM TARGET SGR209C
**PDB authors**	T.A.RAMELOT, Y.YANG, J.R.CORT, K.HAMILTON, C.CICCOSANTI, D.LEE, T.B.ACTON, R.XIAO, J.K.EVERETT, G.T.MONTELIONE, M.A.KENNEDY, NORTHEAST STRUCTURAL GENOMICS CONSORTIUM (NESG)
**Last author**	KENNEDY
**Reference**	
**Reference authors**	
**Reference title**	
**Software listed**	AUTOASSIGN, AUTOSTRUCTURE, CYANA, NMRPIPE, PDBSTAT, PSVS, SPARKY, TOPSPIN, VNMR, X-PLOR
**Spectrometer**	BRUKER, VARIAN (850 MHZ, 750 MHZ, 600 MHZ, 500 MHZ)

**Protein number**	91
**PDB code**	2LAH, doi:10.2210/pdb2LAH/pdb
**BMRB code**	17524, doi:10.13018/BMR17524
**UniProt code**	O43683, BUB1_HUMAN
**PDB Header**	CELL CYCLE, APOPTOSIS
**Protein name**	MITOTIC CHECKPOINT SERINE/THREONINE-PROTEIN KINASE BUB1 N-TERMINAL DOMAIN FROM HOMO SAPIENS
**Deposition date**	14.03.2011
**PDB title**	SOLUTION NMR STRUCTURE OF MITOTIC CHECKPOINT SERINE/THREONINE-PROTEIN KINASE BUB1 N-TERMINAL DOMAIN FROM HOMO SAPIENS, NORTHEAST STRUCTURAL GENOMICS CONSORTIUM TARGET HR5460A (METHODS DEVELOPMENT)
**PDB authors**	G.LIU, R.XIAO, H.LEE, K.HAMILTON, T.B.ACTON, C.CICCOSANTI, J.K.EVERETT, R.T.SHASTRY, Y.J.HUANG, G.T.MONTELIONE, N.NORTHEAST STRUCTURAL GENOMICS CONSORTIUM, NORTHEAST STRUCTURAL GENOMICS CONSORTIUM (NESG)
**Last author**	MONTELIONE
**Reference**	
**Reference authors**	
**Reference title**	
**Software listed**	AUTOASSIGN, AUTOSTRUCTURE, CNS, CYANA, NMRPIPE, SPARKY, TALOS+, TOPSPIN, VNMRJ, XEASY
**Spectrometer**	BRUKER, VARIAN (800 MHZ, 600 MHZ)

**Protein number**	92
**PDB code**	2LAK, doi:10.2210/pdb2LAK/pdb
**BMRB code**	17530, doi:10.13018/BMR17530
**UniProt code**	Q2K6S8, Q2K6S8_RHIEC
**PDB Header**	STRUCTURE GENOMICS, UNKNOWN FUNCTION
**Protein name**	AHSA1-LIKE PROTEIN RHE_CH02687 (1-152) FROM RHIZOBIUM ETLI
**Deposition date**	16.03.2011
**PDB title**	SOLUTION NMR STRUCTURE OF THE AHSA1-LIKE PROTEIN RHE_CH02687 (1-152) FROM RHIZOBIUM ETLI, NORTHEAST STRUCTURAL GENOMICS CONSORTIUM TARGET RER242
**PDB authors**	Y.YANG, T.A.RAMELOT, J.R.CORT, D.WANG, C.CICCOSANTI, H.JANJUA, R.NAIR, B.ROST, T.B.ACTON, R.XIAO, J.K.EVERETT, G.T.MONTELIONE, M.A.KENNEDY, NORTHEAST STRUCTURAL GENOMICS CONSORTIUM (NESG)
**Last author**	KENNEDY
**Reference**	
**Reference authors**	
**Reference title**	
**Software listed**	AUTOASSIGN, AUTOSTRUCTURE, CNS, CYANA, NMRPIPE, PDBSTAT, PINE_SERVER, PSVS, SPARKY, TOPSPIN, VNMR, X-PLOR_NIH
**Spectrometer**	BRUKER, VARIAN (850 MHZ, 600 MHZ)

**Protein number**	93
**PDB code**	2L82, doi:10.2210/pdb2L82/pdb
**BMRB code**	17390, doi:10.13018/BMR17390
**UniProt code**	
**PDB Header**	DE NOVO PROTEIN
**Protein name**	DE NOVO DESIGNED PROTEIN, P-LOOP NTPASE FOLD
**Deposition date**	31.12.2010
**PDB title**	SOLUTION NMR STRUCTURE OF DE NOVO DESIGNED PROTEIN, P-LOOP NTPASE FOLD, NORTHEAST STRUCTURAL GENOMICS CONSORTIUM TARGET OR32
**PDB authors**	G.LIU, N.KOGA, R.KOGA, R.XIAO, K.HAMILTON, H.JANJUA, S.TONG, T.B.ACTON, J.EVERETT, D.BAKER, G.T.MONTELIONE, NORTHEAST STRUCTURAL GENOMICS CONSORTIUM (NESG)
**Last author**	MONTELIONE
**Reference**	
**Reference authors**	
**Reference title**	
**Software listed**	AUTOASSIGN, AUTOSTRUCTURE, CNS, CYANA, NMRPIPE, PALES, PINE, REDCAT, SPARKY, TALOS+, TOPSPIN, VNMRJ, XEASY
**Spectrometer**	BRUKER, VARIAN (800 MHZ, 600 MHZ)

**Protein number**	94
**PDB code**	2M47, doi:10.2210/pdb2M47/pdb
**BMRB code**	18989, doi:10.13018/BMR18989
**UniProt code**	Q8NN40, Q8NN40_CORGL
**PDB Header**	STRUCTURAL GENOMICS, UNKNOWN FUNCTION
**Protein name**	POLYKETIDE_CYC-LIKE PROTEIN CGL2372 FROM CORYNEBACTERIUM GLUTAMICUM
**Deposition date**	30.01.2013
**PDB title**	SOLUTION NMR STRUCTURE OF THE POLYKETIDE_CYC-LIKE PROTEIN CGL2372 FROM CORYNEBACTERIUM GLUTAMICUM, NORTHEAST STRUCTURAL GENOMICS CONSORTIUM TARGET CGR160
**PDB authors**	Y.YANG, T.A.RAMELOT, D.LEE, C.CICCOSANTI, A.SAPIN, H.JANJUA, R.NAIR, B.ROST, T.B.ACTON, R.XIAO, J.K.EVERETT, G.T.MONTELIONE, M.A.KENNEDY, NORTHEAST STRUCTURAL GENOMICS CONSORTIUM (NESG)
**Last author**	KENNEDY
**Reference**	
**Reference authors**	
**Reference title**	
**Software listed**	AUTOASSIGN, AUTOSTRUCTURE, CNS, CYANA, NMRPIPE, PINE, PSVS, SPARKY, TALOS+, TOPSPIN, VNMRJ, XEASY
**Spectrometer**	BRUKER, VARIAN (850 MHZ, 600 MHZ)

**Protein number**	95
**PDB code**	2K3A, doi:10.2210/pdb2K3A/pdb
**BMRB code**	15335, doi:10.13018/BMR15335
**UniProt code**	Q49ZM2, Q49ZM2_STAS1
**PDB Header**	HYDROLASE
**Protein name**	STAPHYLOCOCCUS SAPROPHYTICUS CHAP (CYSTEINE, HISTIDINE-DEPENDENT AMIDOHYDROLASES/PEPTIDASES) DOMAIN PROTEIN
**Deposition date**	29.04.2008
**PDB title**	NMR SOLUTION STRUCTURE OF STAPHYLOCOCCUS SAPROPHYTICUS CHAP (CYSTEINE, HISTIDINE-DEPENDENT AMIDOHYDROLASES/PEPTIDASES) DOMAIN PROTEIN. NORTHEAST STRUCTURAL GENOMICS CONSORTIUM TARGET SYR11
**PDB authors**	P.ROSSI, J.M.ARAMINI, C.X.CHEN, C.NWOSU, K.C.CUNNINGHAM, L.A.OWENS, R.XIAO, J.LIU, M.C.BARAN, G.SWAPNA, T.B.ACTON, B.ROST, G.T.MONTELIONE, NORTHEAST STRUCTURAL GENOMICS CONSORTIUM (NESG)
**Last author**	MONTELIONE
**Reference**	PROTEINS 74, 515 (2008), doi:10.1002/PROT.22267
**Reference authors**	P.ROSSI, J.M.ARAMINI, R.XIAO, C.X.CHEN, C.NWOSU, L.A.OWENS, M.MAGLAQUI, R.NAIR, M.FISCHER, T.B.ACTON, B.HONIG, B.ROST, G.T.MONTELIONE
**Reference title**	STRUCTURAL ELUCIDATION OF THE CYS-HIS-GLU-ASN PROTEOLYTIC RELAY IN THE SECRETED CHAP DOMAIN ENZYME FROM THE HUMAN PATHOGEN STAPHYLOCOCCUS SAPROPHYTICUS
**Software listed**	AUTOASSIGN, AUTOSTRUCTURE, CNS, CYANA, MOLMOL, MOLPROBITY, NMRPIPE, PDBSTAT, PROCHECK, PROSA, PSVS, SPARKY, TOPSPIN, VERIFY3D, X-PLOR
**Spectrometer**	BRUKER (800 MHZ, 600 MHZ)

**Protein number**	96
**PDB code**	2M7U, doi:10.2210/pdb2M7U/pdb
**BMRB code**	19213, doi:10.13018/BMR19213
**UniProt code**	Q8DLC7, Q8DLC7_THEEB
**PDB Header**	SIGNALING PROTEIN
**Protein name**	BLUE LIGHT-ABSORBING STATE OF TEPIXJ, AN ACTIVE CYANOBACTERIOCHROME DOMAIN
**Deposition date**	01.05.2013
**PDB title**	BLUE LIGHT-ABSORBING STATE OF TEPIXJ, AN ACTIVE CYANOBACTERIOCHROME DOMAIN
**PDB authors**	G.CORNILESCU, C.C.CORNILESCU, S.E.BURGIE, J.M.WALKER, J.L.MARKLEY, A.T.ULIJASZ, R.D.VIERSTRA
**Last author**	VIERSTRA
**Reference**	
**Reference authors**	
**Reference title**	
**Software listed**	NMRPIPE, PIPP, X-PLOR_NIH
**Spectrometer**	BRUKER, VARIAN (900 MHZ, 800 MHZ, 700 MHZ, 600 MHZ)

**Protein number**	97
**PDB code**	2B3W, doi:10.2210/pdb2B3W/pdb
**BMRB code**	6782, doi:10.13018/BMR6782
**UniProt code**	P30176, YBIA_ECOLI
**PDB Header**	STRUCTURAL GENOMICS, UNKNOWN FUNCTION
**Protein name**	E.COLI PROTEIN YBIA
**Deposition date**	21.09.2005
**PDB title**	NMR STRUCTURE OF THE E.COLI PROTEIN YBIA, NORTHEAST STRUCTURAL GENOMICS TARGET ET24
**PDB authors**	T.A.RAMELOT, J.R.CORT, R.XIAO, L.Y.SHIH, T.B.ACTON, G.T.MONTELIONE, M.A.KENNEDY, NORTHEAST STRUCTURAL GENOMICS CONSORTIUM (NESG)
**Last author**	KENNEDY
**Reference**	
**Reference authors**	
**Reference title**	

**Software listed**	AUTOSTRUCTURE, CNS, NMRPIPE, SPARKY, VNMR, X-PLOR
**Spectrometer**	VARIAN (800 MHZ, 750 MHZ, 600 MHZ)

**Protein number**	98
**PDB code**	(KRAS4B)
**BMRB code**	
**PDB Header**	
**Protein name**	(KRAS)
**Deposition date**	
**PDB title**	
**PDB authors**	(RIEK)
**Last author**	
**Reference**	
**Reference authors**	
**Reference title**	
**Software listed**	
**Spectrometer**	(BRUKER (700 MHZ))

**Protein number**	99
**PDB code**	2G0Q, doi:10.2210/pdb2G0Q/pdb
**BMRB code**	7007, doi:10.13018/BMR7007
**UniProt code**	
**PDB Header**	STRUCTURAL GENOMICS, UNKNOWN FUNCTION
**Protein name**	AT5G39720.1 FROM ARABIDOPSIS THALIANA
**Deposition date**	13.02.2006
**PDB title**	SOLUTION STRUCTURE OF AT5G39720.1 FROM ARABIDOPSIS THALIANA
**PDB authors**	B.F.VOLKMAN, F.C.PETERSON, B.L.LYTLE, CENTER FOR EUKARYOTIC STRUCTURAL GENOMICS (CESG)
**Last author**	LYTLE
**Reference**	ACTA CRYSTALLOGR.,SECT.F 62, 490 (2006), doi:10.1107/S1744309106015946
**Reference authors**	B.L.LYTLE, F.C.PETERSON, E.M.TYLER, C.L.NEWMAN, D.A.VINAROV, J.L.MARKLEY, B.F.VOLKMAN
**Reference title**	SOLUTION STRUCTURE OF ARABIDOPSIS THALIANA PROTEIN AT5G39720.1, A MEMBER OF THE AIG2-LIKE PROTEIN FAMILY
**Software listed**	GARANT, NMRPIPE, SPSCAN, X-PLOR_NIH, XEASY, XWINNMR
**Spectrometer**	BRUKER (600 MHZ)

**Protein number**	100
**PDB code**	2LF2, doi:10.2210/pdb2LF2/pdb
**BMRB code**	17736, doi:10.13018/BMR17736
**UniProt code**	Q11W30, Q11W30_CYTH3
**PDB Header**	STRUCTURAL GENOMICS, UNKNOWN FUNCTION
**Protein name**	AHSA1-LIKE PROTEIN CHU_1110 FROM CYTOPHAGA HUTCHINSONII
**Deposition date**	28.06.2011
**PDB title**	SOLUTION NMR STRUCTURE OF THE AHSA1-LIKE PROTEIN CHU_1110 FROM CYTOPHAGA HUTCHINSONII, NORTHEAST STRUCTURAL GENOMICS CONSORTIUM TARGET CHR152
**PDB authors**	Y.YANG, T.A.RAMELOT, D.LEE, C.CICCOSANTI, T.B.ACTON, R.XIAO, J.K.EVERETT, G.T.MONTELIONE, M.A.KENNEDY, NORTHEAST STRUCTURAL GENOMICS CONSORTIUM (NESG)
**Last author**	KENNEDY
**Reference**	
**Reference authors**	
**Reference title**	
**Software listed**	AUTOASSIGN, AUTOSTRUCTURE, CNS, CYANA, NMRPIPE, PDBSTAT, PINE_SERVER, PSVS, SPARKY, TOPSPIN, VNMR, X-PLOR_NIH
**Spectrometer**	BRUKER, VARIAN (850 MHZ, 600 MHZ)

Supplementary Table 3. Spectrum types, axis labels, and expected peaks for the spectra in the 100-protein NMR spectra dataset. As defined in the CYANA/FLYA library.

**Spectrum type**	**Axis labels**	**Expected peaks** (*i*, *j* = residue numbers)
NOESY	H1, H2	$H1_i$ ~ $H2_j$ ($d \le 6$ Å)
TOCSY	H1, H2	$H1_i$ — $H2_i$
C13HSQC	C, H	C_i – H_i
N15HSQC	N, H	N_i – H_i
CBHDaro	C, H	C^{β}_i — H^{δ}_i in aromatic sidechains
C13NOESY	C, HC, H	C_i – HC_i ~ H_j ($d \le 6$ Å)
N15NOESY	N, HN, H	N_i – HN_i ~ H_j ($d \le 6$ Å)
CCNOESY	C1, H1, H2, C2	$C1_i$ – $H1_i$ ~ $H2_j$ – $C2_j$ ($d \le 6$ Å)
HNCA	HN, N, C	HN_i – N_i – C^{α}_i, HN_i – N_i — C^{α}_{i-1}
HNcoCA	HN, N, C	HN_i – N_i — C^{α}_{i-1}
HNCO	HN, N, C	HN_i – N_i – C'_{i-1}
HNcaCO	HN, N, C	HN_i – N_i — C'_i, HN_i – N_i – C'_{i-1}
HNHA	HN, N, H	HN_i – N_i – H^{α}_i, HN_i – N_i — H^{α}_{i-1}
CBCANH	HN, N, C	HN_i – N_i – C^{α}_i, HN_i – N_i — C^{β}_i, HN_i – N_i — C^{α}_{i-1}, HN_i – N_i — C^{β}_{i-1}
CBCAcoNH	HN, N, C	HN_i – N_i — C^{α}_{i-1}, HN_i – N_i — C^{β}_{i-1}
HBHAcoNH	HN, N, H	HN_i – N_i — H^{α}_{i-1}, HN_i – N_i — H^{β}_{i-1}
CBCACOcaHA	C, CO, HA	C^{α}_i – C'_i – H^{α}_i, C^{β}_i – C'_i – H^{α}_i
N15TOCSY	HN, N, H	HN_i – N_i – H^x_i (x = N, α, β, γ)
HCcoNH	HN, N, H	HN_i – N_i — H^x_{i-1} (x = α, β, γ, δ, ε)
CcoNH	HN, N, C	HN_i – N_i — C^x_{i-1} (x = α, β, γ, δ, ε)
HCCHCOSY	H, HC, C	HC_i – C_i — H_i (H_i in same or nearest neighbor CH_n group)
HCCHTOCSY	H, HC, C	HC_i – C_i — H_i (H_i in CH_n group)
CCHTOCSY	H1, C1, C2	$H1_i$ – $C1_i$ — $C2_i$ ($C2_i$ in CH_n group)

Formal magnetization transfer types are indicated by '–' for one-bond scalar coupling transfers, '—' for other through-bond transfers, and '~' for through-space (NOE) transfer. CCNOESY is the 4D HC-HC NOESY experiment.

Supplementary Table 4. Spectra in the 100-protein NMR spectra dataset. The column 'MHz' gives the ^{1}H spectrometer frequency. For each dimension, column 'L*' is the axis label, 'Size' denotes the number of real data points, and 'Range (ppm)' gives the spectral chemical shift range.

PDB	Spectrum name	MHz	L1	Size	Range (ppm)	L2	Size	Range (ppm)	L3	Size	Range (ppm)	L4	Size	Range (ppm)
6SVC	C13NOESY	700	C	128	33.14 62.96	HC	632	-1.02 7.79	H	474	-1.02 11.00			
6SVC	CBCANH	600	N	128	97.42 133.42	C	256	25.06 71.06	HN	1024	4.95 11.43			
6SVC	HNCA	900	N	256	97.55 135.57	C	256	38.45 70.46	HN	1024	4.97 12.96			
6SVC	N15NOESY	700	N	128	96.93 136.08	HN	362	5.95 11.00	H	474	-1.02 11.00			
6SVC	NOESY	900	H1	2048	-1.99 11.91	H2	1917	-1.52 11.49						
6SVC	TOCSY	900	H1	1024	-2.00 11.90	H2	1917	-1.53 11.48						
2JVD	C13HSQC_@ALI	600	C	1024	4.47 70.79	H	1024	-1.85 4.82						
2JVD	C13NOESY	800	C	512	19.75 89.75	HC	652	0.46 7.46	H	373	0.16 9.63			
2JVD	CBCANH	800	N	256	102.61 127.61	C	512	4.21 79.28	HN	512	4.78 10.28			
2JVD	CBCAcoNH	800	N	256	102.61 127.61	C	512	4.27 79.23	HN	512	4.78 10.28			
2JVD	CCHTOCSY_@ALI	800	C1	256	29.75 53.75	C2	512	4.27 79.23	H1	441	-0.02 5.99			
2JVD	HBHAcoNH	800	N	256	102.61 127.61	HN	512	4.79 10.29	H	512	1.04 6.04			
2JVD	HCCHCOSY_@ALI	800	C	256	29.75 53.75	HC	512	0.28 6.28	H	559	-0.01 6.00			
2JVD	HNCO	800	N	256	102.61 127.61	C	512	168.73 188.73	HN	512	4.78 10.28			
2JVD	HNcaCO	800	N	256	102.61 127.61	C	512	168.75 188.75	HN	512	4.79 10.29			
2JVD	N15HSQC	800	N	1024	80.11 140.11	H	1024	4.78 11.76						
2JVD	N15NOESY	800	N	512	104.15 134.95	HN	316	5.93 9.33	H	373	0.16 9.63			
2K57	C13HSQC_@ALI	800	C	1024	5.18 80.14	H	1024	-2.19 11.76						
2K57	C13HSQC_@ARO	800	C	1024	112.75 142.76	H	1024	-2.19 11.76						
2K57	C13NOESY_@ALI	800	C	256	19.75 89.74	HC	544	-1.37 6.04	H	440	-1.03 11.00			
2K57	CBCANH	800	N	256	104.10 134.11	C	512	4.25 79.32	HN	512	4.79 11.76			
2K57	CBCAcoNH	800	N	256	104.10 134.11	C	512	4.12 79.19	HN	512	4.81 11.78			
2K57	CCHTOCSY_@ALI	800	C1	512	29.72 53.71	C2	256	4.18 79.14	H1	551	-1.01 6.50			
2K57	CcoNH	600	N	256	101.57 134.48	C	512	15.40 81.71	HN	512	4.78 11.77			
2K57	HBHAcoNH	800	N	256	104.10 134.11	HN	512	4.78 11.75	H	512	1.03 6.03			
2K57	HCCHCOSY_@ALI	600	C	256	25.55 49.56	HC	512	-0.23 9.77	H	550	-1.01 6.50			
2K57	HCCHTOCSY_@ALI	600	C	256	25.49 49.50	HC	512	-0.22 9.78	H	550	-1.01 6.50			
2K57	HNCO	800	N	256	104.10 134.11	C	512	168.76 188.75	HN	512	4.79 11.76			
2K57	HNcaCO	800	N	256	104.14 134.14	C	512	168.75 188.74	HN	512	4.78 11.75			
2K57	N15HSQC	800	N	1024	80.10 140.11	H	2048	-2.19 11.76						
2K57	N15NOESY	800	N	256	104.19 135.02	HN	474	5.31 11.76	H	440	-1.03 11.00			
6SOW	C13HSQC	850	C	962	3.15 81.04	H	999	-0.07 5.31						
6SOW	C13NOESY_@ALI	850	C	128	10.66 68.69	HC	526	-0.05 5.61	H	860	0.15 9.41			
6SOW	C13NOESY_@ARO	850	C	128	111.76 141.78	HC	455	5.94 8.39	H	875	-0.33 9.10			
6SOW	CBCANH	850	N	128	105.00 131.00	C	256	5.56 85.64	HN	512	4.68 9.93			
6SOW	HCCHCOSY_@ALI	850	C	128	2.62 70.59	HC	128	-2.83 7.17	H	1024	-0.34 9.66			
6SOW	HNCA	850	N	256	104.99 130.99	C	256	40.78 70.80	HN	1024	4.69 9.94			
6SOW	HNCO	850	N	256	105.06 131.05	C	256	168.85 182.85	HN	1024	4.69 9.94			
6SOW	HNcaCO	850	N	256	104.98 130.98	C	256	168.87 182.87	HN	1024	4.68 9.93			
6SOW	N15HSQC	850	N	1024	104.99 134.99	H	1024	4.68 10.20						
6SOW	N15NOESY	850	N	128	104.99 130.98	HN	1024	4.69 10.20	H	1024	-0.82 10.17			
2LX7	C13HSQC_@ALI	850	C	512	3.30 88.30	H	440	-1.21 6.50						
2LX7	C13HSQC_@ARO	850	C	256	109.97 139.97	H	274	5.48 9.50						
2LX7	C13NOESY_@ALI	850	C	256	13.00 79.00	HC	430	-1.52 6.01	H	256	-1.22 10.78			
2LX7	C13NOESY_@ARO	850	C	128	111.30 141.30	HC	274	5.48 9.50	H	256	-1.24 10.77			
2LX7	CBCANH	600	N	128	104.87 131.37	C	128	16.30 81.30	HN	439	4.99 11.00			
2LX7	CBCAcoNH	600	N	128	104.87 131.37	C	128	9.30 74.30	HN	439	4.99 11.00			
2LX7	CCHTOCSY_@ALI	600	C1	256	0.08 70.09	C2	256	0.08 70.08	H1	398	-1.03 5.97			
2LX7	CCNOESY_@ALI	600	C1	64	13.29 33.99	C2	64	13.29 33.99	H1	1024	-3.70 9.64	H2	128	-0.53 6.47
2LX7	CcoNH	600	N	128	104.75 131.25	C	128	11.00 81.00	HN	370	5.48 11.99			
2LX7	HBHAcoNH	600	N	128	104.75 131.25	HN	371	5.48 11.99	H	128	0.51 6.51			
2LX7	HCCHCOSY_@ALI	600	C	256	7.36 77.35	HC	128	-0.53 6.47	H	542	-2.02 7.50			
2LX7	HCCHTOCSY_@ALI	600	C	256	7.11 77.11	HC	256	-0.53 6.47	H	541	-2.03 7.50			
2LX7	HCcoNH_@ALI	600	N	128	104.75 131.25	H	256	-0.72 6.28	HN	371	5.47 11.99			
2LX7	HNCA	600	N	128	104.75 131.25	C	128	41.00 71.00	HN	286	5.97 11.00			
2LX7	HNCO	600	N	128	104.75 131.25	C	128	167.01 180.93	HN	399	4.97 11.99			
2LX7	HNcoCA	600	N	128	104.75 131.25	C	128	41.00 71.00	HN	370	5.48 11.99			
2LX7	N15HSQC	600	N	1024	103.00 133.00	H	342	4.99 11.00						
2LX7	N15NOESY	850	N	256	104.85 131.35	HN	343	5.00 11.00	H	512	-1.22 10.78			
2MA6	C13HSQC_@ALI	850	C	1024	7.65 77.68	H	431	-0.50 5.81						
2MA6	C13HSQC_@ARO	850	C	256	109.91 139.92	H	206	5.99 9.00						
2MA6	C13NOESY_@ALI	850	C	128	8.60 70.56	HC	431	-0.51 5.81	H	256	-1.72 11.28			
2MA6	C13NOESY_@ARO	850	C	64	111.21 141.22	HC	206	5.99 9.00	H	256	-0.96 10.54			
2MA6	CBCANH	600	N	128	108.99 129.99	C	128	13.32 78.32	HN	302	5.68 10.99			
2MA6	CBCAcoNH	600	N	128	102.49 136.49	C	128	13.48 78.48	HN	302	5.68 10.99			
2MA6	CCHTOCSY_@ALI	600	C1	256	4.89 74.89	C2	256	4.89 74.89	H1	359	-0.53 5.79			
2MA6	CCNOESY_@ALI	600	C1	32	13.29 33.99	C2	32	13.29 33.99	H1	1024	-3.71 9.63	H2	128	-0.54 6.46
2MA6	CcoNH	850	N	128	109.07 130.08	C	128	6.65 76.68	HN	419	5.70 11.01			
2MA6	HBHAcoNH	600	N	128	108.99 129.99	HN	512	5.68 11.00	H	128	-0.24 5.76			
2MA6	HCCHCOSY_@ALI	850	C	512	9.13 79.16	HC	256	-0.47 6.53	H	553	-1.01 6.00			
2MA6	HCCHTOCSY_@ALI	600	C	128	10.58 74.58	HC	128	-0.45 6.55	H	512	-1.01 6.00			
2MA6	HCcoNH_@ALI	850	N	256	109.07 130.07	H	256	-0.48 6.53	HN	419	5.69 11.00			
2MA6	HNCO	600	N	256	102.49 136.49	C	128	167.01 180.93	HN	302	5.67 10.99			
2MA6	N15HSQC	850	N	2048	109.04 130.04	H	419	5.70 11.01						
2MA6	N15NOESY	850	N	128	109.07 130.07	HN	363	5.70 11.02	H	512	-1.71 11.29			
2JRM	C13HSQC_@ALI	800	C	512	5.30 80.26	H	2048	-2.14 11.80						
2JRM	C13NOESY_@ALI	800	C	128	33.71 57.71	HC	460	-1.08 5.19	H	256	-0.96 10.54			
2JRM	C13NOESY_@ARO	800	C	64	115.83 139.82	HC	1024	-2.18 11.77	H	256	-0.94 10.55			
2JRM	CBCANH	600	N	256	104.16 132.11	C	256	4.21 79.17	HN	1024	4.81 10.80			

PDB	Spectrum name	MHz	L1	Size	Range (ppm)		L2	Size	Range (ppm)		L3	Size	Range (ppm)		L4	Size	Range (ppm)	
2JRM	CBCAcoNH	600	N	256	104.16	132.11	C	256	4.29	79.25	HN	1024	4.82	10.80				
2JRM	CCHTOCSY_@ALI	600	C1	256	29.75	53.74	C2	256	4.29	79.25	H1	2048	-1.17	10.80				
2JRM	CcoNH	600	N	256	104.19	132.14	C	256	4.29	79.25	HN	1024	4.82	10.80				
2JRM	HBHAcoNH	600	N	256	104.16	132.11	HN	1024	4.81	10.80	H	256	0.31	6.31				
2JRM	HCCHTOCSY_@ALI	600	C	256	29.75	53.74	HC	256	-1.19	6.81	H	2048	-1.17	10.80				
2JRM	HNCO	600	N	256	104.19	132.14	C	256	168.84	188.71	HN	1024	4.81	10.80				
2JRM	N15HSQC	800	N	256	105.13	127.13	H	1024	4.85	11.82								
2JRM	N15NOESY	800	N	128	105.13	127.13	HN	956	4.95	11.46	H	256	-0.92	10.58				
1YEZ	C13HSQC_@ALI	600	C	1024	-3.09	76.48	H	1024	-1.42	11.09								
1YEZ	C13NOESY_@ALI	600	C	512	0.68	80.69	HC	533	-0.49	6.02	H	512	-1.42	11.10				
1YEZ	C13NOESY_@ARO	600	C	512	114.82	144.15	HC	130	6.13	7.72	H	412	-0.47	9.60				
1YEZ	CBCANH	500	N	512	100.68	136.20	C	512	4.46	79.47	HN	512	4.85	11.36				
1YEZ	CBCAcoNH	500	N	512	100.68	136.20	C	512	4.48	79.50	HN	512	4.87	11.38				
1YEZ	CcoNH	500	N	512	100.68	136.20	C	512	4.54	79.55	HN	512	4.88	11.39				
1YEZ	HBHAcoNH	500	N	512	100.64	136.16	HN	512	4.87	11.38	H	512	-1.85	7.14				
1YEZ	HCCHCOSY_@ALI	600	C	512	-3.30	76.70	HC	512	-1.44	11.07	H	533	-0.52	6.00				
1YEZ	HCCHTOCSY_@ARO	600	C	512	99.95	152.99	HC	512	-1.40	11.11	H	1024	-1.40	11.11				
1YEZ	HCcoNH_@ALI	500	N	512	100.69	136.21	H	512	-3.36	7.64	HN	512	4.87	11.38				
1YEZ	HNCA	500	N	512	100.63	136.15	C	512	41.00	73.06	HN	512	4.86	11.37				
1YEZ	HNCO	500	N	512	100.65	136.17	C	512	164.92	184.80	HN	512	4.86	11.37				
1YEZ	HNcaCO	500	N	512	100.64	136.15	C	512	166.93	182.83	HN	512	4.87	11.38				
1YEZ	HNcoCA	500	N	512	100.68	136.20	C	512	41.01	73.08	HN	512	4.87	11.38				
1YEZ	N15HSQC	600	N	512	100.17	136.37	H	512	4.84	11.09								
1YEZ	N15NOESY	600	N	512	101.00	135.55	HN	512	4.86	11.12	H	512	-1.39	11.12				
2L9R	C13NOESY_@ALI	800	C	128	19.77	89.77	HC	464	-1.36	4.96	H	412	-0.83	10.44				
2L9R	C13NOESY_@ARO	800	C	64	112.77	142.77	HC	264	4.63	8.23	H	256	-1.69	11.31				
2L9R	CBCANH	800	N	64	106.13	130.13	C	256	4.12	79.12	HN	512	4.80	11.77				
2L9R	CBCAcoNH	800	N	64	106.13	130.13	C	256	4.12	79.12	HN	512	4.80	11.77				
2L9R	CCHTOCSY_@ALI	800	C1	64	29.77	53.77	C2	128	4.27	79.27	H1	1024	-2.17	11.77				
2L9R	HBHAcoNH	800	N	64	106.13	130.13	HN	512	4.80	11.77	H	256	-0.44	6.56				
2L9R	HNCO	800	N	64	106.13	130.13	C	64	167.60	189.60	HN	512	4.80	11.77				
2L9R	N15HSQC	800	N	512	106.11	130.11	H	1024	4.80	11.77								
2L9R	N15NOESY	800	N	128	102.63	133.63	HN	324	6.00	10.41	H	452	-1.39	10.97				
2K52	C13HSQC	600	C	512	6.26	81.27	H	1024	-1.88	11.47								
2K52	C13NOESY_@ALI	800	C	128	19.83	89.83	HC	1027	-1.00	5.99	H	403	-1.01	10.01				
2K52	C13NOESY_@ARO	800	C	128	112.77	142.77	HC	221	6.00	9.02	H	256	-0.69	10.31				
2K52	CBCANH	600	N	128	102.12	132.12	C	128	4.31	79.33	HN	512	4.80	11.79				
2K52	CBCAcoNH	600	N	128	102.16	132.16	C	128	4.32	79.32	HN	512	4.80	11.79				
2K52	CCHTOCSY_@ALI	600	C1	128	31.91	55.91	C2	256	5.97	80.97	H1	514	-1.01	6.00				
2K52	HBHAcoNH	600	N	64	103.12	133.12	HN	512	4.80	11.79	H	256	0.29	6.29				
2K52	HCCHCOSY_@ALI	600	C	128	31.83	55.83	HC	128	-0.39	6.11	H	514	-1.02	6.00				
2K52	HCCHTOCSY_@ALI	600	C	128	31.80	55.80	HC	128	-0.39	6.11	H	514	-1.03	5.99				
2K52	HNCA	600	N	128	103.07	133.07	C	128	40.80	72.86	HN	512	4.79	11.78				
2K52	HNCO	600	N	128	104.23	134.23	C	128	167.72	189.80	HN	512	4.79	11.78				
2K52	HNcoCA	600	N	128	103.12	133.12	C	128	40.83	72.84	HN	512	4.79	11.78				
2K52	N15HSQC	800	N	256	100.63	135.63	H	512	4.81	11.78								
2K52	N15NOESY	800	N	128	104.22	135.05	HN	1024	4.81	11.78	H	403	-1.01	10.01				
2KRS	C13HSQC_@ALI	600	C	957	10.07	80.16	H	371	-0.33	6.18								
2KRS	C13HSQC_@ARO	600	C	256	110.00	140.00	H	192	5.98	8.79								
2KRS	C13NOESY_@ALI	850	C	128	10.80	78.80	HC	478	-0.51	6.49	H	256	-0.72	10.28				
2KRS	C13NOESY_@ARO	600	C	128	110.00	140.00	HC	192	5.98	8.79	H	512	-0.72	10.28				
2KRS	CBCANH	600	N	256	101.00	133.00	C	128	13.50	78.50	HN	257	5.98	10.50				
2KRS	CBCAcoNH	600	N	256	100.99	132.99	C	128	13.30	78.30	HN	285	6.21	11.23				
2KRS	CCHTOCSY_@ALI	600	C1	256	8.16	78.17	C2	512	8.12	78.12	H1	383	-0.53	6.19				
2KRS	CCNOESY_@ALI	600	C1	64	13.29	33.99	C2	32	13.29	33.99	H1	128	-0.53	6.47	H2	1024	-3.70	9.64
2KRS	CcoNH	600	N	256	101.01	132.99	C	128	8.50	83.50	HN	253	5.98	10.51				
2KRS	HBHAcoNH	600	N	128	101.01	132.99	HN	330	6.00	10.51	H	128	0.29	6.29				
2KRS	HCCHCOSY_@ALI	600	C	256	8.08	78.08	HC	256	-0.70	6.30	H	513	-0.49	6.52				
2KRS	HCCHTOCSY_@ALI	600	C	256	8.00	78.00	HC	241	-1.02	6.51	H	392	-0.51	6.51				
2KRS	HCcoNH_@ALI	600	N	256	101.01	132.99	H	256	-1.22	6.78	HN	253	5.98	10.51				
2KRS	HNCO	600	N	256	101.00	133.00	C	128	167.37	180.63	HN	257	5.98	10.50				
2KRS	HNcoCA	600	N	128	101.01	132.99	C	64	41.02	71.02	HN	258	5.99	10.51				
2KRS	N15NOESY	850	N	128	101.07	133.08	HN	258	5.99	10.51	H	256	-0.72	10.28				
2K53	C13HSQC_@ALI	800	C	256	4.86	78.86	H	478	-0.56	5.96								
2K53	C13NOESY_@ALI	800	C	512	19.81	89.81	HC	361	0.01	4.93	H	357	0.03	9.79				
2K53	C13NOESY_@ARO	800	C	256	112.89	142.88	HC	148	5.80	7.81	H	441	-0.23	10.10				
2K53	CBCANH	600	N	256	101.54	134.45	C	256	8.50	88.52	HN	512	4.78	11.45				
2K53	CBCAcoNH	600	N	256	101.58	134.49	C	203	14.26	77.70	HN	267	6.52	9.99				
2K53	CCHTOCSY_@ALI	600	C1	256	25.64	49.64	C2	256	-2.19	77.39	H1	462	-0.04	5.98				
2K53	HBHAcoNH	600	N	256	101.54	134.45	HN	288	6.70	10.45	H	110	0.94	5.24				
2K53	HCCHTOCSY_@ARO	600	C	156	115.48	135.68	HC	93	5.05	8.08	H	162	6.06	7.64				
2K53	HCcoNH_@ALI	600	N	240	103.07	133.92	H	146	0.08	5.79	HN	239	6.70	9.82				
2K53	N15HSQC	800	N	245	103.83	132.54	H	493	6.53	9.89								
2K53	N15NOESY_@ALI	800	N	493	103.56	133.01	HN	259	6.53	10.06	H	403	-0.57	10.45				
2JT1	C13HSQC_@ALI	800	C	1024	5.26	80.22	H	1024	-2.19	11.76								
2JT1	C13NOESY_@ALI	800	C	512	2.22	80.75	HC	495	-0.94	5.81	H	512	-0.23	9.77				
2JT1	C13NOESY_@ARO	800	C	256	112.86	142.87	HC	512	4.78	11.75	H	512	-0.97	10.53				
2JT1	CBCANH	800	N	256	104.64	131.64	C	512	4.18	79.26	HN	512	4.78	11.76				
2JT1	CBCAcoNH	800	N	256	104.64	131.64	C	512	4.18	79.25	HN	512	4.78	11.75				
2JT1	HBHAcoNH	800	N	256	104.61	131.60	HN	512	4.78	11.76	H	512	1.03	6.03				
2JT1	HNCO	800	N	256	104.62	131.62	C	512	168.74	188.74	HN	512	4.78	11.75				
2JT1	HNcaCO	800	N	256	104.61	131.60	C	512	168.76	188.75	HN	512	4.78	11.76				
2JT1	N15HSQC	800	N	1024	104.61	131.61	H	1024	4.79	11.76								
2JT1	N15NOESY	800	N	256	104.61	131.61	HN	512	4.79	11.77	H	512	-0.96	10.54				
2JVO	C13HSQC_@ALI	500	C	415	6.38	71.22	H	1020	-0.47	6.51								

PDB	Spectrum name	MHz	L1	Size	Range (ppm)		L2	Size	Range (ppm)		L3	Size	Range (ppm)		L4	Size	Range (ppm)	
2JVO	C13HSQC_@ARO	500	C	173	110.06	137.09	H	298	6.08	8.12								
2JVO	C13NOESY_@ALI	900	C	256	0.59	80.60	HC	993	-0.50	6.30	H	433	-0.36	11.48				
2JVO	CBCANH	500	N	256	94.17	130.17	C	256	10.95	72.95	HN	1024	4.70	11.70				
2JVO	CCHTOCSY_@ALI	600	C1	256	5.72	75.71	C2	256	5.74	75.74	H1	2048	-0.87	10.15				
2JVO	HCCHTOCSY_@ALI	500	C	256	5.47	71.51	HC	302	-0.54	6.54	H	585	-0.41	6.45				
2JVO	HNCA	500	N	256	94.11	130.11	C	256	40.28	72.28	HN	1024	4.71	11.71				
2JVO	HNcoCA	500	N	256	94.10	130.10	C	256	40.78	72.78	HN	1024	4.70	11.70				
2JVO	N15HSQC	600	N	512	94.83	130.83	H	1024	4.71	11.65								
2JVO	N15NOESY_@ALI	600	N	128	94.10	130.09	HN	1024	4.71	11.70	H	512	-2.29	11.71				
2ERR	C13HSQC_@ALI	600	C	460	5.43	73.70	H	1154	-0.41	6.34								
2ERR	C13NOESY	900	C	128	6.86	76.87	HC	609	-0.47	6.65	H	474	-0.94	10.15				
2ERR	CBCAcoNH	600	N	128	90.59	141.59	C	256	14.96	74.95	HN	1024	4.61	12.10				
2ERR	HCCHTOCSY_@ALI	600	C	128	6.61	72.23	HC	282	-0.81	6.62	H	521	-0.27	6.33				
2ERR	HNCA	600	N	120	101.57	133.44	C	128	40.49	74.49	HN	767	6.32	11.56				
2ERR	N15HSQC	600	N	512	98.70	138.70	H	1024	4.62	12.63								
2ERR	N15NOESY_@ALI	750	N	128	98.92	132.92	HN	1024	4.60	12.61	H	512	-3.40	12.59				
2L1P	C13HSQC	600	C	1024	-10.13	155.53	H	1024	-1.24	10.73								
2L1P	C13NOESY_@ALI	800	C	512	19.75	89.75	HC	537	-0.87	6.44	H	512	-2.25	11.75				
2L1P	C13NOESY_@ARO	800	C	512	113.57	141.57	HC	512	4.75	11.72	H	512	-2.25	11.75				
2L1P	CBCANH	600	N	256	101.20	135.20	C	256	4.21	79.22	HN	512	4.76	11.75				
2L1P	CCHTOCSY_@ALI	600	C1	256	29.73	53.73	C2	256	4.33	79.33	H1	477	-0.51	6.00				
2L1P	HBHAcoNH	600	N	256	100.13	136.13	HN	512	4.76	11.75	H	256	-1.24	6.76				
2L1P	HNCO	600	N	256	101.20	135.20	C	256	167.71	189.79	HN	512	4.76	11.75				
2L1P	N15HSQC	800	N	256	102.16	134.16	H	1024	4.75	11.73								
2L1P	N15NOESY	800	N	512	100.73	131.56	HN	512	4.75	11.72	H	512	-2.25	11.75				
2LN3	C13HSQC_@ALI	800	C	512	6.65	76.65	H	2048	-2.17	11.78								
2LN3	C13HSQC_@ARO	800	C	128	112.66	142.66	H	2048	-2.17	11.78								
2LN3	C13NOESY_@ALI	800	C	128	19.50	89.50	HC	504	-0.81	6.05	H	412	-0.83	10.44				
2LN3	C13NOESY_@ARO	800	C	64	112.77	142.77	HC	144	6.00	7.97	H	256	-1.69	11.31				
2LN3	CBCANH	800	N	64	104.13	132.13	C	256	4.12	79.12	HN	512	4.80	11.77				
2LN3	CBCAcoNH	800	N	64	104.13	132.13	C	256	4.12	79.12	HN	512	4.80	11.77				
2LN3	CCHTOCSY_@ALI	800	C1	64	29.58	53.58	C2	128	4.27	79.27	H1	1024	-2.17	11.77				
2LN3	HBHAcoNH	800	N	64	104.13	132.13	HN	512	4.80	11.77	H	256	-0.44	6.56				
2LN3	HNCO	800	N	64	104.13	132.13	C	64	167.60	189.60	HN	512	4.80	11.77				
2LN3	N15HSQC	800	N	512	104.10	132.10	H	1024	4.80	11.77								
2LN3	N15NOESY	800	N	128	102.63	133.63	HN	324	6.00	10.41	H	512	-2.19	11.80				
2HEQ	C13HSQC_@ALI	750	C	854	4.96	80.02	H	512	-2.02	7.00								
2HEQ	C13NOESY_@ALI	750	C	128	30.95	54.95	HC	512	-2.02	7.00	H	256	-1.69	11.31				
2HEQ	C13NOESY_@ALI_2	600	C	256	31.08	55.08	HC	481	-2.05	6.96	H	256	-0.23	9.77				
2HEQ	CBCANH	600	N	128	106.01	130.01	C	128	10.97	80.97	HN	378	4.99	12.01				
2HEQ	CBCAcoNH	600	N	128	106.01	130.01	C	128	10.95	80.94	HN	378	4.99	12.01				
2HEQ	CCNOESY_@ALI	600	C1	64	13.29	33.99	C2	32	13.26	33.95	H1	128	-0.52	6.48	H2	1024	-3.69	9.66
2HEQ	HCCHCOSY_@ALI	600	C	256	30.96	54.96	HC	241	-1.05	6.48	H	406	-1.04	6.49				
2HEQ	HCCHTOCSY_@ALI	600	C	256	30.96	54.96	HC	241	-1.05	6.48	H	406	-1.04	6.49				
2HEQ	HNCA	600	N	128	106.03	130.02	C	64	40.91	70.91	HN	378	4.99	12.01				
2HEQ	HNCO	600	N	128	106.02	130.03	C	64	166.04	181.96	HN	378	4.99	12.01				
2HEQ	HNcoCA	600	N	128	106.01	130.01	C	64	40.97	70.97	HN	378	4.99	12.01				
2HEQ	N15HSQC	750	N	512	105.96	129.96	H	444	4.50	12.02								
2HEQ	N15NOESY	600	N	256	105.99	129.99	HN	400	4.98	12.01	H	512	-1.69	11.31				
2KK8	C13HSQC_@ALI	600	C	512	-2.17	77.41	H	998	-0.50	6.00								
2KK8	C13NOESY_@ALI	800	C	256	19.82	89.82	HC	478	-0.52	5.99	H	512	-2.15	11.85				
2KK8	C13NOESY_@ARO	800	C	256	114.07	142.07	HC	512	4.85	11.82	H	256	-2.10	11.90				
2KK8	CBCANH	600	N	256	101.45	134.36	C	256	8.51	88.52	HN	512	4.81	11.48				
2KK8	CBCAcoNH	600	N	256	99.91	136.11	C	256	12.13	85.08	HN	256	4.82	11.49				
2KK8	CCHTOCSY_@ALI	600	C1	256	25.66	49.66	C2	256	1.13	74.08	H1	270	-0.53	6.50				
2KK8	CcoNH	600	N	256	99.91	136.11	C	256	12.13	85.08	HN	512	4.82	11.49				
2KK8	HCcoNH_@ALI	600	N	512	98.98	135.19	H	512	-0.18	9.82	HN	512	4.82	11.49				
2KK8	HNCA	600	N	256	101.55	134.46	C	256	34.68	74.47	HN	256	4.82	11.82				
2KK8	HNCO	600	N	256	99.91	136.11	C	256	167.97	185.21	HN	256	4.82	11.49				
2KK8	N15HSQC	800	N	256	101.14	135.14	H	512	4.84	11.81								
2KK8	N15NOESY	800	N	256	102.73	133.56	HN	512	4.84	11.81	H	256	-2.17	11.83				
2KD0	C13HSQC_@ALI	800	C	512	4.95	78.95	H	515	-0.52	6.50								
2KD0	C13NOESY_@ALI	800	C	256	19.98	89.98	HC	478	-0.52	5.99	H	512	-2.15	11.85				
2KD0	C13NOESY_@ARO	800	C	256	113.75	141.75	HC	512	4.85	11.82	H	256	-2.15	11.85				
2KD0	CBCANH	800	N	256	99.26	133.26	C	256	4.40	79.41	HN	512	4.85	11.83				
2KD0	CBCAcoNH	800	N	256	99.22	133.22	C	256	4.31	79.32	HN	512	4.85	11.83				
2KD0	CCHTOCSY_@ALI	800	C1	256	29.96	53.96	C2	256	6.90	76.90	H1	508	-0.52	6.40				
2KD0	HBHAcoNH	800	N	256	99.18	133.18	HN	512	4.85	12.86	H	256	0.35	6.35				
2KD0	HCCHTOCSY_@ALI	800	C	256	30.12	54.12	HC	256	-0.40	6.60	H	515	-0.52	6.50				
2KD0	HCcoNH_@ALI	600	N	256	99.19	133.19	H	256	-1.18	6.83	HN	512	4.84	11.83				
2KD0	HNCO	800	N	256	99.14	133.14	C	256	167.77	189.86	HN	512	4.85	11.82				
2KD0	HNcaCO	800	N	256	99.18	133.18	C	256	167.77	189.86	HN	512	4.85	11.82				
2KD0	N15HSQC	800	N	512	99.20	133.20	H	512	4.85	11.83								
2KD0	N15NOESY	800	N	256	100.76	131.59	HN	512	4.85	11.82	H	256	-2.15	11.85				
2LML	C13HSQC_@ALI	600	C	512	3.09	83.09	H	359	-1.02	5.99								
2LML	C13NOESY_@ALI	850	C	256	6.80	76.80	HC	401	-1.01	6.01	H	256	-1.05	10.65				
2LML	C13NOESY_@ARO	850	C	64	112.30	140.31	HC	172	5.99	9.00	H	256	-1.08	10.68				
2LML	CBCANH	600	N	128	103.00	133.00	C	128	13.65	78.65	HN	258	5.96	11.00				
2LML	CBCAcoNH	600	N	256	102.97	132.97	C	128	13.50	78.50	HN	386	5.96	13.50				
2LML	CCNOESY_@ALI	600	C1	64	13.29	33.99	C2	64	13.29	33.99	H1	1024	-3.68	9.65	H2	128	-0.51	6.48
2LML	CcoNH	600	N	256	102.97	132.97	C	128	13.50	78.50	HN	258	5.97	11.01				
2LML	HBHAcoNH	850	N	256	102.79	133.34	HN	315	5.49	11.00	H	256	0.39	6.27				
2LML	HCCHCOSY_@ALI	850	C	128	6.80	76.80	HC	256	-1.41	5.59	H	401	-1.03	5.99				
2LML	HCCHTOCSY_@ALI	850	C	256	6.80	76.80	HC	256	-1.47	5.53	H	401	-1.01	6.01				
2LML	HCcoNH_@ALI	600	N	256	102.97	132.97	H	128	-0.72	6.28	HN	258	5.97	11.01				

PDB	Spectrum name	MHz	L1	Size	Range (ppm)		L2	Size	Range (ppm)		L3	Size	Range (ppm)		L4	Size	Range (ppm)	
2LML	HNCO	600	N	256	102.97	132.97	C	128	165.50	182.50	HN	258	5.97	11.01				
2LML	HNcoCA	600	N	256	102.97	132.97	C	128	41.07	71.07	HN	386	5.97	13.51				
2LML	N15HSQC	850	N	256	103.08	133.08	H	308	5.99	10.50								
2LML	N15NOESY	850	N	128	103.00	133.08	HN	315	4.98	10.50	H	256	-1.08	10.68				
2K3D	C13HSQC_@ARO	850	C	512	112.78	142.78	H	240	5.49	9.01								
2K3D	C13NOESY_@ALI	850	C	256	6.48	81.32	HC	478	-1.00	6.00	H	256	-0.72	10.28				
2K3D	C13NOESY_@ARO	850	C	256	114.77	140.77	HC	240	5.49	9.01	H	256	-0.70	10.30				
2K3D	CBCANH	850	N	128	103.62	132.64	C	128	9.90	79.72	HN	308	5.49	10.00				
2K3D	CBCAcoNH	850	N	128	103.62	132.64	C	256	10.39	80.21	HN	308	5.48	10.00				
2K3D	CCNOESY_@ALI	600	C1	64	13.29	33.99	C2	64	13.29	33.99	H1	1024	-3.67	9.66	H2	128	-0.50	6.49
2K3D	HBHAcoNH	850	N	128	103.59	132.61	HN	308	5.48	10.00	H	256	0.39	6.27				
2K3D	HCCHTOCSY_@ALI	850	C	128	6.70	76.70	HC	256	-0.41	6.59	H	479	-0.98	6.04				
2K3D	HNCA	850	N	64	103.55	132.57	C	128	41.20	71.19	HN	308	5.48	10.00				
2K3D	HNCO	850	N	128	103.59	132.61	C	128	166.69	183.70	HN	274	5.99	10.00				
2K3D	N15HSQC	600	N	256	103.47	132.47	H	343	4.97	11.00								
2K3D	N15NOESY	850	N	256	103.59	132.61	HN	410	5.49	11.50	H	256	-0.70	10.30				
2LK2	C13HSQC_@ALI	850	C	1024	3.15	88.15	H	429	-1.01	6.50								
2LK2	C13HSQC_@ARO	850	C	256	108.00	142.00	H	274	5.48	9.50								
2LK2	C13NOESY_@ALI	850	C	512	12.85	78.84	HC	458	-1.52	6.50	H	256	-1.22	10.78				
2LK2	C13NOESY_@ARO	850	C	256	111.30	141.30	HC	274	5.48	9.50	H	256	-1.22	10.78				
2LK2	CBCANH	600	N	256	103.00	132.00	C	256	12.92	78.92	HN	343	5.48	11.51				
2LK2	CBCAcoNH	600	N	256	103.00	132.00	C	128	14.00	78.00	HN	371	5.48	12.00				
2LK2	CCNOESY_@ALI	600	C1	32	13.29	33.99	C2	32	13.29	33.99	H1	1024	-3.70	9.64	H2	128	-0.53	6.47
2LK2	CcoNH	600	N	128	103.00	132.00	C	128	8.50	83.50	HN	401	5.48	12.00				
2LK2	HBHAcoNH	600	N	128	103.00	132.00	HN	371	5.49	12.00	H	128	0.04	7.04				
2LK2	HCCHCOSY_@ALI	600	C	256	3.00	83.00	HC	110	-1.06	6.50	H	462	-1.01	6.51				
2LK2	HCCHTOCSY_@ALI	600	C	256	7.92	77.92	HC	256	-1.21	6.79	H	462	-1.00	6.51				
2LK2	HCcoNH_@ALI	600	N	256	103.00	132.00	H	256	-0.70	6.30	HN	371	5.49	12.00				
2LK2	HNCA	600	N	256	103.00	132.00	C	128	40.93	70.93	HN	371	5.49	12.00				
2LK2	HNCO	600	N	128	103.00	132.00	C	64	167.97	183.97	HN	371	5.48	11.99				
2LK2	HNcoCA	600	N	256	103.00	132.00	C	128	40.86	70.86	HN	371	5.49	12.00				
2LK2	N15HSQC	600	N	1024	103.01	133.01	H	445	5.48	12.00								
2LK2	N15NOESY	850	N	128	103.07	132.07	HN	287	5.48	10.51	H	256	-0.72	10.28				
MH04	C13HSQC_@ALI	600	C	512	6.15	84.41	H	1024	-1.92	11.42								
MH04	C13NOESY	800	C	128	5.64	80.21	HC	2048	-2.14	11.61	H	512	-2.15	11.61				
MH04	CBCANH	600	N	256	101.31	135.31	C	256	7.38	87.38	HN	1024	4.77	11.44				
MH04	CBCAcoNH	600	N	256	101.32	135.32	C	256	9.91	84.91	HN	1024	4.77	11.44				
MH04	CcoNH	600	N	128	101.32	135.32	C	256	6.48	88.48	HN	1024	4.77	11.44				
MH04	HBHAcoNH	600	N	128	101.29	135.29	HN	1024	4.78	11.43	H	256	-1.75	11.25				
MH04	HCCHTOCSY_@ALI	600	C	512	-7.18	72.86	HC	512	-1.50	11.00	H	639	-1.51	11.00				
MH04	HNCA	600	N	256	101.25	135.25	C	128	42.38	72.38	HN	512	4.76	11.43				
MH04	HNcaCO	600	N	256	101.25	135.25	C	128	162.87	187.87	HN	1024	4.76	11.43				
MH04	HNcoCA	600	N	256	101.28	135.28	C	128	42.38	72.38	HN	1024	4.76	11.43				
MH04	N15HSQC	600	N	1024	101.30	135.30	H	1024	4.77	11.44								
MH04	N15NOESY	800	N	128	101.39	135.39	HN	1024	4.76	11.41	H	256	-1.91	11.39				
1PQX	C13HSQC_@ALI	600	C	488	-3.42	76.14	H	2048	-1.88	11.46								
1PQX	C13HSQC_@ARO	600	C	512	106.46	146.24	H	2048	-1.89	11.45								
1PQX	C13NOESY_@ALI	600	C	512	-3.63	76.38	HC	654	-1.50	7.01	H	512	-1.89	11.45				
1PQX	C13NOESY_@ARO	600	C	256	2.17	48.58	HC	512	4.77	11.43	H	512	-1.90	11.44				
1PQX	CcoNH	600	N	256	103.08	135.99	C	512	5.60	89.14	HN	512	4.77	11.43				
1PQX	HCCHCOSY_@ALI	600	C	256	-3.72	76.28	HC	512	-0.65	10.18	H	1024	-1.90	11.42				
1PQX	HCcoNH	600	N	256	103.12	136.03	H	512	-0.65	10.19	HN	512	4.77	11.43				
1PQX	HNCA	600	N	256	103.12	136.03	C	512	39.16	75.62	HN	1024	-1.89	11.43				
1PQX	HNCO	600	N	256	103.12	136.03	C	512	167.11	183.69	HN	2048	-1.89	11.43				
1PQX	HNcoCA	600	N	256	103.12	136.03	C	512	39.14	75.60	HN	512	4.77	11.43				
1PQX	N15HSQC	600	N	2048	99.93	136.12	H	1024	-2.27	11.90								
1PQX	N15NOESY	600	N	256	103.08	135.99	HN	512	4.77	11.43	H	512	-1.90	11.44				
2L33	C13HSQC_@ALI	800	C	512	6.46	76.45	H	2048	-2.22	11.73								
2L33	C13HSQC_@ARO	800	C	1024	112.62	142.62	H	1024	-2.21	11.74								
2L33	C13NOESY_@ALI	800	C	128	19.45	89.45	HC	524	-0.84	6.29	H	512	-2.23	11.77				
2L33	C13NOESY_@ARO	800	C	64	112.60	142.60	HC	124	5.97	7.66	H	512	-1.73	11.27				
2L33	CBCANH	600	N	64	104.20	130.53	C	128	13.19	83.20	HN	512	4.75	10.76				
2L33	CBCAcoNH	600	N	64	101.78	133.04	C	128	13.25	83.26	HN	512	4.75	10.76				
2L33	CCHTOCSY_@ALI	800	C1	64	29.41	53.41	C2	128	4.00	79.00	H1	1024	-2.21	11.74				
2L33	HBHAcoNH	800	N	64	102.00	133.00	HN	512	4.77	11.74	H	256	0.27	6.27				
2L33	N15HSQC	800	N	512	102.01	133.01	H	1024	4.77	11.74								
2L33	N15NOESY	800	N	128	101.76	132.94	HN	253	6.28	9.72	H	376	-0.29	9.99				
2KZV	C13HSQC_@ALI	850	C	449	9.85	80.00	H	449	-0.51	6.50								
2KZV	C13HSQC_@ARO	850	C	256	112.00	142.00	H	274	5.48	9.50								
2KZV	C13NOESY_@ALI	850	C	256	12.92	78.92	HC	429	-1.02	6.50	H	256	-1.22	10.78				
2KZV	C13NOESY_@ARO	850	C	256	111.30	141.30	HC	229	5.99	10.00	H	256	-1.22	10.78				
2KZV	CBCANH	600	N	128	105.14	128.84	C	128	16.02	76.02	HN	286	5.48	10.51				
2KZV	CBCAcoNH	600	N	128	105.14	128.84	C	128	16.02	76.02	HN	286	5.48	10.50				
2KZV	CCHTOCSY_@ALI	600	C1	256	8.18	78.19	C2	256	8.18	78.18	H1	428	-1.02	6.50				
2KZV	CCNOESY_@ALI	600	C1	64	13.29	33.99	C2	64	13.29	33.99	H1	1024	-3.70	9.64	H2	128	-0.53	6.47
2KZV	CcoNH	600	N	128	105.14	128.84	C	256	11.18	77.18	HN	286	5.97	11.00				
2KZV	HBHAcoNH	600	N	128	105.14	128.84	HN	286	5.48	10.50	H	128	0.51	6.51				
2KZV	HCCHCOSY_@ALI	600	C	256	13.03	83.02	HC	256	-0.21	5.79	H	400	-0.52	6.50				
2KZV	HCCHTOCSY_@ALI	600	C	256	13.02	83.03	HC	256	-0.21	5.79	H	400	-0.52	6.51				
2KZV	HNCA	600	N	128	105.14	128.84	C	128	41.01	71.01	HN	286	5.49	10.51				
2KZV	HNCO	600	N	128	105.14	128.84	C	64	168.00	183.91	HN	286	5.48	10.51				
2KZV	HNcoCA	600	N	128	105.14	128.84	C	128	41.01	71.01	HN	286	5.48	10.51				
2KZV	N15HSQC	850	N	1024	105.25	128.95	H	287	5.48	10.51								
2KZV	N15NOESY	850	N	256	105.26	128.96	HN	287	5.48	10.51	H	512	-1.22	10.78				
2KCT	C13HSQC_@ALI	800	C	1024	6.77	76.77	H	2048	-2.17	11.78								

PDB	Spectrum name	MHz	L1	Size	Range (ppm)	L2	Size	Range (ppm)	L3	Size	Range (ppm)	L4	Size	Range (ppm)
2KCT	C13HSQC_@ARO	600	C	1024	112.75 142.75	H	2048	-1.86 11.49						
2KCT	C13NOESY_@ALI	800	C	512	19.77 89.77	HC	552	-1.01 6.50	H	440	-1.04 10.99			
2KCT	C13NOESY_@ARO	800	C	256	112.70 142.70	HC	512	4.80 11.77	H	512	-1.20 10.80			
2KCT	CBCANH	600	N	256	103.08 132.70	C	512	8.53 88.54	HN	512	4.79 11.46			
2KCT	CBCAcoNH	600	N	256	103.10 132.72	C	512	8.58 88.59	HN	512	4.79 11.46			
2KCT	CCHTOCSY_@ALI	600	C1	256	31.75 55.75	C2	256	8.83 78.83	H1	551	-1.03 6.50			
2KCT	CcoNH	600	N	256	103.08 132.70	C	512	12.10 85.05	HN	512	4.79 11.46			
2KCT	HBHAcoNH	600	N	256	103.08 132.70	HN	512	4.79 11.46	H	512	-0.23 9.78			
2KCT	HCCHCOSY_@ALI	600	C	256	31.75 55.75	HC	512	-0.08 6.42	H	550	-1.02 6.49			
2KCT	HCCHTOCSY_@ALI	600	C	256	31.75 55.75	HC	512	-0.08 6.42	H	1024	-2.19 11.78			
2KCT	HNcaCO	600	N	256	101.48 134.39	C	256	164.28 188.81	HN	512	4.79 11.46			
2KCT	HNcoCA	600	N	256	103.12 132.74	C	512	42.64 74.47	HN	512	4.79 11.46			
2KCT	N15HSQC	800	N	1024	103.13 133.13	H	1024	4.81 11.78						
2KCT	N15NOESY	800	N	256	104.22 135.04	HN	512	4.81 11.78	H	440	-1.04 10.99			
2MDR	C13HSQC_@ALI	900	C	512	11.03 44.36	H	960	-1.21 6.28						
2MDR	C13NOESY_@ALI	900	C	256	11.03 44.33	HC	832	-0.90 6.41	H	512	-0.59 10.01			
2MDR	C13NOESY_@ARO	600	C	256	112.99 134.99	HC	320	6.20 8.39	H	512	-0.60 10.00			
2MDR	CBCANH	500	N	128	104.15 133.15	C	256	12.46 74.46	HN	1024	4.73 11.73			
2MDR	CBCAcoNH	600	N	256	104.38 127.38	C	256	13.03 73.03	HN	640	5.99 10.99			
2MDR	CCHTOCSY_@ALI	600	C1	256	10.04 72.05	C2	256	10.07 72.07	H1	1216	-0.94 6.48			
2MDR	HCCHTOCSY_@ALI	600	C	256	10.01 72.01	HC	512	-0.61 9.99	H	1216	-1.24 6.49			
2MDR	HNCA	600	N	256	104.37 127.37	C	256	39.49 68.49	HN	608	5.97 10.73			
2MDR	N15HSQC	600	N	1024	104.07 133.07	H	928	4.74 11.99						
2MDR	N15NOESY	900	N	256	104.30 127.30	HN	672	5.42 10.99	H	512	-0.60 10.00			
2FB7	C13NOESY_@ALI	600	C	215	9.72 76.92	HC	860	-0.50 6.50	H	338	-1.03 9.99			
2FB7	CBCANH	600	N	128	103.24 135.50	C	256	8.65 88.66	HN	1024	4.77 13.12			
2FB7	CBCAcoNH	600	N	128	103.25 135.51	C	256	8.96 88.97	HN	1024	4.77 13.12			
2FB7	CCHTOCSY_@ALI	600	C1	256	-2.04 77.97	C2	256	-2.08 77.92	H1	2048	-3.56 13.12			
2FB7	CcoNH	600	N	128	103.25 135.52	C	256	8.98 88.98	HN	1024	4.78 13.12			
2FB7	HBHAcoNH	600	N	128	103.25 135.52	HN	1024	4.77 13.11	H	256	-1.90 11.44			
2FB7	HCCHTOCSY_@ALI	600	C	216	9.43 76.93	HC	256	-1.08 10.60	H	860	-0.52 6.49			
2FB7	HCcoNH_@ALI	600	N	128	103.25 135.52	H	256	-3.58 13.11	HN	1024	4.77 13.11			
2FB7	HNCO	600	N	128	103.23 135.50	C	128	167.88 182.11	HN	1024	4.77 13.11			
2FB7	N15HSQC	600	N	256	103.25 135.52	H	1024	4.77 13.12						
2FB7	N15NOESY	600	N	116	104.75 133.99	HN	431	6.49 10.01	H	423	-0.52 10.50			
2MB0	C13HSQC_@ALI	900	C	932	10.34 74.05	H	877	-0.63 6.20						
2MB0	C13NOESY_@ALI	900	C	256	8.50 73.50	HC	962	-0.99 6.51	H	436	-0.39 10.66			
2MB0	C13NOESY_@ARO	700	C	64	117.00 139.00	HC	290	5.84 8.10	H	325	-0.36 9.80			
2MB0	CcoNH	500	N	256	100.79 130.79	C	511	5.16 70.97	HN	259	6.56 10.62			
2MB0	HCcoNH_@ALI	500	N	256	100.79 130.79	H	295	-0.54 6.38	HN	297	6.27 10.91			
2MB0	HNCA	500	N	256	100.80 130.80	C	106	41.00 67.50	HN	509	6.66 10.64			
2MB0	N15HSQC	900	N	1001	101.10 130.42	H	591	6.16 10.77						
2MB0	N15NOESY_@ALI	900	N	128	100.80 130.80	HN	618	6.22 11.04	H	358	-0.41 10.80			
2L05	C13HSQC	800	C	1024	6.78 76.78	H	2048	-2.16 11.79						
2L05	C13NOESY_@ALI	800	C	512	29.76 53.76	HC	588	-1.51 6.50	H	512	-1.19 10.81			
2L05	C13NOESY_@ARO	800	C	512	112.76 142.76	HC	512	4.81 11.79	H	512	-1.19 10.81			
2L05	CBCANH	600	N	256	100.67 135.67	C	512	4.18 79.19	HN	512	4.81 11.80			
2L05	CBCAcoNH	600	N	256	100.63 135.63	C	512	4.27 79.27	HN	512	4.81 11.80			
2L05	CCHTOCSY_@ALI	600	C1	512	31.78 55.78	C2	512	6.32 81.32	H1	587	-1.52 6.50			
2L05	HCCHCOSY_@ALI	600	C	512	31.82 55.82	HC	512	-0.44 6.56	H	587	-1.52 6.49			
2L05	HCCHTOCSY_@ALI	600	C	512	31.78 55.78	HC	512	-0.44 6.56	H	587	-1.51 6.50			
2L05	HNCA	600	N	256	100.67 135.67	C	512	40.65 72.71	HN	512	4.81 11.80			
2L05	HNCO	600	N	256	100.71 135.71	C	256	167.71 189.79	HN	512	4.81 11.80			
2L05	HNcaCO	600	N	256	100.67 135.67	C	256	167.76 189.84	HN	512	4.81 11.80			
2L05	HNcoCA	600	N	256	100.67 135.67	C	512	40.71 72.72	HN	512	4.81 11.80			
2L05	N15NOESY	800	N	256	105.11 132.11	HN	512	4.81 11.78	H	512	-1.19 10.81			
2KJR	C13HSQC_@ALI	850	C	449	9.86 80.02	H	411	-0.02 6.00						
2KJR	C13NOESY_@ALI	850	C	256	7.80 77.80	HC	390	-0.52 6.31	H	256	-1.00 10.60			
2KJR	C13NOESY_@ARO	850	C	64	115.80 140.80	HC	201	5.48 9.00	H	256	-1.00 10.60			
2KJR	CBCANH	500	N	64	103.00 135.00	C	128	11.00 81.00	HN	462	5.98 10.49			
2KJR	CBCAcoNH	500	N	128	103.00 135.00	C	128	11.00 81.00	HN	462	5.98 10.49			
2KJR	CCHTOCSY_@ALI	500	C1	512	8.04 78.04	C2	256	8.00 78.00	H1	385	-1.03 6.49			
2KJR	CcoNH	500	N	256	102.96 134.96	C	128	8.50 83.50	HN	330	5.97 10.49			
2KJR	HCCHTOCSY_@ALI	500	C	512	8.00 78.00	HC	128	-0.76 6.24	H	487	-2.04 7.47			
2KJR	HNCA	500	N	128	103.00 135.00	C	64	40.86 70.86	HN	462	6.00 10.51			
2KJR	HNCO	500	N	128	103.00 135.00	C	64	166.05 181.96	HN	232	5.97 10.51			
2KJR	N15HSQC	850	N	256	103.11 135.11	H	257	5.99 10.49						
2KJR	N15NOESY	850	N	128	103.08 135.07	HN	257	5.99 10.49	H	256	-1.00 10.60			
2M5O	C13NOESY_@ALI	800	C	128	19.50 89.50	HC	464	-0.54 5.78	H	412	-0.82 10.44			
2M5O	C13NOESY_@ARO	800	C	64	112.78 142.78	HC	144	6.01 7.97	H	256	-1.69 11.31			
2M5O	CBCANH	800	N	128	102.51 133.51	C	256	3.98 78.98	HN	512	4.80 11.78			
2M5O	CBCAcoNH	800	N	64	102.63 133.63	C	256	4.13 79.13	HN	512	4.80 11.78			
2M5O	CCHTOCSY_@ALI	800	C1	128	29.49 53.49	C2	128	3.98 78.98	H1	1024	-2.17 11.78			
2M5O	HBHAcoNH	800	N	128	103.13 133.13	HN	512	4.81 11.78	H	256	-0.44 6.56			
2M5O	HNCO	800	N	64	102.63 133.63	C	128	167.69 189.69	HN	512	4.80 11.78			
2M5O	N15HSQC	800	N	256	105.99 129.99	H	1024	4.79 11.77						
2M5O	N15NOESY	800	N	128	102.51 133.51	HN	304	6.27 10.41	H	422	-0.55 10.98			
MDM2	C13NOESY	700	C	64	-1.66 28.34	HC	880	-0.82 5.32	H	405	-0.55 9.72			
MDM2	CBCANH	700	N	64	99.89 131.21	C	256	10.10 74.64	HN	647	5.97 10.48			
MDM2	HCCHTOCSY_@ALI	700	C	128	3.79 78.82	HC	203	-0.67 5.68	H	741	-0.36 5.40			
MDM2	HNCA	700	N	128	100.11 131.43	C	256	46.09 74.49	HN	646	5.98 10.48			
MDM2	HNcoCA	700	N	128	100.10 131.42	C	97	42.73 69.63	HN	534	6.28 10.00			
MDM2	N15NOESY	700	N	64	97.13 136.28	HN	651	5.68 10.22	H	424	-0.83 9.92			
MDM2	N15TOCSY	700	N	64	101.02 132.34	HN	575	5.73 9.74	H	512	-2.53 11.75			

PDB	Spectrum name	MHz	L1	Size	Range (ppm)		L2	Size	Range (ppm)		L3	Size	Range (ppm)		L4	Size	Range (ppm)	
2LNA	C13HSQC_@ALI	850	C	1024	3.20	88.26	H	417	-0.50	6.01								
2LNA	C13HSQC_@ARO	850	C	256	109.91	139.92	H	342	4.99	10.00								
2LNA	C13NOESY_@ALI	850	C	256	11.74	77.72	HC	476	-0.50	6.01	H	256	-2.22	11.78				
2LNA	C13NOESY_@ARO	600	C	64	110.72	140.72	HC	293	4.99	9.01	H	512	-1.43	10.97				
2LNA	CBCANH	600	N	256	103.98	133.98	C	256	13.40	78.40	HN	285	5.97	10.99				
2LNA	CBCAcoNH	600	N	256	103.98	133.98	C	128	13.48	78.48	HN	285	5.97	10.99				
2LNA	CCHTOCSY_@ALI	600	C1	256	-5.02	74.97	C2	256	-4.93	75.07	H1	427	-1.03	6.48				
2LNA	CCNOESY_@ALI	600	C1	32	13.29	33.99	C2	32	13.29	33.99	H1	1024	-3.70	9.64	H2	128	-0.53	6.47
2LNA	CcoNH	600	N	128	103.98	133.98	C	256	10.97	80.97	HN	286	5.97	11.00				
2LNA	HBHAcoNH	600	N	128	103.99	133.99	HN	286	5.97	11.00	H	256	0.37	6.37				
2LNA	HCCHCOSY_@ALI	600	C	256	2.73	72.70	HC	256	-0.44	6.56	H	512	-0.98	6.03				
2LNA	HCCHTOCSY_@ALI	600	C	512	-0.07	69.94	HC	256	-0.72	6.28	H	383	-0.52	6.20				
2LNA	HCcoNH_@ALI	600	N	128	103.98	133.98	H	256	-0.53	6.47	HN	286	5.97	11.00				
2LNA	HNCO	600	N	128	103.98	133.98	C	64	167.01	180.93	HN	286	5.99	11.01				
2LNA	HNcoCA	600	N	128	103.98	133.98	C	64	40.98	70.98	HN	285	5.99	11.00				
2LNA	N15HSQC	600	N	256	104.04	134.04	H	285	5.99	11.01								
2LNA	N15NOESY	850	N	128	104.07	134.07	HN	440	4.99	11.01	H	512	-0.81	10.39				
2LA6	C13NOESY_@ALI	800	C	128	19.50	89.50	HC	524	-0.81	6.33	H	512	-2.21	11.79				
2LA6	C13NOESY_@ARO	800	C	64	112.77	142.77	HC	264	4.64	8.24	H	256	-1.69	11.31				
2LA6	CBCANH	600	N	64	103.04	132.66	C	128	13.24	83.25	HN	512	4.79	11.46				
2LA6	CBCAcoNH	600	N	64	103.04	132.66	C	128	13.24	83.25	HN	512	4.79	11.46				
2LA6	CCHTOCSY_@ALI	600	C1	128	25.23	49.24	C2	256	0.90	73.85	H1	1024	-1.89	11.45				
2LA6	CcoNH	600	N	64	103.04	132.66	C	128	8.73	88.31	HN	512	4.79	11.46				
2LA6	HBHAcoNH	600	N	64	103.04	132.66	HN	512	4.79	11.46	H	128	-0.21	9.79				
2LA6	HNCO	600	N	64	103.04	132.66	C	64	167.80	185.04	HN	512	4.79	11.46				
2LA6	N15HSQC	600	N	512	102.98	132.60	H	1024	4.79	11.46								
2LA6	N15NOESY	800	N	128	102.51	133.51	HN	324	6.00	10.41	H	512	-2.21	11.79				
6FIP	C13HSQC_@ALI	600	C	512	3.16	83.22	H	1075	-1.01	6.00								
6FIP	C13NOESY_@ALI	600	C	128	3.19	83.19	HC	1323	-0.51	6.50	H	1024	-2.33	11.86				
6FIP	CBCANH	800	N	128	102.47	137.01	C	256	9.11	83.11	HN	746	5.99	11.00				
6FIP	CBCAcoNH	800	N	128	102.47	137.01	C	256	9.11	83.10	HN	746	5.99	11.00				
6FIP	CcoNH	800	N	128	102.50	137.04	C	256	6.07	86.07	HN	746	6.00	11.01				
6FIP	HBHAcoNH	800	N	128	102.47	137.01	HN	746	5.99	11.00	H	256	-2.43	11.95				
6FIP	HCCHCOSY_@ALI	600	C	128	-4.81	75.19	HC	172	-0.24	6.48	H	1323	-0.51	6.50				
6FIP	HCCHTOCSY_@ALI	600	C	128	-5.21	74.79	HC	166	-0.53	6.50	H	1323	-0.51	6.50				
6FIP	HNCO	800	N	128	102.47	137.01	C	128	167.05	183.74	HN	746	5.99	11.00				
6FIP	HNcaCO	800	N	128	102.47	137.01	C	128	165.02	183.77	HN	746	5.99	11.00				
6FIP	N15HSQC	800	N	1024	102.45	137.00	H	1024	4.76	11.63								
6FIP	N15NOESY	800	N	256	102.47	137.01	HN	1024	4.76	11.64	H	856	-0.52	11.50				
6FIP	N15TOCSY_@ALI	800	N	128	102.44	136.98	HN	746	5.99	11.00	H	256	-2.43	11.95				
2LEA	C13HSQC_@ALI	900	C	1024	8.85	69.85	H	2048	-3.37	12.59								
2LEA	C13HSQC_@ARO	600	C	404	115.94	139.61	H	369	6.08	8.96								
2LEA	C13NOESY_@ALI	900	C	256	13.66	45.66	HC	895	-0.51	6.46	H	318	-0.15	9.81				
2LEA	C13NOESY_@ARO	900	C	64	115.87	134.87	HC	395	6.03	9.10	H	475	0.08	9.35				
2LEA	CBCANH	500	N	128	105.98	130.98	C	256	8.89	70.92	HN	1024	4.65	12.66				
2LEA	CBCAcoNH	600	N	128	103.53	133.53	C	128	8.91	70.90	HN	1024	4.66	12.67				
2LEA	HCCHTOCSY_@ALI	750	C	256	13.28	45.28	HC	300	-0.18	6.26	H	1140	-0.10	6.01				
2LEA	HNCA	500	N	256	97.47	132.47	C	256	38.87	70.86	HN	1024	4.66	12.67				
2LEA	N15HSQC	900	N	512	97.90	132.90	H	1024	4.65	10.65								
2LEA	N15NOESY	900	N	128	105.70	131.10	HN	1024	4.66	11.69	H	512	-0.69	10.01				
2LL8	C13HSQC_@ALI	850	C	449	9.81	79.93	H	478	-1.01	5.99								
2LL8	C13HSQC_@ARO	850	C	256	109.00	141.00	H	342	4.97	9.98								
2LL8	C13NOESY_@ALI	850	C	256	10.33	76.31	HC	478	-1.01	5.99	H	256	-1.22	10.78				
2LL8	C13NOESY_@ARO	850	C	128	111.00	142.00	HC	342	4.99	10.00	H	256	-1.22	10.78				
2LL8	CBCANH	600	N	256	105.58	132.57	C	128	13.52	78.52	HN	315	5.98	11.51				
2LL8	CBCAcoNH	600	N	256	105.58	132.57	C	128	13.52	78.52	HN	315	5.98	11.51				
2LL8	CCHTOCSY_@ALI	600	C1	512	4.50	70.50	C2	256	4.50	70.50	H1	428	-1.04	6.47				
2LL8	CcoNH	600	N	128	105.54	132.54	C	256	12.97	78.97	HN	315	5.98	11.51				
2LL8	HBHAcoNH	850	N	256	105.67	132.66	HN	479	4.99	12.00	H	128	-0.47	6.53				
2LL8	HCCHTOCSY_@ALI	850	C	256	7.22	73.22	HC	256	-0.49	6.51	H	478	-1.02	5.98				
2LL8	HCcoNH_@ALI	600	N	256	104.08	131.08	H	256	-0.71	6.29	HN	315	5.99	11.52				
2LL8	HNCO	600	N	128	105.54	132.54	C	64	165.97	181.97	HN	286	5.97	11.00				
2LL8	HNcoCA	600	N	256	105.57	132.57	C	128	41.01	71.01	HN	314	6.00	11.52				
2LL8	N15HSQC	600	N	512	105.56	132.56	H	285	5.98	11.00								
2LL8	N15NOESY	850	N	128	105.69	132.69	HN	322	5.98	11.01	H	256	-1.22	10.78				
2KPN	C13HSQC_@ALI	600	C	1024	6.24	81.24	H	2048	-2.22	11.76								
2KPN	C13NOESY_@ALI	800	C	512	29.77	53.77	HC	552	-1.01	6.50	H	512	-1.20	10.80				
2KPN	C13NOESY_@ARO	800	C	512	112.76	142.76	HC	512	4.80	11.77	H	512	-1.20	10.80				
2KPN	CBCANH	600	N	256	105.19	132.19	C	512	4.14	79.16	HN	1024	-2.20	11.78				
2KPN	CBCAcoNH	600	N	256	105.16	132.16	C	512	4.24	79.24	HN	1024	-2.20	11.78				
2KPN	CCHTOCSY_@ALI	600	C1	512	31.74	55.74	C2	512	6.24	81.24	H1	550	-1.02	6.49				
2KPN	HBHAcoNH	600	N	256	105.16	132.16	HN	1024	-2.20	11.78	H	512	-1.23	6.77				
2KPN	HCCHCOSY_@ALI	600	C	512	31.74	55.74	HC	512	-1.22	6.78	H	550	-1.01	6.50				
2KPN	HCCHTOCSY_@ALI	600	C	512	31.75	55.76	HC	512	-1.23	6.77	H	550	-1.02	6.49				
2KPN	HNCA	600	N	256	105.16	132.16	C	512	40.63	72.69	HN	1024	-2.20	11.78				
2KPN	HNCO	600	N	256	105.16	132.16	C	256	167.69	189.78	HN	1024	-2.20	11.78				
2KPN	HNcaCO	600	N	256	105.13	132.13	C	256	167.72	189.81	HN	1024	-2.20	11.78				
2KPN	N15HSQC	600	N	2048	101.14	136.14	H	2048	-2.20	11.78								
2KPN	N15NOESY	800	N	256	105.12	132.12	HN	1024	-2.18	11.77	H	512	-1.20	10.80				
2K0M	C13HSQC_@ALI	800	C	512	5.73	85.76	H	2048	-5.23	14.80								
2K0M	C13NOESY_@ALI	800	C	128	33.78	57.78	HC	768	-0.93	6.58	H	214	-1.50	11.04				
2K0M	C13NOESY_@ARO	800	C	128	112.76	142.77	HC	359	5.56	9.07	H	214	-1.51	11.03				
2K0M	CBCANH	800	N	64	104.19	131.19	C	256	4.12	79.19	HN	512	4.79	11.76				
2K0M	CBCAcoNH	800	N	64	104.07	131.06	C	256	4.26	79.22	HN	512	4.79	11.76				
2K0M	CCHTOCSY_@ALI	800	C1	128	29.78	53.77	C2	256	4.26	79.22	H1	551	-1.43	6.08				
2K0M	HBHAcoNH	800	N	128	105.63	132.63	HN	512	4.78	11.76	H	256	1.03	6.04				

PDB	Spectrum name	MHz	L1	Size	Range (ppm)		L2	Size	Range (ppm)		L3	Size	Range (ppm)		L4	Size	Range (ppm)	
2K0M	HCCHCOSY_@ALI	800	C	64	33.73	57.72	HC	128	0.29	6.29	H	551	-1.43	6.08				
2K0M	HCCHTOCSY_@ALI	800	C	128	29.78	53.77	HC	128	0.29	6.29	H	551	-1.43	6.08				
2K0M	HNCA	800	N	64	104.19	131.19	C	128	41.77	71.77	HN	512	4.78	11.76				
2K0M	HNCO	800	N	64	104.19	131.19	C	128	168.81	188.81	HN	512	4.78	11.76				
2K0M	HNcaCO	800	N	64	104.68	130.69	C	128	168.81	188.81	HN	512	4.78	11.76				
2K0M	N15HSQC	800	N	256	100.10	135.10	H	614	5.00	11.00								
2K0M	N15NOESY	800	N	128	104.13	131.13	HN	1024	4.79	14.80	H	256	-2.73	12.28				
2K5V	C13HSQC	600	C	1024	4.23	79.23	H	2048	-2.19	11.79								
2K5V	C13NOESY_@ALI	800	C	512	19.89	89.89	HC	551	-1.01	6.49	H	440	-1.02	11.01				
2K5V	C13NOESY_@ARO	800	C	256	112.74	142.74	HC	512	4.81	11.78	H	512	-0.69	10.31				
2K5V	CBCANH	600	N	256	103.17	133.18	C	512	4.16	79.16	HN	512	4.80	11.79				
2K5V	CBCAcoNH	600	N	256	103.14	133.14	C	512	4.25	79.25	HN	512	4.82	11.80				
2K5V	CCHTOCSY_@ALI	600	C1	512	31.75	55.75	C2	512	6.29	81.29	H1	550	-1.01	6.50				
2K5V	HBHAcoNH	600	N	256	103.03	133.03	HN	512	4.81	11.80	H	512	0.31	6.31				
2K5V	HCCHCOSY_@ALI	600	C	256	31.72	55.72	HC	512	-0.07	6.43	H	551	-1.02	6.51				
2K5V	HCCHTOCSY_@ALI	600	C	512	31.75	55.75	HC	512	-0.08	6.42	H	551	-1.02	6.51				
2K5V	HNCA	600	N	256	103.17	133.18	C	512	40.69	72.69	HN	512	4.80	11.79				
2K5V	HNCO	600	N	256	103.14	133.14	C	512	167.73	189.81	HN	512	4.83	11.82				
2K5V	HNcaCO	600	N	256	103.17	133.18	C	512	167.69	189.78	HN	512	4.81	11.80				
2K5V	HNcoCA	600	N	256	103.17	133.18	C	512	40.77	72.77	HN	512	4.81	11.80				
2K5V	N15HSQC	600	N	1024	103.13	133.13	H	1024	4.80	11.79								
2K5V	N15NOESY	800	N	256	104.23	135.06	HN	512	4.81	11.79	H	440	-1.03	11.00				
2MQL	C13HSQC_@ALI	900	C	1024	10.49	75.50	H	2560	-0.30	5.94								
2MQL	C13HSQC_@ARO	750	C	1024	116.01	139.01	H	1280	5.42	9.17								
2MQL	C13NOESY_@ALI	900	C	256	11.49	47.99	HC	2463	-0.14	5.87	H	473	-0.22	9.94				
2MQL	C13NOESY_@ARO	900	C	128	115.01	135.01	HC	509	6.10	8.84	H	480	-0.27	10.05				
2MQL	CcoNH	750	N	256	104.59	127.09	C	248	8.96	68.05	HN	1477	6.05	10.00				
2MQL	HCcoNH_@ALI	750	N	256	104.59	127.09	H	281	-0.24	5.80	HN	353	6.09	9.87				
2MQL	HNCA	500	N	256	100.18	132.98	C	128	43.00	69.00	HN	1024	4.72	10.21				
2MQL	N15HSQC	900	N	949	100.40	133.76	H	1525	5.92	10.03								
2MQL	N15NOESY_@ALI	900	N	128	106.50	127.50	HN	1453	5.91	10.16	H	441	-0.28	10.06				
2K75	C13HSQC_@ALI	600	C	1024	3.04	83.04	H	308	-0.04	5.98								
2K75	C13HSQC_@ARO	600	C	256	112.51	137.51	H	206	5.97	10.00								
2K75	C13NOESY_@ALI	850	C	256	6.69	84.66	HC	479	-1.02	6.00	H	256	-1.33	10.92				
2K75	CBCANH	600	N	128	102.01	132.01	C	128	13.52	78.52	HN	309	4.97	11.01				
2K75	CBCAcoNH	600	N	128	102.02	132.02	C	128	13.45	78.45	HN	309	4.97	11.01				
2K75	CCNOESY_@ALI	600	C1	64	13.26	33.95	C2	64	13.26	33.95	H1	1024	-3.69	9.65	H2	128	-0.52	6.48
2K75	CcoNH	600	N	128	102.02	132.02	C	128	15.98	75.98	HN	258	5.97	11.01				
2K75	HBHAcoNH	600	N	128	102.02	132.02	HN	232	5.97	10.50	H	128	0.02	6.02				
2K75	HCCHCOSY_@ALI	600	C	512	8.02	78.02	HC	102	-1.04	5.97	H	385	-1.01	6.50				
2K75	HCCHTOCSY_@ALI	600	C	512	7.97	77.98	HC	205	-1.04	6.00	H	385	-1.02	6.50				
2K75	HCcoNH_@ALI	600	N	128	102.02	132.02	H	128	-0.73	6.27	HN	232	5.97	10.50				
2K75	HNCA	600	N	128	102.02	132.02	C	128	40.90	70.90	HN	309	4.97	11.01				
2K75	HNCO	600	N	128	102.02	132.02	C	64	165.50	182.50	HN	309	4.98	11.01				
2K75	N15HSQC	850	N	256	102.06	132.14	H	308	5.99	10.50								
2K75	N15NOESY	850	N	128	102.06	132.13	HN	376	4.99	10.50	H	256	-1.08	10.68				
2LTM	C13HSQC_@ALI	800	C	512	6.52	76.52	H	2048	-2.17	11.78								
2LTM	C13HSQC_@ARO	800	C	128	112.55	142.55	H	1024	4.80	11.78								
2LTM	C13NOESY_@ALI	800	C	128	19.50	89.50	HC	604	-1.63	6.60	H	472	-1.65	11.26				
2LTM	C13NOESY_@ARO	800	C	64	112.78	142.78	HC	224	5.32	8.37	H	256	-1.69	11.31				
2LTM	CBCANH	800	N	64	102.63	133.63	C	256	3.98	78.98	HN	512	4.79	11.77				
2LTM	CBCAcoNH	800	N	64	102.63	133.63	C	256	4.13	79.13	HN	512	4.79	11.77				
2LTM	CCHTOCSY_@ALI	800	C1	64	29.58	53.58	C2	128	3.98	78.98	H1	1024	-2.17	11.78				
2LTM	HBHAcoNH	800	N	64	102.90	132.90	HN	512	4.80	11.78	H	256	-0.46	6.54				
2LTM	HNCO	800	N	64	102.63	133.63	C	64	167.60	189.60	HN	512	4.79	11.77				
2LTM	N15HSQC	800	N	512	102.51	133.51	H	1024	4.80	11.78								
2LTM	N15NOESY	800	N	128	102.51	133.51	HN	374	6.00	11.09	H	512	-2.19	11.80				
2KOB	C13HSQC_@ALI	850	C	512	5.77	85.74	H	515	-2.00	7.01								
2KOB	C13NOESY_@ALI	850	C	256	8.45	71.41	HC	344	-0.53	5.49	H	256	-2.22	11.79				
2KOB	C13NOESY_@ARO	600	C	64	114.00	142.00	HC	228	4.99	8.99	H	256	-2.22	11.78				
2KOB	CBCANH	600	N	256	105.92	129.91	C	128	13.50	78.50	HN	314	5.01	10.53				
2KOB	CBCAcoNH	600	N	256	105.92	129.91	C	128	13.50	78.50	HN	314	5.01	10.53				
2KOB	CCHTOCSY_@ALI	600	C1	512	8.57	71.58	C2	256	8.50	71.50	H1	371	-0.50	6.01				
2KOB	CCNOESY_@ALI	600	C1	64	13.29	33.99	C2	64	13.29	33.99	H1	1024	-3.70	9.64	H2	128	-0.53	6.47
2KOB	HBHAcoNH	600	N	128	105.94	129.94	HN	314	5.01	10.53	H	256	-0.20	5.80				
2KOB	HCCHCOSY_@ALI	850	C	512	6.74	76.77	HC	344	-0.53	5.49	H	256	-0.46	6.54				
2KOB	HCCHCOSY_@ALI_2	850	C	512	6.74	76.77	HC	256	-0.46	6.54	H	344	-0.53	5.49				
2KOB	HCCHTOCSY_@ALI	600	C	256	8.50	71.50	HC	122	-0.22	5.50	H	343	-0.50	5.52				
2KOB	HNCA	600	N	128	105.94	129.94	C	64	41.00	71.00	HN	286	6.00	11.02				
2KOB	HNCO	600	N	256	105.92	129.92	C	64	166.04	181.96	HN	342	5.01	11.02				
2KOB	HNcoCA	600	N	128	105.94	129.94	C	64	41.00	71.00	HN	342	5.01	11.02				
2KOB	N15HSQC	850	N	256	104.00	134.00	H	400	5.01	12.02								
2KOB	N15NOESY	850	N	128	106.00	130.00	HN	400	5.01	12.02	H	256	-1.71	11.29				
2KHD	C13HSQC_@ALI	850	C	512	5.66	85.66	H	615	-2.01	7.00								
2KHD	C13HSQC_@ARO	850	C	256	112.80	142.80	H	240	5.49	9.01								
2KHD	C13NOESY_@ALI	850	C	256	10.44	79.24	HC	479	-1.01	6.01	H	256	-0.45	10.05				
2KHD	C13NOESY_@ARO	850	C	128	116.80	138.80	HC	254	5.28	9.01	H	256	-0.45	10.05				
2KHD	CBCANH	850	N	256	104.09	133.11	C	128	9.73	79.56	HN	376	5.49	11.00				
2KHD	CBCAcoNH	850	N	256	104.09	133.11	C	128	10.39	80.21	HN	376	5.49	11.00				
2KHD	CCHTOCSY_@ALI	600	C1	256	3.00	83.00	C2	256	3.00	83.00	H1	385	-1.02	6.50				
2KHD	CcoNH	600	N	256	106.05	135.06	C	128	13.49	78.51	HN	257	5.96	10.99				
2KHD	HBHAcoNH	600	N	256	104.00	133.00	HN	258	5.97	11.01	H	128	0.02	6.02				
2KHD	HCCHCOSY_@ALI	600	C	256	30.94	54.94	HC	113	-1.09	5.97	H	460	-1.02	6.48				
2KHD	HCCHTOCSY_@ALI	600	C	256	30.88	54.88	HC	225	-1.06	5.97	H	460	-1.03	6.48				
2KHD	HCcoNH_@ALI	600	N	256	104.00	133.00	H	128	-0.51	6.50	HN	206	5.97	9.99				
2KHD	HNCA	600	N	256	104.00	133.00	C	128	40.93	70.93	HN	206	5.97	9.99				

PDB	Spectrum name	MHz	L1	Size	Range (ppm)	L2	Size	Range (ppm)	L3	Size	Range (ppm)	L4	Size	Range (ppm)
2KHD	HNCO	850	N	128	104.09 133.11	C	128	166.69 183.70	HN	274	5.99 10.01			
2KHD	HNcoCA	600	N	256	104.00 133.00	C	128	41.00 71.00	HN	206	5.97 9.99			
2KHD	N15HSQC	750	N	256	104.03 133.03	H	301	5.28 9.99						
2KHD	N15NOESY	850	N	128	104.09 133.11	HN	329	5.18 10.00	H	256	-0.27 9.87			
2RN7	C13HSQC_@ALI	750	C	481	-0.20 74.95	H	660	-2.02 6.99						
2RN7	C13NOESY_@ALI	750	C	256	30.93 54.93	HC	416	-1.01 5.50	H	256	-1.00 10.60			
2RN7	C13NOESY_@ARO	750	C	128	109.93 139.93	HC	257	4.97 8.99	H	256	-1.01 10.59			
2RN7	CBCANH	600	N	256	101.11 134.89	C	128	11.00 81.00	HN	246	5.99 10.00			
2RN7	CBCAcoNH	600	N	256	101.11 134.89	C	128	11.00 81.00	HN	246	5.99 10.00			
2RN7	CCHTOCSY_@ALI	600	C1	256	30.94 54.94	C2	256	5.47 80.47	H1	461	-1.03 6.49			
2RN7	CcoNH	600	N	256	101.11 134.89	C	128	2.64 77.64	HN	369	4.97 10.99			
2RN7	HBHAcoNH	600	N	256	101.11 134.89	HN	430	4.97 11.99	H	128	-1.20 6.80			
2RN7	HCCHCOSY_@ALI	750	C	256	30.92 54.92	HC	241	-1.04 6.49	H	576	-1.01 6.49			
2RN7	HNCO	600	N	256	101.11 134.89	C	128	167.36 180.64	HN	460	4.49 11.99			
2RN7	N15HSQC	600	N	256	101.11 134.89	H	614	5.99 11.00						
2RN7	N15NOESY	750	N	256	101.08 134.84	HN	257	5.97 11.00	H	256	-0.91 10.51			
2LXU	C13HSQC_@ALI	850	C	1024	5.72 85.69	H	410	-0.01 6.00						
2LXU	C13HSQC_@ARO	850	C	512	113.71 143.72	H	257	5.49 9.01						
2LXU	C13NOESY_@ALI	850	C	256	11.74 76.72	HC	513	-0.01 6.00	H	256	-0.97 10.53			
2LXU	C13NOESY_@ARO	850	C	128	113.22 139.22	HC	206	5.99 8.40	H	256	-0.97 10.53			
2LXU	CBCANH	600	N	128	105.98 127.98	C	128	13.32 78.32	HN	257	5.97 10.49			
2LXU	CBCAcoNH	600	N	128	105.98 127.98	C	128	13.46 78.46	HN	257	5.97 10.49			
2LXU	CCHTOCSY_@ALI	600	C1	256	7.97 77.98	C2	256	7.98 77.98	H1	427	-1.03 6.48			
2LXU	CCNOESY_@ALI	600	C1	64	13.29 33.99	C2	64	13.29 33.99	H1	1024	-3.72 9.62	H2	128	-0.55 6.45
2LXU	CcoNH	600	N	256	105.99 127.99	C	128	10.97 80.97	HN	257	5.96 10.49			
2LXU	HBHAcoNH	600	N	128	105.99 127.99	HN	258	5.96 10.49	H	128	-0.25 5.75			
2LXU	HCCHTOCSY_@ALI	600	C	256	7.82 77.82	HC	128	-0.76 6.24	H	541	-2.03 7.49			
2LXU	HNCO	600	N	256	105.98 127.98	C	128	169.00 182.92	HN	257	5.97 10.50			
2LXU	N15HSQC	850	N	1024	106.07 128.07	H	330	5.98 10.50						
2LXU	N15NOESY	850	N	128	106.07 128.07	HN	385	5.99 10.50	H	512	-0.97 10.53			
2KIF	C13HSQC_@ALI	800	C	1024	6.78 76.78	H	2048	-2.16 11.79						
2KIF	C13NOESY_@ALI	800	C	512	19.71 89.71	HC	551	-1.01 6.49	H	440	-0.54 11.49			
2KIF	C13NOESY_@ARO	800	C	256	112.78 142.78	HC	512	4.81 11.78	H	512	-1.19 10.81			
2KIF	N15HSQC	800	N	1024	100.64 135.63	H	2048	-2.16 11.79						
2KIF	N15NOESY	800	N	256	104.22 135.05	HN	512	4.81 11.79	H	440	-0.52 11.51			
2KBN	C13HSQC_@ALI	850	C	475	5.75 79.97	H	513	-1.02 6.50						
2KBN	C13HSQC_@ARO	600	C	512	110.00 140.00	H	257	4.98 10.00						
2KBN	C13NOESY_@ALI	850	C	256	6.72 84.69	HC	479	-1.02 6.00	H	256	-1.34 10.92			
2KBN	C13NOESY_@ARO	850	C	128	112.30 140.31	HC	206	5.99 9.01	H	256	-1.45 11.05			
2KBN	CBCANH	600	N	128	103.50 131.50	C	128	13.50 78.50	HN	258	5.97 11.01			
2KBN	CBCAcoNH	600	N	128	103.50 131.50	C	128	13.50 78.50	HN	232	5.97 10.50			
2KBN	CCNOESY_@ALI	600	C1	64	13.29 33.99	C2	64	13.29 33.99	H1	1024	-3.68 9.65	H2	128	-0.51 6.48
2KBN	CcoNH	600	N	128	103.50 131.50	C	128	13.19 73.19	HN	232	5.96 10.49			
2KBN	HBHAcoNH	600	N	128	103.53 131.53	HN	232	5.96 10.49	H	128	0.02 6.02			
2KBN	HCCHTOCSY_@ALI	600	C	512	8.00 78.00	HC	205	-1.04 6.00	H	385	-1.02 6.50			
2KBN	HCcoNH_@ALI	600	N	128	103.50 131.50	H	128	-0.72 6.28	HN	232	5.96 10.49			
2KBN	HNCA	600	N	128	103.50 131.50	C	64	40.86 70.86	HN	258	5.96 11.00			
2KBN	HNCO	600	N	128	103.50 131.50	C	64	165.50 182.50	HN	258	5.97 11.01			
2KBN	N15HSQC	850	N	512	102.61 131.63	H	308	5.99 10.50						
2KBN	N15NOESY	850	N	128	102.61 131.63	HN	308	5.99 10.50	H	512	-1.33 10.93			
2MK2	C13HSQC_@ALI	850	C	512	3.27 88.33	H	440	-1.21 6.50						
2MK2	C13HSQC_@ARO	850	C	256	110.03 140.04	H	274	5.48 9.50						
2MK2	C13NOESY_@ALI	850	C	256	13.01 78.99	HC	458	-1.03 6.99	H	256	-1.24 10.77			
2MK2	C13NOESY_@ARO	850	C	256	111.20 141.20	HC	274	5.48 9.50	H	256	-1.22 10.78			
2MK2	CBCANH	600	N	128	101.00 133.00	C	128	13.50 78.50	HN	342	5.47 11.49			
2MK2	CBCAcoNH	600	N	128	101.00 133.00	C	128	13.50 78.50	HN	370	5.47 11.99			
2MK2	CcoNH	600	N	128	101.00 133.00	C	256	11.00 81.00	HN	370	5.48 11.99			
2MK2	HBHAcoNH	600	N	256	101.00 133.00	HN	314	5.47 10.99	H	256	0.01 7.01			
2MK2	HCCHTOCSY_@ALI	600	C	256	10.08 76.08	HC	238	-1.02 5.48	H	584	-2.02 7.48			
2MK2	HNCA	600	N	128	101.00 133.00	C	64	38.66 68.66	HN	314	5.48 11.00			
2MK2	HNCO	600	N	256	101.00 133.00	C	128	169.01 182.93	HN	370	5.48 11.99			
2MK2	HNcoCA	600	N	256	101.00 133.00	C	64	41.00 71.00	HN	285	5.97 10.99			
2MK2	N15HSQC	600	N	1024	101.00 133.00	H	445	5.48 12.00						
2MK2	N15NOESY	850	N	128	101.07 133.07	HN	286	5.99 11.00	H	256	-0.94 11.06			
2K50	C13HSQC_@ALI	800	C	512	8.26 83.22	H	896	-0.98 6.03						
2K50	C13NOESY_@ALI	800	C	128	29.77 53.77	HC	552	-1.51 6.00	H	256	-0.69 10.30			
2K50	C13NOESY_@ARO	800	C	128	112.77 142.77	HC	221	6.00 9.01	H	256	-0.69 10.30			
2K50	CBCANH	600	N	128	102.12 132.12	C	128	4.08 79.09	HN	512	4.84 11.83			
2K50	CBCAcoNH	600	N	64	102.12 132.12	C	128	4.26 79.26	HN	512	4.84 11.83			
2K50	CCHTOCSY_@ALI	600	C1	128	31.76 55.76	C2	256	5.88 80.88	H1	513	-1.02 5.99			
2K50	HBHAcoNH	600	N	64	103.12 133.12	HN	512	4.83 11.82	H	256	0.33 6.33			
2K50	HCCHTOCSY_@ALI	600	C	128	31.76 55.76	HC	128	-0.46 6.04	H	513	-1.02 5.98			
2K50	HNCA	600	N	128	103.05 133.05	C	128	40.73 72.79	HN	512	4.83 11.82			
2K50	HNCO	600	N	128	104.23 134.23	C	128	167.72 189.80	HN	512	4.83 11.82			
2K50	HNcaCO	600	N	128	105.23 135.23	C	128	167.72 189.80	HN	512	4.83 11.82			
2K50	HNcoCA	600	N	128	103.12 133.12	C	128	40.68 72.69	HN	512	4.83 11.82			
2K50	N15HSQC	800	N	256	100.63 135.63	H	1024	4.81 14.82						
2K50	N15NOESY	800	N	64	103.13 133.13	HN	512	4.81 11.78	H	512	-2.69 12.31			
2KL5	C13HSQC_@ALI	800	C	512	6.55 76.55	H	2048	-2.21 11.74						
2KL5	C13HSQC_@ARO	800	C	256	112.66 142.67	H	1024	4.77 11.75						
2KL5	C13NOESY_@ALI	800	C	128	34.54 74.54	HC	524	-1.12 6.02	H	512	-2.23 11.77			
2KL5	C13NOESY_@ARO	800	C	64	112.63 142.63	HC	304	4.86 8.43	H	256	-1.23 10.78			
2KL5	CBCANH	600	N	64	101.43 134.34	C	128	13.27 83.27	HN	512	4.76 11.43			
2KL5	CBCAcoNH	600	N	64	101.43 134.34	C	128	13.29 83.29	HN	512	4.74 11.41			
2KL5	N15HSQC	800	N	512	101.59 134.59	H	1024	4.76 11.74						

PDB	Spectrum name	MHz	L1	Size	Range (ppm)		L2	Size	Range (ppm)		L3	Size	Range (ppm)		L4	Size	Range (ppm)	
2KL5	N15NOESY	800	N	128	104.13	135.04	HN	503	4.82	11.67	H	463	-0.89	11.77				
2LTA	C13NOESY_@ALI	800	C	128	19.36	89.36	HC	448	-0.37	5.73	H	409	-0.64	10.55				
2LTA	C13NOESY_@ARO	800	C	63	112.64	142.18	HC	101	6.36	7.74	H	234	-0.87	11.01				
2LTA	CBCANH	600	N	128	103.01	132.63	C	128	13.24	83.25	HN	512	4.79	11.46				
2LTA	CBCAcoNH	600	N	128	103.01	132.63	C	128	13.27	83.28	HN	512	4.79	11.46				
2LTA	CCHTOCSY_@ALI	800	C1	64	29.41	53.41	C2	128	4.09	79.09	H1	1024	-2.17	11.78				
2LTA	CcoNH	600	N	64	103.00	132.62	C	128	8.58	88.17	HN	512	4.79	11.46				
2LTA	HBHAcoNH	800	N	64	103.04	133.04	HN	512	4.81	11.79	H	256	-0.44	6.56				
2LTA	HNCO	600	N	64	103.02	132.64	C	64	167.78	185.02	HN	512	4.79	11.46				
2LTA	N15HSQC	600	N	512	103.02	132.64	H	1024	4.79	11.46								
2LTA	N15NOESY	800	N	128	102.25	133.43	HN	287	6.37	10.27	H	410	-0.66	10.55				
2KIW	C13HSQC_@ALI	850	C	1024	3.25	88.25	H	615	-2.01	7.00								
2KIW	C13HSQC_@ARO	600	C	512	110.00	140.00	H	228	5.98	9.99								
2KIW	C13NOESY_@ALI	850	C	256	6.72	76.72	HC	401	-1.01	6.01	H	256	-1.08	10.68				
2KIW	C13NOESY_@ARO	850	C	128	111.30	141.30	HC	201	5.48	9.00	H	512	-1.08	10.68				
2KIW	CBCANH	600	N	256	103.00	131.00	C	128	11.00	81.00	HN	400	4.98	12.01				
2KIW	CBCAcoNH	600	N	256	103.00	131.00	C	128	11.00	81.00	HN	400	4.98	12.00				
2KIW	CCHTOCSY_@ALI	600	C1	256	8.16	78.17	C2	256	8.16	78.16	H1	428	-1.01	6.50				
2KIW	CCNOESY_@ALI	600	C1	64	13.29	33.99	C2	32	13.29	33.99	H1	128	-0.53	6.47	H2	1024	-3.70	9.64
2KIW	CcoNH	600	N	256	106.75	131.25	C	128	16.00	76.00	HN	400	4.98	12.01				
2KIW	HBHAcoNH	600	N	256	106.75	131.25	HN	258	6.00	10.53	H	128	-0.21	5.79				
2KIW	HCCHCOSY_@ALI	850	C	256	6.72	76.72	HC	256	-0.39	6.61	H	401	-0.96	6.06				
2KIW	HCCHTOCSY_@ALI	600	C	128	7.83	77.84	HC	127	-0.93	6.51	H	542	-1.99	7.53				
2KIW	HCcoNH_@ALI	600	N	256	106.75	131.25	H	128	-0.93	6.57	HN	229	6.00	10.02				
2KIW	HNCA	600	N	256	103.00	131.00	C	128	40.93	70.93	HN	400	4.98	12.00				
2KIW	HNCO	600	N	128	103.00	131.00	C	64	167.37	180.63	HN	343	4.98	11.00				
2KIW	HNcoCA	600	N	256	103.00	131.00	C	128	41.00	71.00	HN	400	4.98	12.01				
2KIW	N15HSQC	600	N	1024	106.74	131.24	H	400	4.98	12.01								
2KIW	N15NOESY	850	N	256	106.84	131.34	HN	478	4.99	11.99	H	256	-1.33	10.93				
2LVB	C13NOESY_@ALI	800	C	128	19.73	89.73	HC	524	-1.08	6.05	H	512	-2.20	11.80				
2LVB	C13NOESY_@ARO	800	C	64	112.72	142.72	HC	264	5.45	9.05	H	256	-1.70	11.30				
2LVB	CBCANH	800	N	64	102.54	133.54	C	256	4.08	79.08	HN	512	4.80	11.77				
2LVB	CBCAcoNH	800	N	64	102.54	133.54	C	256	4.16	79.16	HN	512	4.80	11.77				
2LVB	CCHTOCSY_@ALI	800	C1	64	29.77	53.77	C2	128	4.31	79.31	H1	1024	-2.17	11.77				
2LVB	HNCO	800	N	64	102.54	133.54	C	64	167.61	189.61	HN	512	4.80	11.77				
2LVB	N15HSQC	800	N	512	103.07	133.07	H	1024	4.80	11.77								
2LVB	N15NOESY	800	N	128	102.29	133.47	HN	288	6.41	10.33	H	431	-1.16	10.62				
2LND	C13HSQC_@ALI	800	C	512	76.65	146.65	H	2048	-2.18	11.77								
2LND	C13HSQC_@ARO	800	C	128	112.75	142.75	H	1024	4.81	11.78								
2LND	C13NOESY_@ALI	800	C	128	19.72	89.72	HC	564	-1.36	6.32	H	512	-2.20	11.80				
2LND	C13NOESY_@ARO	800	C	64	112.73	142.73	HC	124	6.27	7.96	H	256	-1.70	11.30				
2LND	CBCANH	800	N	64	103.60	132.60	C	256	4.11	79.11	HN	512	4.80	11.78				
2LND	CBCAcoNH	800	N	64	103.60	132.60	C	256	4.17	79.17	HN	512	4.80	11.77				
2LND	CCHTOCSY_@ALI	800	C1	64	29.73	53.73	C2	128	4.28	79.28	H1	1024	-2.17	11.77				
2LND	HBHAcoNH	800	N	64	103.60	132.60	HN	512	4.80	11.78	H	256	-0.45	6.55				
2LND	HNCO	800	N	64	103.60	132.60	C	64	167.56	189.56	HN	512	4.80	11.78				
2LND	N15HSQC	800	N	512	103.60	132.60	H	1024	4.80	11.77								
2LND	N15NOESY	800	N	128	102.50	133.68	HN	295	6.39	10.41	H	437	-1.25	10.70				
1WQU	C13HSQC	800	C	512	5.85	45.35	H	1322	-1.00	8.00								
1WQU	C13NOESY	800	C	128	5.83	45.34	HC	1322	-1.00	8.00	H	800	-2.28	11.67				
1WQU	CBCANH	600	N	128	101.05	133.95	C	256	-0.39	74.59	HN	367	5.19	10.20				
1WQU	CBCAcoNH	600	N	128	101.05	133.95	C	256	0.02	75.00	HN	367	5.19	10.20				
1WQU	CcoNH	600	N	128	101.05	133.95	C	256	-0.15	74.83	HN	367	5.19	10.20				
1WQU	HBHAcoNH	600	N	128	101.05	133.95	HN	367	5.19	10.20	H	256	-2.29	11.69				
1WQU	HCCHCOSY_@ALI	600	C	128	-0.06	74.91	HC	276	-1.04	6.50	H	550	-1.00	6.50				
1WQU	HCCHCOSY_@ARO	600	C	128	108.94	139.06	HC	112	5.79	8.00	H	223	5.80	8.00				
1WQU	HCCHTOCSY_@ALI	600	C	128	-0.12	74.85	HC	276	-1.04	6.50	H	550	-1.01	6.50				
1WQU	HCcoNH_@ALI	600	N	128	101.05	133.95	H	256	-0.86	10.25	HN	367	5.18	10.20				
1WQU	HNCA	600	N	128	101.05	133.95	C	256	35.65	67.73	HN	367	5.19	10.20				
1WQU	HNCO	600	N	128	101.05	133.95	C	256	161.50	183.59	HN	367	5.20	10.21				
1WQU	HNcaCO	600	N	128	101.05	133.95	C	256	161.50	183.59	HN	367	5.19	10.20				
1WQU	HNcoCA	600	N	128	101.05	133.95	C	256	35.66	67.75	HN	367	5.19	10.20				
1WQU	N15HSQC	800	N	512	101.05	133.94	H	742	5.20	10.25								
1WQU	N15NOESY	800	N	128	101.05	133.95	HN	735	5.20	10.20	H	800	-2.27	11.68				
2KL6	C13HSQC_@ALI	800	C	1024	6.77	76.77	H	2048	-2.17	11.78								
2KL6	C13HSQC_@ARO	800	C	1024	107.26	142.26	H	2048	-2.17	11.78								
2KL6	C13NOESY	800	C	256	29.74	53.74	HC	1024	-2.18	11.77	H	512	-1.20	10.80				
2KL6	C13NOESY_@ARO	800	C	256	112.80	142.80	HC	512	4.80	11.77	H	512	-1.20	10.80				
2KL6	CBCANH	600	N	256	100.67	135.67	C	512	4.17	79.19	HN	512	4.81	11.80				
2KL6	CBCAcoNH	600	N	256	100.63	135.63	C	512	4.18	79.18	HN	512	4.80	11.79				
2KL6	CCHTOCSY_@ALI	600	C1	512	31.77	55.77	C2	512	6.27	81.27	H1	587	-1.52	6.50				
2KL6	HBHAcoNH	600	N	256	100.63	135.63	HN	507	4.80	11.79	H	512	0.31	6.31				
2KL6	HCCHCOSY_@ALI	600	C	512	31.76	55.76	HC	512	-0.44	6.56	H	587	-1.52	6.50				
2KL6	HCCHTOCSY_@ALI	600	C	512	31.77	55.77	HC	512	-0.44	6.56	H	587	-1.52	6.50				
2KL6	HNCA	600	N	256	100.67	135.67	C	512	40.70	72.76	HN	512	4.80	11.79				
2KL6	HNCO	600	N	256	100.63	135.63	C	512	167.73	189.81	HN	512	4.80	11.79				
2KL6	HNcaCO	600	N	256	100.71	135.71	C	256	167.75	189.84	HN	512	4.80	11.79				
2KL6	HNcoCA	600	N	256	100.71	135.71	C	512	40.74	72.76	HN	512	4.81	11.80				
2KL6	N15HSQC	800	N	1024	100.63	135.63	H	2048	-2.17	11.78								
2KL6	N15NOESY	800	N	256	105.09	132.09	HN	512	4.80	11.78	H	512	-1.20	10.80				
6GT7	C13HSQC_@ALI	750	C	239	6.92	72.22	H	814	-1.17	5.20								
6GT7	C13HSQC_@ARO	900	C	1024	100.03	150.05	H	1024	4.70	12.69								
6GT7	C13NOESY_@ALI	700	C	128	8.49	47.51	HC	804	-0.98	5.28	H	355	-0.80	10.30				
6GT7	C13NOESY_@ARO	900	C	128	110.78	136.82	HC	1024	4.70	12.69	H	512	-3.29	12.68				
6GT7	CBCANH	750	N	256	96.99	134.99	C	256	12.01	73.94	HN	1024	4.70	12.71				

PDB	Spectrum name	MHz	L1	Size	Range (ppm)		L2	Size	Range (ppm)		L3	Size	Range (ppm)		L4	Size	Range (ppm)	
6GT7	CBCAcoNH	750	N	256	96.99	134.99	C	512	12.06	73.99	HN	1024	4.69	12.70				
6GT7	CcoNH	750	N	256	96.99	134.99	C	512	5.07	71.01	HN	512	4.68	12.69				
6GT7	HNCA	750	N	256	96.99	134.99	C	128	38.02	70.03	HN	1024	4.70	12.71				
6GT7	HNcoCA	750	N	256	96.97	134.97	C	128	38.02	70.03	HN	1024	4.69	12.70				
6GT7	N15HSQC	700	N	1024	96.93	134.92	H	1024	4.70	12.67								
6GT7	N15NOESY	750	N	128	96.92	134.91	HN	1024	4.68	12.70	H	512	-3.31	12.68				
2JN8	C13HSQC_@ALI	500	C	1024	-4.93	75.07	H	2048	-4.17	13.84								
2JN8	C13HSQC_@ARO	750	C	512	110.13	140.13	H	1024	-4.17	13.87								
2JN8	C13NOESY_@ALI	750	C	256	31.15	55.15	HC	398	-0.99	6.02	H	512	-0.89	10.61				
2JN8	C13NOESY_@ARO	750	C	256	110.12	140.12	HC	512	4.84	13.87	H	512	-0.91	10.60				
2JN8	CBCAcoNH	600	N	512	101.71	134.60	C	512	4.42	79.38	HN	512	4.88	12.37				
2JN8	CCHTOCSY_@ALI	500	C1	256	23.07	47.07	C2	256	-2.46	72.55	H1	399	-0.95	6.06				
2JN8	CcoNH	600	N	512	99.85	136.05	C	512	11.04	83.98	HN	512	4.83	11.09				
2JN8	HBHAcoNH	600	N	512	101.69	134.58	HN	512	4.85	11.52	H	512	-0.77	6.73				
2JN8	HCCHCOSY_@ALI	600	C	512	2.67	82.24	HC	512	-1.40	11.11	H	616	-1.00	6.52				
2JN8	HCCHTOCSY_@ALI	500	C	256	31.06	55.06	HC	512	0.05	8.05	H	399	-0.95	6.06				
2JN8	HCCHTOCSY_@ARO	600	C	512	99.83	152.87	HC	512	-1.41	11.09	H	512	4.83	10.67				
2JN8	HCcoNH	600	N	512	101.50	137.69	H	512	-0.58	10.26	HN	512	4.84	11.09				
2JN8	HNCA	600	N	512	101.48	137.67	C	512	39.30	75.77	HN	512	4.84	11.09				
2JN8	HNCO	600	N	512	97.36	138.50	C	512	167.15	183.72	HN	512	4.84	11.51				
2JN8	HNcaCO	500	N	256	104.88	131.19	C	512	166.07	181.98	HN	512	4.83	13.83				
2JN8	HNcoCA	600	N	512	101.48	137.67	C	512	39.28	75.75	HN	512	4.84	11.10				
2JN8	N15HSQC	500	N	1024	104.87	131.19	H	1024	4.83	13.83								
2JN8	N15NOESY	750	N	256	102.06	132.06	HN	512	4.83	13.86	H	512	-1.16	10.84				
2K5D	C13HSQC_@ALI	800	C	1024	5.39	80.35	H	2048	-2.14	11.81								
2K5D	C13HSQC_@ARO	800	C	1024	112.81	142.82	H	1024	4.84	11.81								
2K5D	C13NOESY_@ALI	800	C	512	19.89	89.89	HC	551	-1.02	6.48	H	440	-1.04	10.99				
2K5D	C13NOESY_@ARO	800	C	256	112.80	142.81	HC	512	4.84	11.81	H	512	-0.91	10.59				
2K5D	CBCANH	800	N	256	104.17	131.16	C	512	4.27	79.34	HN	512	4.85	11.82				
2K5D	CBCAcoNH	800	N	256	104.17	131.16	C	512	4.41	79.37	HN	512	4.84	11.81				
2K5D	CCHTOCSY_@ALI	800	C1	512	29.79	53.79	C2	512	4.32	79.28	H1	552	-1.02	6.50				
2K5D	HBHAcoNH	800	N	256	104.14	131.13	HN	512	4.84	11.81	H	512	1.09	6.09				
2K5D	HCCHTOCSY_@ALI	800	C	256	29.80	53.80	HC	512	0.34	6.34	H	552	-1.02	6.50				
2K5D	HNCA	800	N	256	104.20	131.19	C	512	41.81	71.80	HN	512	4.84	11.81				
2K5D	HNCO	800	N	256	104.20	131.19	C	256	168.78	188.78	HN	512	4.85	11.82				
2K5D	HNcaCO	800	N	256	104.17	131.16	C	256	168.83	188.83	HN	512	4.84	11.81				
2K5D	N15HSQC	800	N	1024	103.15	133.15	H	1024	4.84	11.82								
2K5D	N15NOESY	800	N	256	104.23	135.06	HN	504	4.95	11.82	H	440	-1.03	11.00				
2KD1	C13HSQC_@ALI	800	C	512	6.00	81.00	H	666	-0.50	6.01								
2KD1	C13NOESY_@ALI	800	C	128	31.53	55.53	HC	478	-0.50	6.01	H	422	-0.53	11.01				
2KD1	C13NOESY_@ARO	800	C	128	112.76	142.76	HC	192	5.99	8.61	H	512	-2.21	11.79				
2KD1	CBCANH	600	N	128	103.59	130.59	C	128	3.87	78.89	HN	512	4.78	11.77				
2KD1	CBCAcoNH	600	N	128	103.59	130.59	C	128	3.88	78.88	HN	512	4.78	11.76				
2KD1	CCHTOCSY_@ALI	600	C1	128	31.51	55.51	C2	256	5.97	80.97	H1	513	-1.00	6.00				
2KD1	HBHAcoNH	600	N	128	103.59	130.59	HN	512	4.78	11.76	H	128	0.28	6.28				
2KD1	HCCHCOSY_@ALI	600	C	64	31.51	55.51	HC	128	-0.40	6.10	H	1024	-2.21	11.77				
2KD1	HCCHTOCSY_@ALI	600	C	128	31.51	55.51	HC	128	-0.41	6.09	H	513	-0.99	6.01				
2KD1	HNCA	600	N	128	103.59	130.59	C	128	40.40	72.46	HN	512	4.78	11.76				
2KD1	HNCO	600	N	128	103.59	130.59	C	128	167.32	189.41	HN	512	4.78	11.77				
2KD1	N15HSQC	600	N	616	79.87	140.03	H	512	4.78	11.76								
2KD1	N15NOESY	600	N	128	103.59	130.59	HN	512	4.78	11.77	H	512	-2.22	11.78				
2LTL	C13HSQC_@ALI	800	C	512	6.52	76.52	H	2048	-2.17	11.78								
2LTL	C13HSQC_@ARO	800	C	128	112.66	142.66	H	1024	4.81	11.78								
2LTL	C13NOESY_@ALI	800	C	128	19.50	89.50	HC	564	-0.81	6.87	H	412	-0.82	10.44				
2LTL	C13NOESY_@ARO	800	C	64	112.77	142.77	HC	174	6.00	8.38	H	256	-1.69	11.31				
2LTL	CBCANH	800	N	64	103.14	133.13	C	256	4.13	79.13	HN	512	4.81	11.78				
2LTL	CBCAcoNH	800	N	64	103.14	133.13	C	256	4.13	79.13	HN	512	4.80	11.78				
2LTL	CCHTOCSY_@ALI	800	C1	64	29.39	53.40	C2	128	3.98	78.98	H1	1024	-2.17	11.77				
2LTL	HBHAcoNH	800	N	64	103.13	133.13	HN	512	4.81	11.78	H	256	-0.44	6.56				
2LTL	N15HSQC	800	N	512	102.99	132.99	H	1024	4.80	11.78								
2LTL	N15NOESY	800	N	128	102.51	133.51	HN	324	6.00	10.41	H	412	-0.82	10.44				
2KVO	C13HSQC_@ALI	850	C	512	10.80	76.80	H	581	-1.00	7.51								
2KVO	C13HSQC_@ARO	600	C	445	112.95	139.03	H	229	5.49	9.51								
2KVO	C13NOESY_@ALI	850	C	256	9.92	77.92	HC	429	-1.03	6.48	H	256	-1.47	11.04				
2KVO	C13NOESY_@ARO	850	C	256	111.30	141.30	HC	287	4.97	10.00	H	512	-1.20	10.80				
2KVO	CBCANH	600	N	128	106.03	132.03	C	128	12.07	80.07	HN	286	5.98	11.00				
2KVO	CBCAcoNH	600	N	128	106.03	132.03	C	128	12.07	80.07	HN	286	5.97	10.99				
2KVO	CCHTOCSY_@ALI	600	C1	256	8.15	78.16	C2	256	8.15	78.15	H1	399	-1.03	5.97				
2KVO	CCNOESY_@ALI	600	C1	64	13.29	33.99	C2	32	13.29	33.99	H1	128	-0.53	6.47	H2	1024	-3.70	9.64
2KVO	CcoNH	600	N	128	106.03	132.03	C	256	10.23	78.23	HN	286	5.97	10.99				
2KVO	HBHAcoNH	600	N	256	106.08	132.08	HN	308	6.02	11.03	H	128	-0.43	7.57				
2KVO	HCCHCOSY_@ALI	600	C	256	8.05	78.05	HC	241	-1.02	6.52	H	461	-0.99	6.52				
2KVO	HCCHTOCSY_@ALI	600	C	256	8.05	78.05	HC	128	-1.16	6.84	H	461	-0.99	6.52				
2KVO	HCcoNH_@ALI	600	N	128	106.02	132.02	H	256	-0.70	6.30	HN	286	5.97	10.99				
2KVO	HNCA	600	N	128	106.03	132.03	C	128	40.99	70.99	HN	286	5.98	11.00				
2KVO	HNCO	600	N	128	106.03	132.03	C	64	169.46	182.72	HN	286	5.98	11.00				
2KVO	HNcoCA	600	N	128	106.03	132.03	C	128	40.99	70.99	HN	286	5.98	11.00				
2KVO	N15HSQC	850	N	1024	106.13	132.13	H	342	5.99	11.00								
2KVO	N15NOESY	850	N	256	106.12	132.12	HN	342	5.99	11.00	H	256	-0.70	10.30				
1T0Y	C13HSQC_@ALI	600	C	1024	22.50	63.92	H	2048	-3.54	13.12								
1T0Y	C13HSQC_@ARO	600	C	256	110.43	143.56	H	1024	4.78	13.11								
1T0Y	C13NOESY	600	C	128	22.51	63.92	HC	1024	-3.55	13.11	H	256	-1.16	10.74				
1T0Y	C13NOESY_@ARO	600	C	128	110.80	143.36	HC	97	6.10	7.68	H	227	-0.23	10.32				
1T0Y	CBCANH	600	N	128	102.66	135.54	C	128	1.08	83.91	HN	512	4.79	13.12				
1T0Y	CcoNH	600	N	128	102.66	135.54	C	128	1.73	84.56	HN	512	4.79	13.12				
1T0Y	HCCHTOCSY_@ALI	600	C	128	22.51	63.93	HC	256	-2.13	8.28	H	512	-2.13	8.29				

PDB	Spectrum name	MHz	L1	Size	Range (ppm)		L2	Size	Range (ppm)		L3	Size	Range (ppm)		L4	Size	Range (ppm)	
1T0Y	HNCA	600	N	128	102.66	135.54	C	128	33.40	74.82	HN	512	4.79	13.12				
1T0Y	HNCO	600	N	128	102.66	135.54	C	128	168.41	181.66	HN	512	4.79	13.12				
1T0Y	HNcaCO	600	N	128	102.66	135.55	C	128	168.41	181.66	HN	512	4.79	13.12				
1T0Y	HNcoCA	600	N	128	102.66	135.54	C	128	33.41	74.82	HN	512	4.79	13.12				
1T0Y	N15HSQC	600	N	1024	102.62	135.51	H	1024	4.79	13.12								
1T0Y	N15NOESY	600	N	128	102.63	135.51	HN	512	4.78	13.12	H	256	-1.17	10.73				
1T0Y	N15TOCSY_@ALI	600	N	128	102.55	135.44	HN	512	4.78	13.11	H	256	-1.17	10.73				
2KCD	C13HSQC_@ALI	850	C	1024	5.61	85.58	H	1024	-2.69	12.31								
2KCD	C13HSQC_@ARO	850	C	909	112.91	139.54	H	240	5.55	9.07								
2KCD	C13NOESY	850	C	256	2.11	82.08	HC	1024	-2.70	12.30	H	256	-1.45	11.05				
2KCD	C13NOESY_@ALI	750	C	256	30.96	54.96	HC	1024	-3.23	12.79	H	256	-1.22	10.78				
2KCD	C13NOESY_@ARO	850	C	64	111.30	139.30	HC	240	5.49	9.01	H	256	-1.45	11.05				
2KCD	CBCACOcaHA	600	C	128	5.56	80.56	CO	128	166.85	180.85	HA	309	0.97	7.00				
2KCD	CBCANH	850	N	256	105.49	134.51	C	128	12.49	70.96	HN	376	5.98	11.49				
2KCD	CBCAcoNH	850	N	256	105.49	134.51	C	128	12.90	71.38	HN	376	5.99	11.50				
2KCD	CCHTOCSY_@ALI	600	C1	256	8.00	78.00	C2	256	8.00	78.00	H1	385	-1.02	6.49				
2KCD	CCNOESY_@ALI	600	C1	64	13.35	34.05	C2	32	13.35	34.05	H1	128	-0.50	6.50	H2	1024	-3.67	9.66
2KCD	CcoNH	600	N	256	105.40	134.40	C	128	14.92	74.92	HN	309	4.97	11.00				
2KCD	HBHAcoNH	850	N	128	105.56	134.58	HN	376	5.98	11.49	H	128	0.38	6.26				
2KCD	HCCHCOSY_@ALI	750	C	256	7.95	77.95	HC	241	-1.06	6.47	H	609	-2.04	7.48				
2KCD	HCCHTOCSY_@ALI	850	C	256	2.11	82.08	HC	256	-1.20	6.80	H	1024	-2.70	12.30				
2KCD	HNCA	850	N	256	105.49	134.51	C	256	42.18	68.18	HN	376	5.99	11.50				
2KCD	HNCO	850	N	128	105.49	134.51	C	64	170.18	181.88	HN	376	5.99	11.50				
2KCD	HNcoCA	600	N	128	105.59	134.27	C	128	40.98	70.99	HN	308	4.95	10.98				
2KCD	N15HSQC	850	N	1024	105.49	134.51	H	376	5.99	11.50								
2KCD	N15NOESY	850	N	128	105.49	134.51	HN	376	5.99	11.50	H	256	-1.45	11.05				
2KRT	C13HSQC_@ALI	600	C	512	-2.18	77.40	H	999	-0.52	5.99								
2KRT	C13NOESY_@ALI	800	C	256	19.78	89.78	HC	515	-0.51	6.50	H	512	-2.20	11.80				
2KRT	C13NOESY_@ARO	800	C	256	113.84	141.84	HC	512	4.80	11.78	H	256	-2.20	11.80				
2KRT	CBCANH	600	N	256	101.46	134.37	C	256	8.45	88.46	HN	512	4.77	11.44				
2KRT	CBCAcoNH	600	N	256	99.86	136.06	C	256	12.07	85.02	HN	256	4.77	11.44				
2KRT	CCHTOCSY_@ALI	600	C1	256	25.54	49.54	C2	256	0.98	73.93	H1	269	-0.53	6.48				
2KRT	CcoNH	600	N	256	99.86	136.06	C	256	12.04	84.99	HN	256	4.78	11.45				
2KRT	HBHAcoNH	600	N	512	98.93	135.14	HN	512	4.78	11.45	H	512	-0.24	9.76				
2KRT	HCCHTOCSY_@ALI	600	C	256	33.53	57.53	HC	256	-1.90	11.44	H	269	-0.52	6.49				
2KRT	HCcoNH_@ALI	600	N	512	98.95	135.16	H	512	-0.24	9.76	HN	512	4.78	11.45				
2KRT	HNCA	600	N	256	101.51	134.42	C	256	34.66	74.44	HN	256	4.77	11.77				
2KRT	HNCO	600	N	256	99.86	136.06	C	256	167.92	185.16	HN	256	4.77	11.44				
2KRT	HNHA	500	N	256	99.07	138.56	H	256	-2.22	11.78	HN	512	4.77	11.77				
2KRT	N15HSQC	800	N	256	101.11	135.11	H	512	4.80	11.78								
2KRT	N15NOESY	800	N	256	102.69	133.52	HN	512	4.80	11.77	H	256	-2.20	11.80				
2LFI	C13HSQC_@ALI	850	C	1024	5.83	85.80	H	615	-2.02	6.99								
2LFI	C13HSQC_@ARO	600	C	1024	115.10	145.10	H	286	4.98	10.00								
2LFI	C13NOESY_@ALI	850	C	512	10.84	76.82	HC	401	-1.02	6.00	H	256	-1.20	10.80				
2LFI	C13NOESY_@ARO	850	C	128	111.30	141.30	HC	201	5.48	9.00	H	256	-0.70	10.30				
2LFI	CBCANH	600	N	256	105.51	134.51	C	256	13.12	79.12	HN	258	5.98	10.51				
2LFI	CCHTOCSY_@ALI	600	C1	256	2.26	68.26	C2	256	2.18	68.18	H1	428	-1.03	6.49				
2LFI	HCCHCOSY_@ALI	600	C	512	10.15	76.15	HC	256	-0.20	5.80	H	542	-2.02	7.50				
2LFI	HCCHTOCSY_@ALI	600	C	512	10.15	76.15	HC	256	-0.20	5.80	H	542	-2.01	7.50				
2LFI	HNCA	600	N	256	105.51	134.51	C	128	41.01	71.01	HN	258	5.97	10.50				
2LFI	HNCO	600	N	256	105.51	134.51	C	64	168.05	183.96	HN	258	5.97	10.50				
2LFI	HNcoCA	600	N	256	105.51	134.51	C	128	41.10	71.11	HN	258	5.97	10.50				
2LFI	N15HSQC	850	N	512	105.60	134.60	H	401	4.97	12.00								
2LFI	N15NOESY	850	N	128	105.59	134.59	HN	401	4.97	12.00	H	512	-1.20	10.80				
2JQN	C13HSQC_@ALI	600	C	1024	1.78	81.79	H	2048	-3.19	12.83								
2JQN	C13HSQC_@ARO	800	C	128	107.65	147.66	H	2048	-2.17	11.78								
2JQN	C13NOESY_@ALI	800	C	256	33.74	57.74	HC	552	-1.03	6.49	H	512	-0.93	10.56				
2JQN	C13NOESY_@ARO	800	C	256	112.79	142.80	HC	512	4.81	11.78	H	512	-0.94	10.56				
2JQN	HCCHTOCSY_@ALI	600	C	512	31.75	55.76	HC	256	-3.42	7.58	H	550	-1.03	6.47				
2JQN	HNCA	800	N	256	100.68	135.68	C	512	40.82	72.83	HN	512	4.84	11.82				
2JQN	HNCO	800	N	256	98.63	133.62	C	256	167.75	189.83	HN	512	4.81	11.78				
2JQN	HNcaCO	800	N	256	98.64	133.64	C	256	167.73	189.81	HN	512	4.81	11.78				
2JQN	HNcoCA	800	N	256	100.68	135.68	C	512	40.77	72.78	HN	512	4.84	11.81				
2JQN	N15HSQC	800	N	1024	98.63	133.63	H	1024	4.82	11.79								
2JQN	N15NOESY	800	N	256	102.14	137.14	HN	512	4.85	11.82	H	512	-2.14	11.85				
2L7Q	C13HSQC_@ALI	600	C	1024	2.10	82.10	H	385	-1.03	6.49								
2L7Q	C13NOESY_@ALI	750	C	256	9.00	77.00	HC	481	-1.02	6.50	H	256	-1.41	11.01				
2L7Q	C13NOESY_@ARO	750	C	128	110.00	140.00	HC	224	4.99	8.50	H	256	-1.41	11.01				
2L7Q	CBCANH	600	N	256	101.58	134.57	C	128	11.00	81.00	HN	563	5.48	10.99				
2L7Q	CBCAcoNH	600	N	256	101.58	134.57	C	128	11.00	81.00	HN	563	5.48	10.99				
2L7Q	CCHTOCSY_@ALI	600	C1	128	38.05	62.05	C2	256	8.16	78.16	H1	385	-1.03	6.49				
2L7Q	HBHAcoNH	600	N	256	101.58	134.57	HN	256	5.97	10.98	H	128	-1.22	6.78				
2L7Q	HCCHCOSY_@ALI	600	C	256	8.08	78.08	HC	241	-1.04	6.49	H	487	-2.02	7.50				
2L7Q	HCCHTOCSY_@ALI	600	C	256	7.84	77.83	HC	241	-1.06	6.47	H	486	-2.04	7.49				
2L7Q	HNCA	600	N	128	105.57	134.57	C	128	40.93	70.93	HN	286	5.98	11.00				
2L7Q	HNCO	600	N	256	101.58	134.57	C	128	165.69	182.31	HN	256	5.96	10.98				
2L7Q	HNcoCA	600	N	128	105.57	134.57	C	128	40.93	70.93	HN	286	5.98	11.01				
2L7Q	N15NOESY	800	N	256	101.55	134.46	HN	347	5.49	11.01	H	256	-1.39	11.02				
2KFP	C13HSQC_@ALI	850	C	256	3.80	83.80	H	547	-1.02	6.99								
2KFP	C13HSQC_@ARO	600	C	512	110.00	140.00	H	231	5.48	10.00								
2KFP	C13NOESY_@ALI	850	C	256	9.40	78.20	HC	547	-1.02	7.00	H	256	-1.08	10.68				
2KFP	C13NOESY_@ARO	850	C	128	112.30	140.31	HC	240	5.49	9.01	H	256	-1.08	10.68				
2KFP	CBCANH	600	N	128	102.50	132.50	C	128	13.50	78.50	HN	258	5.98	11.01				
2KFP	CBCAcoNH	600	N	256	102.50	132.50	C	119	14.52	74.94	HN	258	5.98	11.01				
2KFP	CCHTOCSY_@ALI	600	C1	256	3.28	83.28	C2	256	3.19	83.19	H1	385	-1.02	6.49				
2KFP	CCNOESY_@ALI	600	C1	64	13.29	33.99	C2	64	13.29	33.99	H1	1024	-3.68	9.65	H2	128	-0.51	6.48

PDB	Spectrum name	MHz	L1	Size	Range (ppm)		L2	Size	Range (ppm)		L3	Size	Range (ppm)		L4	Size	Range (ppm)	
2KFP	CcoNH	600	N	256	102.50	132.50	C	128	13.50	78.50	HN	258	5.97	11.01				
2KFP	HBHAcoNH	850	N	128	102.53	132.76	HN	308	5.49	10.01	H	128	0.39	6.27				
2KFP	HCCHCOSY_@ALI	600	C	512	8.12	78.12	HC	102	-1.04	5.97	H	385	-1.02	6.50				
2KFP	HCCHTOCSY_@ALI	600	C	512	8.08	78.08	HC	212	-1.04	6.00	H	385	-1.02	6.50				
2KFP	HNCA	600	N	256	102.50	132.50	C	128	40.86	70.86	HN	258	5.96	11.00				
2KFP	HNCO	600	N	256	102.50	132.50	C	128	165.50	182.50	HN	258	5.97	11.01				
2KFP	HNcoCA	600	N	256	102.50	132.50	C	128	41.00	71.00	HN	258	5.98	11.01				
2KFP	N15HSQC	850	N	256	102.56	132.64	H	342	5.99	11.00								
2KFP	N15NOESY	850	N	256	102.56	132.64	HN	308	5.99	10.50	H	256	-1.08	10.68				
1SE9	C13HSQC_@ARO	600	C	256	110.56	143.69	H	1024	4.77	13.10								
1SE9	C13NOESY	600	C	128	22.48	63.89	HC	1024	-3.53	13.14	H	256	-0.98	10.59				
1SE9	C13NOESY_@ARO	600	C	128	110.55	143.69	HC	512	4.79	13.12	H	256	-0.99	10.58				
1SE9	CBCANH	600	N	128	102.68	135.56	C	128	1.13	83.96	HN	512	4.79	13.12				
1SE9	CcoNH	600	N	128	102.69	135.58	C	128	1.78	84.61	HN	512	4.79	13.13				
1SE9	HCCHTOCSY_@ALI	600	C	128	22.49	63.90	HC	256	-2.11	8.30	H	512	-2.11	8.30				
1SE9	HNCA	600	N	128	102.67	135.56	C	128	33.49	74.91	HN	512	4.79	13.12				
1SE9	HNCO	600	N	128	102.68	135.57	C	128	168.40	181.65	HN	512	4.79	13.12				
1SE9	HNcaCO	600	N	128	102.69	135.58	C	128	168.43	181.69	HN	512	4.79	13.12				
1SE9	HNcoCA	600	N	128	102.65	135.53	C	128	33.50	74.92	HN	512	4.79	13.12				
1SE9	N15HSQC	600	N	1024	102.68	135.57	H	1024	4.79	13.12								
1SE9	N15NOESY	600	N	128	102.63	135.51	HN	512	4.79	13.12	H	256	-0.99	10.58				
2L3G	C13HSQC_@ARO	600	C	256	112.54	142.54	H	2048	-1.92	11.42								
2L3G	C13NOESY_@ALI	800	C	112	19.47	80.72	HC	415	-0.27	5.38	H	414	-0.56	10.76				
2L3G	C13NOESY_@ARO	800	C	64	112.74	142.74	HC	99	6.48	7.83	H	443	-0.51	10.74				
2L3G	CBCANH	600	N	128	102.10	133.36	C	128	13.27	83.28	HN	512	4.75	11.42				
2L3G	CBCAcoNH	600	N	128	102.22	133.48	C	128	13.27	83.28	HN	512	4.75	11.42				
2L3G	CCHTOCSY_@ALI	600	C1	128	25.26	49.27	C2	256	0.79	73.74	H1	1024	-1.93	11.42				
2L3G	CcoNH	600	N	128	102.22	133.48	C	128	8.44	88.03	HN	512	4.75	11.42				
2L3G	HBHAcoNH	600	N	128	102.10	133.36	HN	512	4.75	11.84	H	128	-0.25	9.76				
2L3G	HNCO	600	N	64	102.22	133.48	C	64	167.78	185.02	HN	512	4.75	11.42				
2L3G	N15HSQC	600	N	512	102.19	133.45	H	1024	4.75	11.76								
2L3G	N15NOESY	800	N	128	102.58	133.38	HN	385	5.24	10.49	H	414	-0.57	10.75				
2L3B	C13HSQC_@ALI	850	C	1024	0.67	80.63	H	401	-0.52	6.50								
2L3B	C13NOESY_@ALI	850	C	256	8.62	78.65	HC	429	-1.01	6.50	H	256	-1.22	10.78				
2L3B	C13NOESY_@ARO	850	C	128	111.16	141.16	HC	201	5.47	8.99	H	512	-1.23	10.77				
2L3B	CBCANH	600	N	256	102.89	132.89	C	256	16.00	76.00	HN	286	5.97	11.00				
2L3B	CBCAcoNH	600	N	256	102.89	132.89	C	128	16.00	76.00	HN	286	5.97	11.00				
2L3B	CCHTOCSY_@ALI	600	C1	256	8.00	78.00	C2	256	8.00	78.00	H1	428	-1.02	6.50				
2L3B	CCNOESY_@ALI	600	C1	64	13.29	33.99	C2	64	13.29	33.99	H1	1024	-3.70	9.64	H2	128	-0.53	6.47
2L3B	HBHAcoNH	600	N	256	102.89	132.89	HN	286	5.97	11.00	H	128	-0.22	5.78				
2L3B	HCCHCOSY_@ALI	600	C	256	7.92	77.92	HC	256	-0.20	5.80	H	371	-0.51	6.00				
2L3B	HCCHTOCSY_@ALI	600	C	256	7.91	77.92	HC	256	-0.20	5.79	H	399	-1.00	6.00				
2L3B	HNCA	600	N	256	102.93	132.93	C	128	40.93	70.93	HN	286	5.97	11.00				
2L3B	HNCO	600	N	256	102.93	132.93	C	64	168.00	183.91	HN	286	5.97	11.00				
2L3B	HNcoCA	600	N	256	102.93	132.93	C	128	40.93	70.93	HN	286	5.97	11.00				
2L3B	N15HSQC	850	N	1024	103.01	133.01	H	286	5.99	11.00								
2L3B	N15NOESY	850	N	256	103.00	133.00	HN	286	5.99	11.00	H	512	-1.22	10.78				
2LRH	C13HSQC_@ALI	800	C	512	6.57	76.57	H	2048	-2.17	11.78								
2LRH	C13HSQC_@ARO	800	C	128	112.67	142.66	H	248	6.00	7.69								
2LRH	C13NOESY_@ALI	800	C	128	19.53	89.53	HC	524	-0.81	6.33	H	512	-2.20	11.80				
2LRH	C13NOESY_@ARO	800	C	64	112.66	142.66	HC	124	6.00	7.69	H	256	-1.69	11.31				
2LRH	CBCANH	800	N	64	103.57	132.57	C	256	4.10	79.10	HN	512	4.81	11.78				
2LRH	CBCAcoNH	800	N	64	103.59	132.59	C	256	4.14	79.14	HN	512	4.80	11.78				
2LRH	CCHTOCSY_@ALI	800	C1	128	29.56	53.56	C2	128	4.13	79.14	H1	1024	-2.18	11.77				
2LRH	HBHAcoNH	800	N	64	103.56	132.56	HN	512	4.80	11.78	H	256	-0.45	6.55				
2LRH	HNCO	800	N	64	103.56	132.56	C	64	167.58	189.58	HN	512	4.80	11.78				
2LRH	N15HSQC	800	N	512	103.55	132.55	H	1024	4.80	11.78								
2LRH	N15NOESY	800	N	128	102.30	133.48	HN	286	6.04	9.93	H	382	-0.23	10.21				
1VEE	C13HSQC_@ALI	800	C	512	-0.34	74.49	H	1029	-1.04	5.96								
1VEE	C13NOESY_@ALI	800	C	128	8.61	46.72	HC	1029	-1.04	5.96	H	800	-2.31	11.64				
1VEE	C13NOESY_@ARO	800	C	128	109.46	135.00	HC	368	5.46	7.97	H	800	-2.31	11.64				
1VEE	CBCANH	600	N	128	102.32	135.20	C	256	-0.25	74.70	HN	573	6.29	10.20				
1VEE	CBCAcoNH	600	N	128	102.32	135.20	C	256	0.08	75.02	HN	573	6.29	10.20				
1VEE	CcoNH	600	N	128	102.32	135.20	C	256	-0.09	74.85	HN	661	6.29	10.80				
1VEE	HBHAcoNH	600	N	128	102.34	135.22	HN	661	6.29	10.80	H	256	-1.60	10.98				
1VEE	HCCHCOSY_@ARO	600	C	128	109.55	135.07	HC	286	5.49	7.99	H	571	5.49	7.99				
1VEE	HCCHTOCSY_@ALI	600	C	128	0.14	75.08	HC	514	-1.02	5.99	H	1027	-1.01	6.00				
1VEE	HCcoNH_@ALI	600	N	128	102.32	135.20	H	256	-1.60	10.98	HN	661	6.29	10.80				
1VEE	HNCA	600	N	128	102.32	135.20	C	256	37.97	70.03	HN	573	6.29	10.20				
1VEE	HNCO	600	N	128	102.32	135.20	C	256	161.95	184.04	HN	573	6.29	10.20				
1VEE	HNcaCO	600	N	128	102.34	135.22	C	256	161.97	184.05	HN	573	6.29	10.20				
1VEE	HNcoCA	600	N	128	102.32	135.20	C	256	37.97	70.03	HN	573	6.29	10.20				
1VEE	N15HSQC	800	N	512	102.31	135.19	H	574	6.28	10.18								
1VEE	N15NOESY	800	N	128	102.32	135.20	HN	662	6.28	10.78	H	800	-2.29	11.65				
2K1G	C13HSQC_@ALI	800	C	1024	5.35	80.31	H	1024	-2.20	11.75								
2K1G	C13HSQC_@ARO	800	C	1024	112.76	142.76	H	512	4.78	11.76								
2K1G	C13NOESY	800	C	512	15.86	85.86	HC	1024	-2.19	11.76	H	440	-1.04	10.99				
2K1G	C13NOESY_@ARO	800	C	256	112.81	142.81	HC	512	4.75	11.73	H	512	-0.99	10.50				
2K1G	CBCANH	800	N	256	103.11	133.11	C	512	4.26	79.33	HN	512	4.79	11.76				
2K1G	CBCAcoNH	600	N	256	101.55	134.46	C	512	15.46	81.77	HN	512	4.79	11.79				
2K1G	CCHTOCSY_@ALI	600	C1	256	25.57	49.57	C2	512	4.38	70.69	H1	576	-1.01	6.49				
2K1G	CCHTOCSY_@ARO	800	C1	256	112.71	142.72	C2	256	112.85	142.86	H1	1024	-2.20	11.75				
2K1G	CcoNH	600	N	256	101.51	134.42	C	512	15.42	81.73	HN	512	4.79	11.78				
2K1G	HBHAcoNH	800	N	256	103.11	133.11	HN	512	4.79	11.76	H	512	1.03	6.04				
2K1G	HCCHCOSY_@ALI	800	C	256	29.72	53.72	HC	512	0.28	6.28	H	552	-1.02	6.50				
2K1G	HCCHTOCSY_@ALI	600	C	256	25.51	49.52	HC	512	-0.20	9.80	H	550	-1.01	6.51				

PDB	Spectrum name	MHz	L1	Size	Range (ppm)		L2	Size	Range (ppm)		L3	Size	Range (ppm)		L4	Size	Range (ppm)	
2K1G	HNCA	600	N	256	101.55	134.46	C	512	43.59	73.59	HN	512	4.80	11.80				
2K1G	HNCO	800	N	256	103.14	133.15	C	512	168.75	188.75	HN	512	4.79	11.77				
2K1G	HNcaCO	800	N	256	103.11	133.11	C	512	168.75	188.75	HN	512	4.79	11.76				
2K1G	N15HSQC	800	N	1024	103.11	133.11	H	2048	-2.19	11.76								
2K1G	N15NOESY	800	N	256	103.15	133.14	HN	512	4.79	11.76	H	512	-0.96	10.54				
2KKZ	C13HSQC_@ALI	600	C	1024	4.25	79.25	H	2048	-2.19	11.79								
2KKZ	C13HSQC_@ARO	800	C	1024	112.78	142.78	H	1024	4.82	11.79								
2KKZ	C13NOESY_@ALI	800	C	512	19.78	89.78	HC	551	-1.01	6.49	H	440	-1.03	11.00				
2KKZ	C13NOESY_@ARO	800	C	512	112.78	142.78	HC	512	4.81	11.78	H	512	-0.69	10.31				
2KKZ	CBCANH	800	N	256	104.14	132.14	C	512	4.10	79.10	HN	512	4.81	11.78				
2KKZ	CBCAcoNH	800	N	256	101.14	135.14	C	512	4.32	79.32	HN	512	4.81	11.78				
2KKZ	CCHTOCSY_@ALI	600	C1	256	31.75	55.75	C2	256	8.84	78.84	H1	1024	-2.19	11.79				
2KKZ	HCCHCOSY_@ALI	800	C	256	29.80	53.80	HC	512	-0.81	6.68	H	1024	-2.16	11.78				
2KKZ	HCCHTOCSY_@ALI	600	C	256	31.75	55.75	HC	512	-0.07	6.43	H	1024	-2.19	11.79				
2KKZ	HNCA	800	N	256	104.17	132.17	C	512	40.74	72.81	HN	512	4.81	11.78				
2KKZ	HNCO	800	N	256	104.14	132.14	C	512	167.79	189.79	HN	512	4.81	11.78				
2KKZ	HNcaCO	800	N	256	104.14	132.14	C	512	167.76	189.76	HN	512	4.81	11.78				
2KKZ	HNcoCA	800	N	256	104.14	132.14	C	512	40.77	72.78	HN	512	4.87	11.84				
2KKZ	N15HSQC	600	N	1024	103.14	133.15	H	2048	-2.18	11.80								
2KKZ	N15NOESY	800	N	256	104.22	135.05	HN	512	4.82	11.79	H	440	-1.03	11.00				
1VDY	C13HSQC	800	C	512	3.23	46.46	H	1322	-1.00	8.00								
1VDY	C13NOESY	800	C	128	3.23	46.46	HC	1248	-0.51	8.00	H	800	-0.80	10.20				
1VDY	CBCANH	600	N	128	106.27	128.44	C	256	-0.39	74.59	HN	572	6.29	10.19				
1VDY	CBCAcoNH	600	N	128	106.27	128.44	C	256	-0.06	74.91	HN	573	6.29	10.20				
1VDY	CCHTOCSY_@ALI	600	C1	128	3.27	46.49	C2	256	-21.16	70.84	H1	1026	-1.00	6.00				
1VDY	CcoNH	600	N	128	106.27	128.44	C	256	-0.15	74.83	HN	573	6.28	10.19				
1VDY	HBHAcoNH	600	N	128	106.27	128.44	HN	572	6.29	10.19	H	256	-1.00	10.40				
1VDY	HCCHCOSY_@ALI	600	C	128	3.23	46.46	HC	629	-1.00	6.00	H	1105	-1.00	6.00				
1VDY	HCCHTOCSY_@ALI	600	C	128	3.23	46.46	HC	629	-0.99	6.01	H	1105	-1.00	6.01				
1VDY	HCCHTOCSY_@ARO	600	C	128	111.27	137.83	HC	210	6.30	8.01	H	405	6.31	8.01				
1VDY	HCcoNH_@ALI	600	N	128	106.27	128.44	H	256	-1.00	10.39	HN	573	6.28	10.19				
1VDY	HNCA	600	N	128	106.27	128.44	C	256	35.64	67.72	HN	572	6.29	10.20				
1VDY	HNCO	600	N	128	106.28	128.46	C	256	161.51	183.60	HN	573	6.28	10.20				
1VDY	HNcaCO	600	N	128	106.25	128.42	C	256	161.51	183.60	HN	572	6.29	10.19				
1VDY	HNcoCA	600	N	128	106.25	128.42	C	256	35.65	67.73	HN	572	6.29	10.19				
1VDY	N15HSQC	800	N	512	106.27	128.44	H	574	6.29	10.20								
1VDY	N15NOESY	800	N	128	106.27	128.44	HN	574	6.29	10.20	H	800	-1.60	11.00				
2KKL	C13HSQC_@ALI	600	C	481	-0.16	75.00	H	513	-2.01	7.00								
2KKL	C13HSQC_@ARO	850	C	512	111.33	141.34	H	478	4.99	11.99								
2KKL	C13NOESY_@ALI	850	C	256	8.80	78.80	HC	486	-1.01	7.50	H	512	-2.20	11.80				
2KKL	C13NOESY_@ARO	850	C	128	115.30	141.30	HC	124	6.41	8.58	H	227	-1.00	11.42				
2KKL	CBCANH	600	N	128	103.93	133.93	C	128	11.00	81.00	HN	343	5.98	12.01				
2KKL	CBCAcoNH	600	N	128	103.93	133.93	C	128	11.00	81.00	HN	400	4.98	12.01				
2KKL	CCHTOCSY_@ALI	600	C1	256	8.01	78.02	C2	256	7.93	77.93	H1	428	-1.02	6.50				
2KKL	CCNOESY_@ALI	600	C1	32	13.29	33.99	C2	32	13.29	33.99	H1	1024	-3.68	9.66	H2	128	-0.50	6.50
2KKL	CcoNH	600	N	256	103.93	133.93	C	128	11.00	81.00	HN	314	5.49	11.00				
2KKL	HBHAcoNH	600	N	128	103.93	133.93	HN	371	5.49	12.01	H	256	-0.19	5.81				
2KKL	HCCHCOSY_@ALI	600	C	256	8.08	78.08	HC	128	-0.72	6.28	H	428	-1.01	6.51				
2KKL	HCCHTOCSY_@ALI	600	C	256	8.08	78.09	HC	256	-0.70	6.30	H	542	-2.01	7.51				
2KKL	HCcoNH_@ALI	600	N	256	103.93	133.93	H	128	-0.94	6.56	HN	371	5.49	12.00				
2KKL	HNCA	600	N	128	103.93	133.93	C	64	41.00	71.00	HN	400	4.98	12.01				
2KKL	HNCO	600	N	128	103.93	133.93	C	64	165.50	182.50	HN	400	4.98	12.00				
2KKL	HNcoCA	600	N	128	103.93	133.93	C	64	41.00	71.00	HN	400	4.98	12.01				
2KKL	N15HSQC	850	N	256	104.03	134.03	H	401	4.97	12.00								
2KKL	N15NOESY	850	N	128	104.07	134.07	HN	444	5.49	11.99	H	256	-1.45	11.05				
2N4B	C13HSQC_@ALI	850	C	256	5.71	85.71	H	547	-1.71	6.30								
2N4B	C13HSQC_@ARO	600	C	1024	108.00	142.00	H	228	4.99	8.99								
2N4B	C13NOESY_@ALI	850	C	256	11.72	75.72	HC	444	-0.51	6.00	H	256	-1.02	10.58				
2N4B	C13NOESY_@ARO	850	C	128	111.30	141.30	HC	206	5.48	8.50	H	256	-1.07	10.63				
2N4B	CBCANH	600	N	128	105.30	132.30	C	256	14.00	78.00	HN	428	2.98	10.50				
2N4B	CBCAcoNH	600	N	128	105.30	132.30	C	128	14.00	78.00	HN	428	2.98	10.50				
2N4B	CCHTOCSY_@ALI	600	C1	256	10.07	76.07	C2	256	10.07	76.07	H1	853	-1.02	6.49				
2N4B	CCNOESY_@ALI	600	C1	64	13.29	33.99	C2	64	13.29	33.99	H1	1024	-3.70	9.64	H2	128	-0.53	6.47
2N4B	CcoNH	600	N	256	105.30	132.30	C	256	13.00	79.00	HN	428	2.98	10.50				
2N4B	HBHAcoNH	600	N	256	105.30	132.30	HN	428	2.98	10.50	H	256	0.37	6.37				
2N4B	HCCHTOCSY_@ALI	600	C	256	10.00	78.00	HC	256	-0.71	6.29	H	542	-2.01	7.51				
2N4B	HNCO	600	N	128	105.30	132.30	C	64	166.04	181.96	HN	428	2.98	10.50				
2N4B	HNcoCA	600	N	128	105.30	132.30	C	128	40.93	70.93	HN	428	2.98	10.50				
2N4B	N15HSQC	850	N	1024	105.40	132.40	H	512	2.98	10.49								
2N4B	N15NOESY	850	N	128	105.43	132.43	HN	481	2.98	10.49	H	256	-1.22	10.78				
2L8V	C13HSQC_@ALI	850	C	512	10.04	76.03	H	401	-1.02	6.01								
2L8V	C13NOESY_@ALI	850	C	256	10.43	76.41	HC	429	-1.01	6.50	H	256	-1.22	10.78				
2L8V	C13NOESY_@ARO	850	C	128	111.97	141.98	HC	151	6.18	8.82	H	454	-0.10	10.55				
2L8V	CBCANH	600	N	128	104.49	131.49	C	128	15.02	77.02	HN	286	5.48	10.50				
2L8V	CBCAcoNH	600	N	128	104.49	131.49	C	128	15.02	77.02	HN	286	5.48	10.50				
2L8V	CCHTOCSY_@ALI	600	C1	256	8.16	78.17	C2	256	8.16	78.16	H1	839	-1.02	6.49				
2L8V	CCNOESY_@ALI	600	C1	64	13.29	33.99	C2	32	13.29	33.99	H1	128	-0.54	6.46	H2	1024	-3.73	9.65
2L8V	HBHAcoNH	600	N	128	104.49	131.49	HN	314	5.47	10.98	H	256	0.54	6.54				
2L8V	HCCHCOSY_@ALI	600	C	256	8.08	78.08	HC	256	-0.20	5.79	H	428	-1.02	6.50				
2L8V	HCCHTOCSY_@ALI	600	C	256	8.10	78.11	HC	256	-0.20	5.79	H	542	-2.01	7.51				
2L8V	HNCA	600	N	128	104.49	131.49	C	128	41.01	71.01	HN	286	5.48	10.50				
2L8V	HNCO	600	N	128	104.49	131.49	C	64	168.00	183.91	HN	280	5.58	10.49				
2L8V	HNcoCA	600	N	128	104.49	131.49	C	128	41.01	71.01	HN	286	5.48	10.50				
2L8V	N15HSQC	850	N	1024	104.59	131.59	H	315	5.47	10.99								
2L8V	N15NOESY	850	N	256	104.59	131.59	HN	377	5.47	11.00	H	256	-0.70	11.30				
2LGH	C13HSQC_@ALI	850	C	256	2.58	82.58	H	581	-1.01	7.50								

PDB	Spectrum name	MHz	L1	Size	Range (ppm)	L2	Size	Range (ppm)	L3	Size	Range (ppm)	L4	Size	Range (ppm)
2LGH	C13HSQC_@ARO	850	C	256	109.96 139.97	H	386	4.98 11.01						
2LGH	C13NOESY_@ALI	850	C	512	11.69 75.69	HC	512	-1.01 6.49	H	256	-1.47 11.03			
2LGH	C13NOESY_@ARO	850	C	256	111.26 141.27	HC	342	4.99 10.00	H	256	-1.47 11.03			
2LGH	CBCANH	600	N	128	104.64 132.24	C	128	14.00 78.00	HN	342	5.00 11.00			
2LGH	CBCAcoNH	600	N	128	104.64 132.24	C	128	14.00 78.00	HN	342	5.00 11.00			
2LGH	CCHTOCSY_@ALI	600	C1	256	4.43 70.43	C2	256	4.43 70.43	H1	399	-1.03 5.98			
2LGH	CCNOESY_@ALI	600	C1	64	13.29 33.99	C2	64	13.29 33.99	H1	1024	-3.70 9.64	H2	128	-0.53 6.47
2LGH	CcoNH	600	N	128	104.64 132.24	C	256	12.97 78.97	HN	342	5.00 11.00			
2LGH	HBHAcoNH	600	N	128	104.64 132.24	HN	342	5.00 11.00	H	128	0.51 6.51			
2LGH	HCCHCOSY_@ALI	600	C	256	10.00 76.00	HC	256	-0.71 6.29	H	428	-1.01 6.51			
2LGH	HCCHTOCSY_@ALI	600	C	256	10.00 78.00	HC	256	-0.70 6.30	H	428	-1.01 6.51			
2LGH	HNCA	600	N	128	104.64 132.24	C	128	40.93 70.93	HN	342	5.00 11.00			
2LGH	HNCO	600	N	128	104.64 132.24	C	128	168.00 183.91	HN	342	5.00 11.00			
2LGH	HNcoCA	600	N	128	104.64 132.24	C	128	40.93 70.93	HN	342	5.00 11.00			
2LGH	N15HSQC	600	N	256	102.97 132.96	H	385	4.99 11.01						
2LGH	N15NOESY	850	N	256	104.73 132.33	HN	343	4.99 11.00	H	512	-1.47 11.03			
2K1S	C13HSQC_@ALI	600	C	512	0.05 80.05	H	400	-0.54 6.49						
2K1S	C13HSQC_@ARO	600	C	512	110.00 140.00	H	286	4.98 10.00						
2K1S	C13NOESY_@ALI	600	C	256	30.97 54.97	HC	439	-1.51 6.21	H	256	-0.70 10.30			
2K1S	C13NOESY_@ARO	600	C	256	115.00 135.00	HC	225	4.98 9.01	H	256	-1.70 11.30			
2K1S	CBCAcoNH	600	N	256	97.00 139.00	C	128	11.00 81.00	HN	286	4.98 10.01			
2K1S	CCNOESY_@ALI	600	C1	64	13.29 33.99	C2	32	13.29 33.99	H1	128	-0.50 6.49	H2	1024	-3.67 9.66
2K1S	CcoNH	600	N	256	97.00 139.00	C	128	15.06 75.06	HN	343	4.99 11.02			
2K1S	HBHAcoNH	600	N	256	97.00 139.00	HN	343	4.98 11.01	H	256	0.30 6.30			
2K1S	HCCHCOSY_@ALI	600	C	256	31.03 55.03	HC	128	-0.72 6.28	H	428	-1.02 6.50			
2K1S	HCCHTOCSY_@ALI	600	C	256	31.03 55.03	HC	128	-0.72 6.28	H	428	-1.02 6.50			
2K1S	HCcoNH_@ALI	600	N	128	97.00 139.00	H	128	-0.72 6.28	HN	400	4.98 12.01			
2K1S	HNCA	600	N	128	97.00 139.00	C	64	40.86 70.86	HN	286	4.98 10.01			
2K1S	HNCO	600	N	256	97.00 139.00	C	256	165.52 182.52	HN	343	4.98 11.01			
2K1S	HNcoCA	600	N	128	97.00 139.00	C	64	41.00 71.00	HN	286	4.98 10.00			
2K1S	N15HSQC	600	N	256	97.00 139.00	H	496	4.99 10.80						
2K1S	N15NOESY	600	N	256	97.00 139.00	HN	285	5.48 10.49	H	256	-0.70 10.30			
2M4F	C13HSQC_@ALI	800	C	1024	6.03 78.12	H	2048	-1.18 10.70						
2M4F	C13HSQC_@ARO	800	C	1024	106.33 156.04	H	1024	4.77 10.71						
2M4F	C13NOESY_@ALI	800	C	256	3.10 83.10	HC	2048	-1.50 11.03	H	1024	-1.49 11.02			
2M4F	CBCANH	600	N	256	96.50 136.00	C	256	12.86 92.86	HN	1024	4.77 11.44			
2M4F	CBCAcoNH	600	N	256	95.71 136.86	C	256	8.10 78.10	HN	1024	4.76 11.43			
2M4F	CcoNH	600	N	256	96.48 135.99	C	512	3.16 83.16	HN	1024	4.76 11.44			
2M4F	HBHAcoNH	600	N	256	96.47 135.97	HN	1024	4.76 11.43	H	256	-2.74 12.27			
2M4F	HCCHCOSY_@ALI	800	C	256	-4.87 75.13	HC	512	-1.49 11.02	H	2048	-1.49 11.02			
2M4F	HCCHTOCSY_@ALI	800	C	256	-4.90 75.10	HC	368	-1.50 7.49	H	1472	-1.49 7.50			
2M4F	HNCA	600	N	256	95.47 136.62	C	256	40.10 70.10	HN	1024	4.76 11.43			
2M4F	HNCO	600	N	128	96.50 136.00	C	256	161.59 186.59	HN	1024	4.76 11.43			
2M4F	HNcaCO	600	N	256	96.48 135.99	C	256	161.59 186.60	HN	1024	4.76 11.44			
2M4F	HNcoCA	600	N	256	96.47 135.97	C	128	41.13 71.13	HN	1024	4.76 11.43			
2M4F	N15HSQC	600	N	1024	97.62 138.77	H	1024	4.75 12.26						
2M4F	N15NOESY	800	N	256	96.30 135.78	HN	1024	4.76 11.02	H	2048	-2.75 12.28			
2M4F	N15TOCSY	600	N	256	96.53 136.03	HN	1024	4.76 11.44	H	256	-2.75 12.27			
2JXP	C13HSQC_@ALI	800	C	512	8.21 83.17	H	479	-0.51 6.01						
2JXP	C13HSQC_@ARO	800	C	256	112.68 142.69	H	512	4.74 11.71						
2JXP	C13NOESY_@ALI	800	C	256	19.68 89.67	HC	474	-0.27 6.19	H	385	-0.53 9.99			
2JXP	CBCANH	800	N	128	106.04 132.04	C	128	4.13 79.20	HN	512	4.71 11.69			
2JXP	CBCAcoNH	600	N	128	106.05 132.05	C	256	4.22 79.19	HN	512	4.71 11.70			
2JXP	CCHTOCSY_@ALI	800	C1	128	29.67 53.67	C2	256	4.16 79.11	H1	479	-0.52 6.00			
2JXP	CCHTOCSY_@ARO	800	C1	128	112.67 142.68	C2	128	112.72 142.72	H1	224	5.75 7.94			
2JXP	HBHAcoNH	800	N	256	106.03 132.04	HN	512	4.73 11.70	H	128	0.97 5.97			
2JXP	HCCHCOSY_@ALI	800	C	128	33.70 57.70	HC	256	-0.22 5.78	H	478	-0.51 6.00			
2JXP	HCCHTOCSY_@ALI	800	C	128	29.67 53.66	HC	128	-0.24 5.75	H	478	-0.51 6.00			
2JXP	HCCHTOCSY_@ARO	800	C	128	112.71 142.72	HC	75	6.04 8.97	H	224	5.74 7.93			
2JXP	HNCA	800	N	128	106.04 132.05	C	256	41.66 71.66	HN	512	4.72 11.70			
2JXP	HNCO	800	N	128	106.05 132.06	C	128	168.69 188.69	HN	512	4.72 11.69			
2JXP	HNcaCO	800	N	128	106.05 132.05	C	128	168.69 188.69	HN	512	4.72 11.70			
2JXP	HNcoCA	800	N	128	106.05 132.05	C	128	41.64 71.64	HN	324	5.92 10.34			
2JXP	N15HSQC	800	N	256	106.05 132.05	H	512	4.72 11.70						
2JXP	N15NOESY	800	N	128	104.03 135.03	HN	512	4.71 11.68	H	385	-0.54 9.99			
2L06	C13HSQC_@ALI	850	C	512	3.75 83.75	H	578	-2.02 7.00						
2L06	C13NOESY_@ALI	850	C	256	9.70 74.70	HC	450	-1.02 6.00	H	256	-0.60 10.20			
2L06	C13NOESY_@ARO	850	C	64	115.91 138.91	HC	240	5.49 9.01	H	256	-0.69 10.31			
2L06	CBCANH	500	N	128	102.19 131.81	C	128	8.50 83.50	HN	285	5.49 10.50			
2L06	CBCAcoNH	500	N	128	102.19 131.81	C	128	8.50 83.50	HN	285	5.48 10.49			
2L06	CCHTOCSY_@ALI	600	C1	256	8.20 78.20	C2	256	8.18 78.18	H1	428	-1.02 6.50			
2L06	CcoNH	500	N	128	102.19 131.81	C	128	8.50 83.50	HN	399	4.96 11.98			
2L06	HBHAcoNH	500	N	128	102.19 131.81	HN	285	5.48 10.49	H	256	-1.23 6.77			
2L06	HCCHCOSY_@ALI	600	C	256	8.23 78.23	HC	243	-0.20 5.49	H	371	-1.02 5.49			
2L06	HCCHTOCSY_@ALI	600	C	256	8.18 78.19	HC	256	-0.20 5.80	H	542	-2.02 7.50			
2L06	HCcoNH_@ALI	500	N	256	102.19 131.81	H	256	-1.24 6.76	HN	285	5.47 10.48			
2L06	HNCO	500	N	128	101.21 132.79	C	64	166.05 181.96	HN	367	5.48 10.50			
2L06	HNcoCA	500	N	128	102.19 131.81	C	128	41.00 71.00	HN	285	5.47 10.49			
2L06	N15HSQC	500	N	256	87.39 146.61	H	367	5.48 10.49						
2L06	N15NOESY	850	N	128	102.30 131.90	HN	321	5.50 10.51	H	256	-0.69 10.31			
2LAH	C13NOESY_@ALI	800	C	128	19.72 88.52	HC	491	-0.78 5.91	H	470	-1.05 11.80			
2LAH	C13NOESY_@ARO	800	C	64	112.16 142.89	HC	173	5.83 8.18	H	486	-1.03 11.30			
2LAH	CBCANH	600	N	128	104.63 130.96	C	128	13.21 83.22	HN	512	4.78 10.78			
2LAH	CBCAcoNH	600	N	128	104.63 130.96	C	128	13.27 83.28	HN	512	4.78 10.78			
2LAH	CCHTOCSY_@ALI	800	C1	128	29.51 53.51	C2	256	4.00 79.00	H1	1024	-2.18 11.77			
2LAH	CcoNH	600	N	128	104.63 130.96	C	128	8.65 88.23	HN	512	4.78 11.87			
2LAH	HBHAcoNH	600	N	128	104.63 130.96	HN	512	4.78 11.87	H	128	-0.24 9.76			

PDB	Spectrum name	MHz	L1	Size	Range (ppm)		L2	Size	Range (ppm)		L3	Size	Range (ppm)		L4	Size	Range (ppm)	
2LAH	HCCHTOCSY_@ARO	800	C	64	111.60	143.60	HC	128	3.04	10.04	H	1024	-2.17	11.77				
2LAH	N15HSQC	800	N	512	103.01	132.01	H	1024	4.80	11.77								
2LAH	N15NOESY	800	N	128	102.69	133.24	HN	496	4.24	10.99	H	451	-1.06	11.28				
2LAK	C13HSQC_@ALI	850	C	256	5.82	85.78	H	492	-1.01	6.20								
2LAK	C13NOESY_@ALI	850	C	256	11.80	75.80	HC	478	-1.01	6.00	H	256	-1.47	11.03				
2LAK	C13NOESY_@ARO	850	C	256	111.30	141.30	HC	342	4.99	10.00	H	256	-1.47	11.03				
2LAK	CBCANH	600	N	256	102.93	132.93	C	128	11.16	81.16	HN	258	5.98	11.02				
2LAK	CBCAcoNH	600	N	256	102.93	132.93	C	128	11.16	81.16	HN	258	5.98	11.01				
2LAK	CCHTOCSY_@ALI	600	C1	256	4.58	70.58	C2	256	4.58	70.58	H1	399	-1.02	5.99				
2LAK	CCNOESY_@ALI	600	C1	64	13.29	33.99	C2	64	13.29	33.99	H1	1024	-3.70	9.64	H2	128	-0.53	6.47
2LAK	CcoNH	600	N	128	102.93	132.93	C	128	6.19	86.19	HN	513	6.00	11.00				
2LAK	HBHAcoNH	600	N	128	102.93	132.93	HN	343	5.98	11.00	H	128	0.51	6.51				
2LAK	HCCHCOSY_@ALI	600	C	256	8.07	78.07	HC	256	-0.71	6.29	H	513	-1.01	6.50				
2LAK	HCCHTOCSY_@ALI	600	C	256	8.00	78.00	HC	256	-0.70	6.30	H	513	-1.01	6.50				
2LAK	HCcoNH_@ALI	600	N	128	102.93	132.93	H	256	-1.21	6.79	HN	343	5.99	11.01				
2LAK	HNCA	600	N	256	102.93	132.93	C	128	40.93	70.93	HN	258	5.98	11.01				
2LAK	HNCO	600	N	256	102.93	132.93	C	128	166.08	185.96	HN	258	5.98	11.02				
2LAK	HNcoCA	600	N	256	102.93	132.93	C	128	41.00	71.00	HN	258	5.98	11.01				
2LAK	N15HSQC	850	N	512	102.96	133.04	H	386	4.98	11.01								
2LAK	N15NOESY	850	N	128	103.00	133.00	HN	386	4.99	11.01	H	256	-1.47	11.03				
2L82	C13NOESY_@ALI	800	C	128	19.41	89.41	HC	584	-2.27	5.69	H	472	-1.75	11.15				
2L82	C13NOESY_@ARO	800	C	44	112.68	133.31	HC	224	5.10	8.15	H	512	-1.78	11.22				
2L82	CBCANH	600	N	128	103.34	132.96	C	128	13.15	83.16	HN	512	4.69	11.36				
2L82	CBCAcoNH	600	N	128	103.34	132.96	C	128	13.15	83.16	HN	512	4.69	11.36				
2L82	CCHTOCSY_@ALI	600	C1	128	25.15	49.15	C2	256	0.67	73.62	H1	1024	-2.41	11.76				
2L82	CcoNH	600	N	128	103.34	132.96	C	128	8.33	87.91	HN	512	4.69	11.78				
2L82	HBHAcoNH	600	N	128	103.34	132.96	HN	512	4.69	11.78	H	128	-0.32	9.69				
2L82	N15HSQC	600	N	256	101.69	134.60	H	1024	4.69	12.03								
2L82	N15NOESY	800	N	128	102.12	133.62	HN	324	6.17	10.59	H	512	-2.30	11.70				
2M47	C13HSQC_@ALI	850	C	256	5.80	85.80	H	547	-1.71	6.30								
2M47	C13HSQC_@ARO	850	C	256	108.03	142.03	H	274	5.48	9.50								
2M47	C13NOESY_@ALI	850	C	256	9.81	77.81	HC	547	-1.71	6.30	H	256	-1.72	11.28				
2M47	C13NOESY_@ARO	850	C	205	111.42	135.44	HC	111	6.20	8.14	H	256	-1.22	10.79				
2M47	CBCANH	600	N	256	102.70	131.70	C	128	14.10	78.10	HN	342	4.99	10.99				
2M47	CBCAcoNH	600	N	128	102.71	131.71	C	128	14.10	78.10	HN	286	5.98	11.00				
2M47	CCHTOCSY_@ALI	600	C1	256	11.19	77.19	C2	256	11.16	77.16	H1	910	-1.72	6.29				
2M47	CcoNH	600	N	256	102.67	131.68	C	256	13.09	79.09	HN	286	5.98	11.00				
2M47	HBHAcoNH	600	N	256	102.67	131.67	HN	286	5.98	11.00	H	256	0.29	6.29				
2M47	HCCHCOSY_@ALI	600	C	256	10.20	76.20	HC	256	-0.73	6.27	H	428	-1.03	6.49				
2M47	HCCHTOCSY_@ALI	600	C	256	10.10	78.10	HC	256	-0.36	5.94	H	542	-2.01	7.51				
2M47	HCcoNH_@ALI	600	N	128	102.71	131.71	H	128	-0.75	6.25	HN	286	5.97	10.99				
2M47	HNCA	850	N	128	105.47	134.49	C	128	39.92	69.92	HN	377	5.48	11.01				
2M47	HNCO	600	N	128	102.71	131.71	C	64	166.05	181.96	HN	286	5.98	11.00				
2M47	HNcoCA	600	N	128	102.71	131.71	C	128	41.06	71.06	HN	286	5.97	11.00				
2M47	N15HSQC	850	N	1024	102.79	131.79	H	343	5.98	11.01								
2M47	N15NOESY	850	N	128	102.80	131.80	HN	386	4.98	11.01	H	256	-1.72	11.28				
2K3A	C13HSQC_@ALI	800	C	512	4.38	79.33	H	478	-0.52	5.99								
2K3A	C13HSQC_@ARO	800	C	512	107.72	147.73	H	512	4.75	11.72								
2K3A	C13NOESY_@ALI	800	C	128	33.78	57.78	HC	478	-0.52	5.99	H	256	-1.01	10.48				
2K3A	C13NOESY_@ARO	800	C	128	112.73	142.72	HC	512	4.74	11.71	H	256	-1.00	10.50				
2K3A	CBCANH	800	N	128	105.58	130.58	C	256	4.18	79.26	HN	512	4.76	11.73				
2K3A	CBCAcoNH	800	N	128	105.64	130.64	C	256	4.10	79.17	HN	512	4.76	11.73				
2K3A	CCHTOCSY_@ALI	800	C1	128	31.73	55.72	C2	256	6.10	81.17	H1	478	-0.52	5.99				
2K3A	HBHAcoNH	800	N	128	105.58	130.58	HN	512	4.76	11.73	H	128	-0.49	6.51				
2K3A	HCCHCOSY_@ALI	800	C	128	31.73	55.72	HC	128	-0.49	6.51	H	478	-0.52	5.99				
2K3A	HCCHTOCSY_@ALI	800	C	128	31.67	55.66	HC	128	-0.50	6.50	H	478	-0.52	5.99				
2K3A	HNCA	800	N	128	105.64	130.64	C	128	40.72	72.72	HN	512	4.75	11.73				
2K3A	HNCO	800	N	128	105.58	130.58	C	128	167.68	189.76	HN	512	4.76	11.73				
2K3A	HNcaCO	800	N	128	105.58	130.58	C	128	167.68	189.76	HN	512	4.76	11.73				
2K3A	HNcoCA	800	N	128	105.58	130.58	C	128	40.72	72.72	HN	512	4.75	11.73				
2K3A	N15HSQC	800	N	256	105.52	130.52	H	512	4.75	11.73								
2K3A	N15NOESY	800	N	128	105.52	130.52	HN	512	4.76	11.73	H	256	-0.99	10.51				
2K3A	N15TOCSY	600	N	64	105.66	130.66	HN	512	4.76	11.75	H	256	-0.73	10.27				
2M7U	C13HSQC_@ALI	800	C	512	1.61	81.61	H	1364	-1.00	9.00								
2M7U	C13NOESY_@ALI	800	C	128	9.76	79.37	HC	875	-1.00	6.50	H	197	-0.56	10.99				
2M7U	C13NOESY_@ARO	800	C	128	110.95	140.78	HC	468	4.99	9.00	H	512	-1.23	13.77				
2M7U	CBCANH	600	N	128	101.27	135.27	C	256	12.38	82.38	HN	1797	5.79	13.11				
2M7U	CBCAcoNH	600	N	128	101.27	135.27	C	256	12.38	82.38	HN	1797	5.80	13.11				
2M7U	CcoNH	600	N	64	101.27	135.27	C	256	7.38	87.38	HN	861	5.79	12.80				
2M7U	HBHAcoNH	600	N	64	101.27	135.27	HN	861	5.79	12.80	H	256	0.27	6.27				
2M7U	HCcoNH_@ALI	600	N	128	101.27	135.27	H	512	-0.73	6.28	HN	1721	5.80	12.80				
2M7U	HNCO	600	N	128	101.27	135.27	C	128	167.87	182.87	HN	860	5.99	13.00				
2M7U	N15HSQC	800	N	512	98.40	138.40	H	1657	5.80	13.90								
2M7U	N15NOESY	800	N	128	101.93	135.93	HN	1533	6.00	12.80	H	778	-1.01	12.80				
2B3W	C13HSQC_@ALI	750	C	256	-2.50	82.50	H	385	-1.02	6.00								
2B3W	C13HSQC_@ARO	750	C	439	110.00	140.00	H	275	4.98	10.00								
2B3W	C13NOESY_@ALI	600	C	256	30.99	54.99	HC	483	-2.02	7.01	H	256	-1.89	11.45				
2B3W	C13NOESY_@ALI_2	800	C	128	31.08	55.08	HC	486	-2.01	7.00	H	256	-1.97	11.53				
2B3W	C13NOESY_@ARO	750	C	128	109.98	139.98	HC	275	4.98	10.00	H	256	-1.89	11.45				
2B3W	CBCANH	600	N	128	100.10	134.66	C	128	15.03	77.03	HN	375	4.99	12.00				
2B3W	CBCAcoNH	600	N	128	100.11	134.67	C	128	14.96	76.96	HN	375	4.99	12.00				
2B3W	CBHDaro	600	C	256	20.00	50.00	H	342	4.99	10.00								
2B3W	CCHTOCSY_@ALI	600	C1	128	30.97	54.98	C2	256	5.52	80.52	H1	1024	-4.80	14.34				
2B3W	CCNOESY_@ALI	600	C1	32	13.25	33.95	C2	32	13.25	33.95	H1	1024	-3.66	9.66	H2	128	-0.50	6.50
2B3W	CcoNH	600	N	128	100.11	134.67	C	128	11.59	73.59	HN	375	4.99	12.00				
2B3W	HCCHCOSY_@ALI	600	C	128	31.02	55.02	HC	241	-1.02	6.51	H	1024	-4.78	14.36				

PDB	Spectrum name	MHz	L1	Size	Range (ppm)		L2	Size	Range (ppm)		L3	Size	Range (ppm)		L4	Size	Range (ppm)	
2B3W	HCCHTOCSY_@ALI	600	C	128	31.03	55.03	HC	241	-1.03	6.50	H	1024	-4.79	14.35				
2B3W	HCcoNH_@ALI	600	N	128	100.10	134.66	H	128	-1.46	7.04	HN	375	4.98	11.99				
2B3W	HNCA	600	N	128	100.10	134.66	C	128	41.06	71.06	HN	375	4.99	12.00				
2B3W	HNCO	600	N	128	100.10	134.66	C	64	167.04	180.97	HN	375	4.99	11.99				
2B3W	HNHA	600	N	128	99.90	136.10	H	256	2.50	10.84	HN	375	4.99	11.99				
2B3W	HNcoCA	600	N	128	100.09	134.65	C	128	41.97	71.97	HN	375	4.99	12.00				
2B3W	N15HSQC	750	N	256	99.86	136.07	H	385	4.98	12.01								
2B3W	N15NOESY	750	N	128	99.91	136.12	HN	385	4.98	12.01	H	512	-1.89	11.46				
KRAS	C13HSQC_@ALI	700	C	1002	4.24	72.74	H	2206	-1.15	6.39								
KRAS	C13HSQC_@ARO	600	C	313	109.38	134.67	H	578	5.71	7.69								
KRAS	C13NOESY_@ALI	700	C	256	0.19	75.19	HC	1046	-0.87	6.42	H	430	-0.91	10.85				
KRAS	C13NOESY_@ARO	700	C	256	0.39	75.39	HC	272	6.17	8.06	H	392	-0.91	9.81				
KRAS	CBCANH	700	N	128	98.75	134.75	C	256	4.73	74.73	HN	2048	4.69	11.69				
KRAS	CBCAcoNH	700	N	128	98.76	134.76	C	256	4.40	74.40	HN	2048	4.70	11.70				
KRAS	CcoNH	700	N	256	98.69	134.69	C	512	2.69	74.69	HN	1024	4.68	11.69				
KRAS	CcoNH_2	700	N	256	99.00	135.00	C	512	3.00	75.00	HN	1024	4.70	11.70				
KRAS	HBHAcoNH	700	N	256	98.73	134.73	HN	723	6.14	10.99	H	519	-0.52	6.57				
KRAS	HCCHTOCSY_@ALI	700	C	256	-0.82	74.18	HC	307	-1.51	6.88	H	1060	-0.80	6.45				
KRAS	HNCA	700	N	128	98.73	134.73	C	256	37.70	69.70	HN	2048	4.70	11.70				
KRAS	HNcoCA	700	N	128	98.75	134.75	C	256	37.70	69.70	HN	2048	4.70	11.70				
KRAS	N15HSQC	700	N	988	99.16	133.90	H	1425	6.08	10.95								
KRAS	N15NOESY_@ALI	700	N	256	96.97	136.46	HN	342	6.06	10.83	H	419	-0.67	10.79				
2G0Q	C13NOESY	600	C	128	22.54	63.97	HC	1024	-3.54	13.13	H	256	-2.15	11.74				
2G0Q	C13NOESY_@ARO	600	C	128	110.44	143.59	HC	512	4.79	13.13	H	256	-2.15	11.74				
2G0Q	CBCANH	600	N	128	102.38	135.28	C	128	1.08	83.95	HN	512	4.81	13.14				
2G0Q	CcoNH	600	N	128	102.64	135.54	C	128	1.91	84.78	HN	512	4.81	13.14				
2G0Q	HBHAcoNH	600	N	128	102.38	135.28	HN	512	4.81	13.14	H	128	-1.09	7.25				
2G0Q	HCCHTOCSY_@ALI	600	C	128	22.56	63.99	HC	256	-1.09	7.24	H	512	-1.09	7.24				
2G0Q	HNCA	600	N	128	102.38	135.28	C	128	33.57	75.00	HN	512	4.81	13.14				
2G0Q	HNCO	600	N	128	102.38	135.28	C	128	169.43	182.69	HN	512	4.81	13.14				
2G0Q	HNcaCO	600	N	128	102.38	135.28	C	128	169.43	182.69	HN	512	4.81	13.14				
2G0Q	HNcoCA	600	N	128	102.38	135.28	C	128	33.54	74.97	HN	512	4.81	13.14				
2G0Q	N15HSQC	600	N	1024	102.65	135.55	H	1024	4.81	13.14								
2G0Q	N15NOESY	600	N	128	102.66	135.56	HN	512	4.81	13.14	H	256	-2.14	11.75				
2LF2	C13HSQC_@ALI	850	C	256	5.80	85.80	H	527	-1.52	6.20								
2LF2	C13HSQC_@ARO	850	C	256	108.00	142.00	H	274	5.48	9.50								
2LF2	C13NOESY_@ALI	850	C	512	11.80	75.80	HC	547	-1.52	6.49	H	256	-1.47	11.03				
2LF2	C13NOESY_@ARO	850	C	256	111.26	141.27	HC	274	5.48	9.50	H	256	-1.47	11.03				
2LF2	CBCANH	600	N	256	102.93	132.93	C	256	14.00	78.00	HN	342	5.48	11.51				
2LF2	CBCAcoNH	600	N	256	102.93	132.93	C	128	13.50	78.50	HN	370	5.48	12.00				
2LF2	CCHTOCSY_@ALI	600	C1	256	4.58	70.58	C2	256	4.58	70.58	H1	399	-1.03	5.98				
2LF2	CCNOESY_@ALI	600	C1	32	13.29	33.99	C2	32	13.29	33.99	H1	1024	-3.70	9.64	H2	128	-0.53	6.47
2LF2	CcoNH	600	N	256	102.93	132.93	C	128	14.00	78.00	HN	370	5.48	12.00				
2LF2	HBHAcoNH	600	N	256	102.93	132.93	HN	370	5.48	12.00	H	256	0.02	7.02				
2LF2	HCCHCOSY_@ALI	600	C	256	8.08	78.08	HC	256	-0.71	6.29	H	427	-1.01	6.51				
2LF2	HCCHTOCSY_@ALI	600	C	256	8.08	78.09	HC	256	-0.71	6.29	H	427	-1.01	6.50				
2LF2	HCcoNH_@ALI	600	N	256	102.93	132.93	H	256	-0.72	6.28	HN	370	5.48	12.00				
2LF2	HNCA	600	N	256	102.93	132.93	C	128	41.07	71.07	HN	371	5.48	12.01				
2LF2	HNCO	600	N	256	102.93	132.93	C	128	167.97	183.97	HN	371	5.48	12.00				
2LF2	HNcoCA	600	N	256	102.93	132.93	C	128	41.00	71.00	HN	371	5.48	12.01				
2LF2	N15HSQC	600	N	1024	104.92	132.92	H	370	5.48	12.00								
2LF2	N15NOESY	850	N	128	104.00	132.00	HN	372	5.48	11.99	H	512	-1.47	11.03				

Supplementary Table 5. Accuracy of AlphaFold models and ARTINA structures.

ID	Protein	Residues	Backbone RMSD to PDB structure [Å]		ID	Protein	Residues	Backbone RMSD to PDB structure [Å]	
			AlphaFold structure	ARTINA structure				AlphaFold structure	ARTINA structure
1	6SVC	35	1.25	0.83	51	2KIF	108	1.04	0.89
2	2JVD	54	0.32	0.71	52	2KBN	109	0.72	0.92
3	2K57	55	0.48	0.71	53	2MK2	109	1.22	1.56
4	6SOW	58	0.7	1.16	54	2K50	110	0.55	1.00
5	2LX7	60	2.26	1.41	55	2KL5	110	1.31	2.58
6	2MA6	61	0.7	1.56	56	2LTA	110	0.98	2.39
7	2JRM	65	1.3	1.43	57	2KIW	111	1.29	1.59
8	1YEZ	68	0.54	0.73	58	2LVB	112	2.66	1.56
9	2L9R	69	0.39	0.59	59	2LND	112	2.73	0.85
10	2K52	74	0.95	1.10	60	1WQU	114	0.67	0.98
11	2KRS	74	0.72	1.26	61	2KL6	114	0.62	0.87
12	2K53	76	0.57	0.88	62	6GT7	115	0.92	1.39
13	2JT1	77	0.54	0.94	63	2JN8	115	1.38	1.83
14	2JVO	77	1.41	1.77	64	2K5D	116	1.02	1.47
15	2ERR	81	1.51	2.09	65	2KD1	118	0.58	1.99
16	2L1P	83	1.4	2.13	66	2LTL	119	0.74	2.37
17	2LN3	83	0.54	0.89	67	2KVO	120	1.87	1.87
18	2HEQ	84	0.51	0.60	68	1T0Y	120	0.91	1.27
19	2KK8	84	0.96	1.25	69	2KCD	120	1.38	3.13
20	2KD0	85	0.81	1.37	70	2KRT	121	1.86	2.09
21	2LML	86	0.57	1.53	71	2LFI	122	1.66	2.42
22	2K3D	87	1.07	1.44	72	2JQN	122	1.36	1.52
23	2LK2	89	0.74	1.42	73	2L7Q	124	1.03	1.57
24	MH04	90	1.12	1.57	74	2KFP	125	1.42	2.23
25	1PQX	91	1.09	1.40	75	1SE9	126	1.64	2.10
26	2L33	91	0.61	0.79	76	2L3G	126	0.43	1.28
27	2KZV	92	1.24	2.62	77	2L3B	130	0.76	1.05
28	2KCT	94	0.4	0.77	78	2LRH	134	1.57	2.30
29	2MDR	94	1.32	1.72	79	1VEE	134	0.6	1.03
30	2FB7	95	1.14	1.94	80	2K1G	136	0.53	1.05
31	2MB0	95	0.63	1.11	81	2KKZ	140	0.72	1.47
32	2L05	95	0.58	0.74	82	1VDY	140	0.86	0.95
33	2KJR	95	1.28	1.02	83	2KKL	140	1.43	1.25
34	2M5O	97	0.57	1.08	84	2N4B	142	0.99	1.14
35	MDM2	97	1.3	1.24	85	2L8V	143	1.29	2.79
36	2LNA	99	0.59	0.86	86	2LGH	144	0.7	2.43
37	2LA6	99	0.43	0.81	87	2K1S	149	1.03	1.83
38	6FIP	99	2.13	2.05	88	2M4F	151	0.73	1.11
39	2LEA	100	1.13	1.45	89	2JXP	155	1.48	2.58
40	2LL8	101	0.7	1.42	90	2L06	155	1.08	1.57
41	2KPN	103	0.57	0.97	91	2LAH	160	1.01	1.71
42	2K0M	104	0.77	1.60	92	2LAK	160	0.73	1.54
43	2K5V	104	0.48	0.94	93	2L82	162	1.86	3.55
44	2MQL	105	0.78	0.98	94	2M47	163	1.93	4.72
45	2K75	106	0.92	1.65	95	2K3A	163	0.69	0.99
46	2LTM	107	0.44	0.67	96	2M7U	165	1.49	2.14
47	2KOB	108	0.56	2.24	97	2B3W	168	1.11	2.67
48	2KHD	108	1.06	1.87	98	KRAS4B	169	1.25	1.60
49	2RN7	108	0.71	0.83	99	2G0Q	173	1.01	2.38
50	2LXU	108	0.62	1.19	100	2LF2	175	1.88	2.68

RMSDs are calculated for the backbone atoms N, C^{α}, C’ in the residue ranges given in Supplementary Table 4.

Supplementary Table 6. Structure recalculation with CYANA from deposited restraints.

ID	Protein				Restraints				PDB structure statistics			Recalculated structure		
	PDB code[a]	Residues in PDB structure[b]	Residues included in RMSD calculation	Residue range for RMSD calculation[c]	Format in PDB	NOE	H-bonds	Torsion angles	CYANA target function [Å^2]	CYANA target function for NMR data [Å^2][d]	Backbone RMSD to mean [Å]	CYANA target function [Å^2]	Backbone RMSD to mean [Å]	Backbone RMSD to PDB structure [Å]
1	6SVC	35	23	7-29	unknown	–	–	–	0.83	–	0.37	–	–	–
2	2JVD	48	34	4-37	xplor	1051	48	68	1.78	0.02	0.28	0.01	0.30	0.43
3	2K57	55	48	5-52	xplor	1051	0	133	23.33	21.29	0.36	0.18	0.32	0.71
4	6SOW	58	48	8-55	cyana	1163	0	0	1.01	0.66	0.25	0.38	0.20	0.48
5	2LX7	60	55	5-59	xplor	427	0	80	2.11	0.18	1.35	0.02	1.30	1.02
6	2MA6	61	48	10-57	xplor	777	32	52	2.41	0.19	0.44	0.50	0.65	1.02
7	2JRM	60	42	6-47	xplor	2120	100	308	6.45	1.28	0.34	2.44	0.18	0.61
8	1YEZ	68	49	15-25, 29-66	xplor	1237	0	0	4.81	0.22	0.42	0.04	0.56	0.76
9	2L9R	69	44	13-56	xplor	1534	38	140	1.95	0.16	0.29	0.28	0.29	0.55
10	2K52	74	64	7-70	xplor	1107	0	166	7.92	3.88	0.40	0.26	0.41	0.94
11	2KRS	74	60	2-61	xplor	939	36	104	5.69	0.11	0.40	0.01	0.43	0.50
12	2K53	70	49	8-28, 39-66	xplor	932	0	0	3.75	0.17	0.29	0.36	0.22	0.71
13	2JT1	71	57	5-57, 66-69	xplor	1224	52	92	3.08	0.03	0.61	0.02	0.50	0.72
14	2JVO	77	66	6-71	amber	874	54	0	6.43	3.61	0.32	14.27	0.49	0.76
15	2ERR	88	74	2-75	amber	594	10	0	1.21	0.37	0.37	3.99	0.73	1.02
16	2L1P	83	60	19-78	xplor	1190	0	90	5.19	0.01	1.17	0.38	1.29	1.29
17	2LN3	83	67	6-72	xplor	2847	70	108	2.69	0.28	0.43	0.01	0.39	0.56
18	2HEQ	84	39	17-20, 34-68	xplor	507	30	53	7.13	0.00	0.40	0.21	0.36	0.65
19	2KK8	84	73	10-82	xplor	1461	0	91	5.46	0.24	0.63	0.09	0.74	1.12
20	2KD0	85	69	13-81	xplor	1371	0	85	3.33	0.02	0.55	0.35	0.69	1.01
21	2LML	87	76	3-78	xplor	1110	54	101	3.27	0.34	0.51	0.40	0.78	1.30
22	2K3D	87	80	2-81	xplor	1075	0	105	7.48	0.15	0.76	0.03	0.73	0.84
23	2LK2	89	52	14-65	xplor	860	72	90	10.43	0.18	0.70	0.31	0.61	0.95
24	MH04	–	82	6-87	–	–	–	–	–	–	–	–	–	–
25	1PQX	91	54	13-19, 28-34, 38-65, 70-81	xplor	1490	54	179	13.11	0.30	0.36	0.57	0.72	1.14
26	2L33	91	52	19-36, 46-79	xplor	2529	48	84	3.76	0.08	0.31	0.03	0.28	0.43
27	2KZV	92	72	9-80	xplor	863	58	103	8.54	0.38	1.01	0.07	0.94	1.16
28	2KCT	88	67	11-37, 44-83	xplor	1437	0	79	2.46	0.26	0.57	0.22	0.54	0.98
29	2MDR	94	81	9-89	xplor	2165	98	0	7.08	0.25	0.40	0.64	0.32	0.57
30	2FB7	80	47	20-52, 74-87	xplor	1029	40	85	34.23	28.37	0.62	3.14	0.36	0.45
31	2MB0	95	71	8-41, 49-85	amber	2344	30	0	6.72	2.57	0.15	10.18	0.21	0.65
32	2L05	86	71	19-89	xplor	1817	0	125	3.74	0.05	0.54	0.18	0.53	0.81
33	2KJR	95	76	15-23, 28-94	xplor	1362	62	112	7.25	0.24	0.37	0.01	0.39	0.69
34	2M5O	97	75	17-91	xplor	2992	46	120	2.83	0.27	0.37	0.59	0.38	0.75
35	MDM2	–	84	9-92	–	–	–	–	–	–	–	–	–	–
36	2LNA	99	70	16-49, 59-94	xplor	1352	70	120	3.08	0.35	0.54	0.37	0.46	0.90
37	2LA6	99	83	15-97	xplor	2888	30	80	2.65	0.43	0.36	4.62	0.43	0.79
38	6FIP	99	83	11-93	cyana	1698	0	0	0.51	0.15	0.57	0.26	0.61	0.86
39	2LEA	101	65	15-45, 55-88	amber	2623	54	0	6.02	1.69	0.14	9.29	0.16	0.35
40	2LL8	101	87	4-90	xplor	1921	94	144	2.89	0.29	0.47	0.36	0.50	0.94
41	2KPN	97	73	12-84	xplor	1865	0	132	2.97	0.08	0.43	0.42	0.32	0.76
42	2K0M	104	82	7-70, 76-93	xplor	1834	0	0	5.54	0.06	0.56	0.12	0.86	0.75
43	2K5V	98	81	2-29, 38-78, 83-94	xplor	1792	0	124	5.32	1.99	0.50	0.04	0.48	1.08
44	2MQL	105	70	15-84	cyana	2440	28	0	2.28	0.33	0.14	0.58	0.43	0.53
45	2K75	106	90	3-92	xplor	1313	90	130	7.63	0.11	0.39	0.05	0.56	0.91
46	2LTM	107	86	14-99	xplor	2943	50	112	3.02	0.34	0.42	0.42	0.38	0.81
47	2KOB	108	91	3-93	xplor	1659	100	178	6.27	0.14	0.51	0.56	0.49	0.99
48	2KHD	108	67	31-97	xplor	829	78	110	9.35	0.25	0.56	0.02	0.52	1.15
49	2RN7	108	46	10-55	xplor	0	0	0	0.83	0.01	0.55	0.28	0.41	0.52
50	2LXU	108	87	9-95	xplor	1051	48	68	1.78	0.31	0.61	0.06	0.60	0.73

ID	Protein				Restraints				PDB structure statistics			Recalculated structure		
	PDB code[a]	Residues in PDB structure[b]	Residues included in RMSD calculation	Residue range for RMSD calculation[c]	Format in PDB	NOE	H-bonds	Torsion angles	CYANA target function [Å²]	CYANA target function for NMR data [Å²][d]	Backbone RMSD to mean [Å]	CYANA target function [Å²]	Backbone RMSD to mean [Å]	Backbone RMSD to PDB structure [Å]
51	2KIF	102	95	3-97	xplor	2521	62	125	4.87	0.12	0.47	0.49	0.53	0.72
52	2KBN	109	79	5-29, 34-54, 58-76, 81-94	xplor	1452	84	80	9.72	0.20	0.43	0.12	0.50	0.40
53	2MK2	109	95	14-108	xplor	1357	46	108	4.30	0.50	1.01	0.05	0.94	1.17
54	2K50	110	83	10-34, 42-85, 92-105	xplor	2030	0	214	27.01	22.16	0.37	0.31	0.41	0.93
55	2KL5	110	69	12-53, 58-66, 76-86, 93-99	xplor	2422	31	0	8.34	0.08	0.49	0.06	0.57	0.68
56	2LTA	110	95	4-98	xplor	3183	38	164	4.26	0.45	0.59	0.08	0.59	1.00
57	2KIW	111	80	2-81	xplor	1005	96	151	9.39	0.11	0.71	0.07	0.61	1.63
58	2LVB	112	100	3-102	xplor	2994	82	160	3.46	0.36	0.49	0.02	0.42	0.79
59	2LND	112	96	3-48, 52-101	xplor	3367	70	169	3.70	0.37	0.46	0.07	0.45	0.69
60	1WQU	114	99	8-106	cyana	2291	0	0	6.38	5.13	0.45	2.10	0.44	0.49
61	2KL6	108	101	6-106	xplor	2787	0	166	4.04	0.39	0.37	0.64	0.30	0.85
62	6GT7	115	92	7-30, 40-87, 94-113	cyana	2129	92	174	4.06	1.84	0.23	1.68	0.28	0.56
63	2JN8	109	94	12-26, 31-109	xplor	1700	84	128	8.52	0.16	1.07	0.93	1.19	0.78
64	2K5D	110	72	19-50, 55-84, 98-107	xplor	1721	38	99	5.27	0.12	0.61	0.26	0.98	0.70
65	2KD1	118	83	7-89	xplor	2270	0	246	3.58	0.20	0.42	2.05	0.35	1.23
66	2LTL	119	85	19-35, 39-41, 46-110	xplor	2501	40	122	3.34	0.21	0.56	0.30	0.59	1.12
67	2KVO	120	97	3-23, 28-103	xplor	1447	64	91	12.29	0.29	0.80	0.39	0.98	0.73
68	1T0Y	90	80	4-83	xplor	1732	0	0	7.97	0.80	0.68	1.19	0.78	0.88
69	2KCD	120	106	3-108	xplor	1133	84	136	13.21	0.37	0.80	0.14	0.88	0.99
70	2KRT	121	109	6-114	xplor	2310	0	128	6.68	0.06	0.71	0.09	0.85	1.30
71	2LFI	122	103	2-104	xplor	2031	78	157	5.69	1.37	1.02	0.37	0.99	1.21
72	2JQN	116	109	3-111	xplor	1676	66	108	5.43	0.04	1.15	0.30	1.32	1.18
73	2L7Q	124	92	12-37, 46-101, 105-114	xplor	1263	68	98	9.47	0.13	0.85	0.06	0.76	0.76
74	2KFP	125	113	3-115	xplor	1819	80	166	13.12	0.78	0.75	0.71	0.97	0.71
75	1SE9	101	76	17-84, 94-101	unknown	–	–	–	5.24	–	0.74	–	–	–
76	2L3G	126	111	13-123	xplor	3540	54	150	4.61	0.07	0.39	0.10	0.40	0.65
77	2L3B	130	94	14-38, 45-113	xplor	1354	78	151	9.24	0.20	0.73	0.12	0.64	0.66
78	2LRH	134	120	3-122	xplor	4405	60	224	5.78	0.55	0.56	0.07	0.47	0.91
79	1VEE	134	118	6-123	cyana	3043	0	0	3.35	1.16	0.42	1.89	0.36	0.38
80	2K1G	129	114	5-78, 83-122	xplor	2626	48	138	7.70	0.14	0.54	0.08	0.58	0.77
81	2KKZ	134	109	5-80, 86-118	xplor	2377	0	203	5.40	0.48	0.48	0.18	0.52	1.17
82	1VDY	140	110	9-102, 113-128	cyana	3348	0	0	3.44	1.23	0.40	1.62	0.39	0.45
83	2KKL	140	88	33-90, 96-125	xplor	1503	44	84	13.04	0.43	0.72	0.28	0.81	0.50
84	2N4B	138	109	2-26, 40-54, 66-134	rosetta	173	0	0	3.26	0.00	0.68	–	–	–
85	2L8V	143	105	4-22, 37-65, 73-129	xplor	1282	96	191	11.06	0.15	1.05	0.26	0.83	1.02
86	2LGH	144	131	2-109, 113-135	xplor	1582	122	238	5.33	0.41	0.74	0.40	0.62	1.37
87	2K1S	149	138	3-140	xplor	1114	110	195	9.62	0.10	0.74	0.09	0.88	0.94
88	2M4F	151	98	23-46, 51-57, 63-94, 103-114, 120-129, 136-148	cyana	3446	0	0	1.36	0.77	0.21	0.95	0.28	0.25
89	2JXP	155	129	16-144	xplor	2279	0	0	7.86	0.05	0.84	0.13	1.51	1.98
90	2L06	155	121	15-38, 45-141	xplor	1790	116	194	10.72	0.21	0.85	0.79	0.91	0.77
91	2LAH	160	138	14-25, 33-158	xplor	4891	82	220	6.43	0.74	0.42	1.40	0.45	0.83
92	2LAK	160	85	10-37, 68-77, 93-139	xplor	1481	80	138	14.47	0.31	0.71	0.50	0.75	0.55
93	2L82	162	149	3-151	xplor	5066	82	240	9.80	0.30	0.48	0.14	0.46	0.81
94	2M47	163	129	5-25, 40-56, 66-156	xplor	1575	108	214	5.04	0.51	1.08	0.71	1.11	1.09
95	2K3A	155	82	57-102, 108-127, 138-153	xplor	1874	3	0	4.92	0.68	0.47	0.08	0.72	0.75
96	2M7U	165	140	12-151	xplor	0	0	290	37.75	2.97	0.23	–	–	–
97	2B3W	168	147	16-162	xplor	1291	93	206	16.50	0.01	0.87	0.65	0.92	1.01
98	KRAS	–	104	18-54, 60-126		–	–	–	–	–	–	–	–	–
99	2G0Q	149	104	6-44, 53-69, 76-105, 111-165	xplor	1953	0	0	10.80	0.83	0.53	1.52	0.64	0.91
100	2LF2	175	141	3-97	xplor	1893	166	214	15.46	0.38	0.94	0.17	1.07	1.24

[a]Proteins without PDB deposition are identified by their abbreviated name (MH04, MDM2, KRAS). No structure recalculation was carried out for these. [b]Number of residues with coordinates in the deposited PDB structure. [c]Residue ranges for RMSD calculation were determined by CYRANGE applied to the region between the first residue of the first secondary structure element and the last residue of the final secondary structure element of the reference PDB structure. Residues are numbered as in the BMRB deposition. [d]CYANA target function value for NMR-based restraints, excluding steric lower bounds.

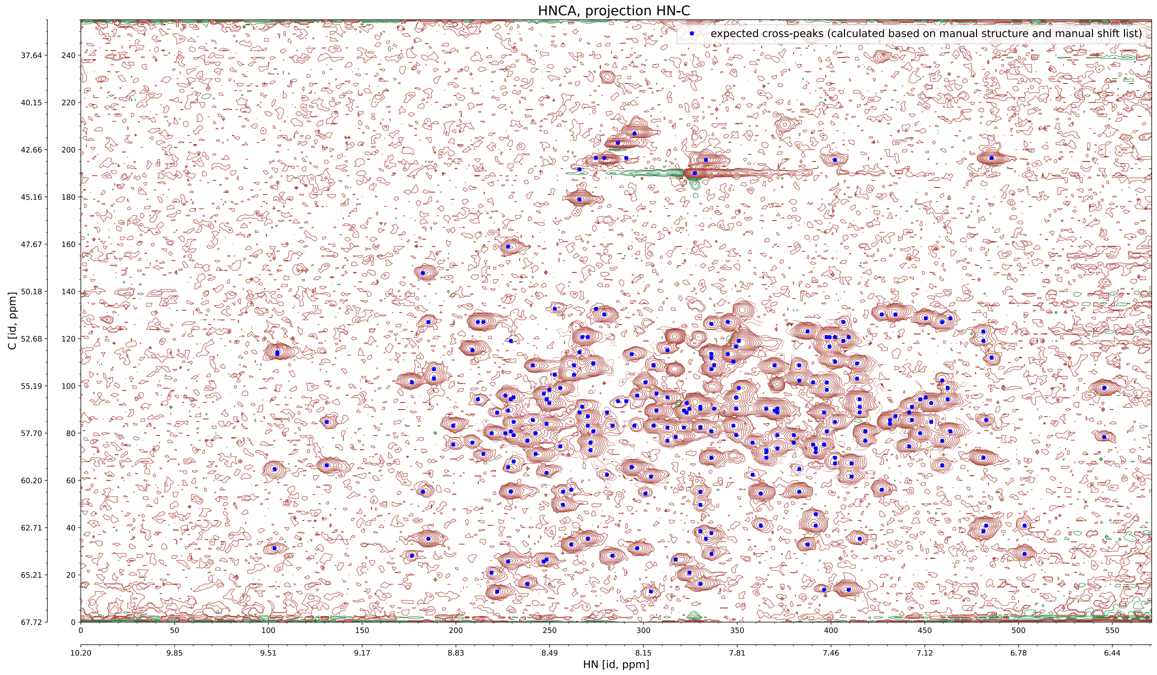

Supplementary Fig. 1. Projection of the HNCA spectrum of the protein 1VDY. Positions of peaks back-calculated from the chemical shifts deposited in the BMRB are indicated by blue dots. A total of 3593 visualizations of this type were generated for all spectra and manually inspected to ascertain the consistency of the ARTINA dataset.

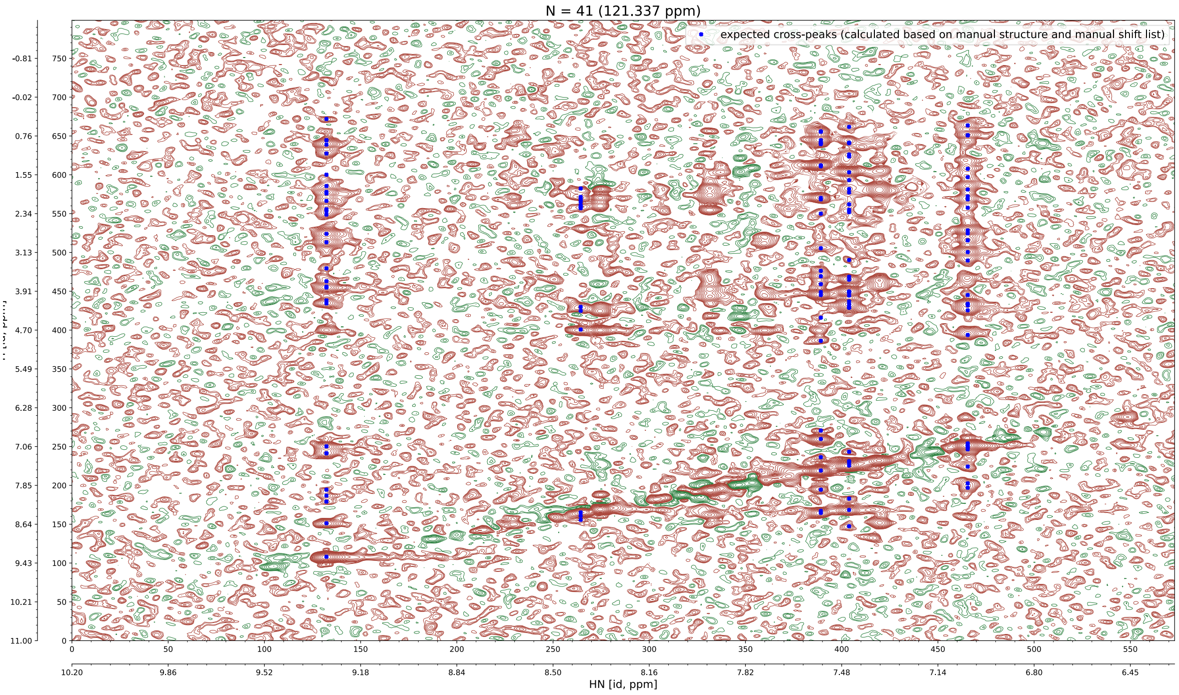

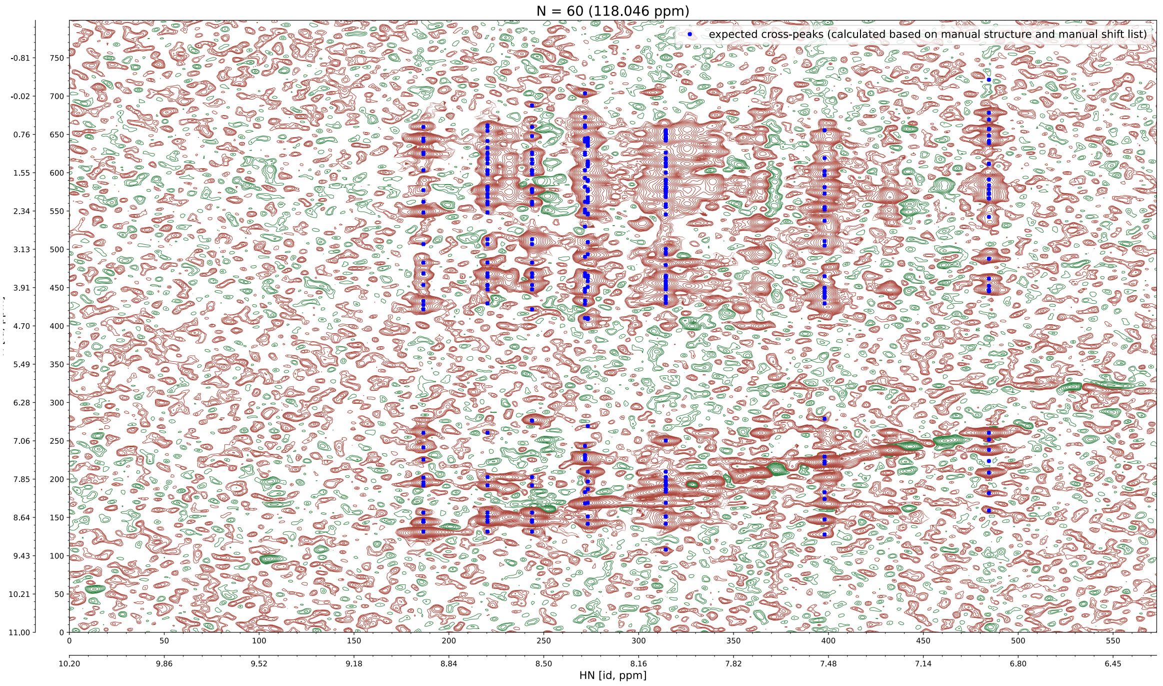

Supplementary Fig. 2. Example 2D planes of the ^{15}N-resolved [^{1}H-^{1}H] NOESY spectrum for the protein 1VDY. Peak positions back-calculated from the chemical shifts deposited in the BMRB and the structure in the PDB are marked by blue crosses. A total of 3279 visualizations of this type were generated for all spectra and manually inspected to ascertain the consistency of the ARTINA dataset.
